# Topographically organized dorsal raphe activity modulates forebrain sensory-motor representations and contributes to defensive behaviors

**DOI:** 10.1038/s41467-026-75490-y

**Published:** 2026-07-16

**Authors:** Aytac Kadir Mutlu, Bram Serneels, Christoph Wiest, Anh-Tuan Trinh, Ricarda Bardenhewer, Fabrizio Palumbo, Oda Bjørnevik Frisvold, Inger Kristine Fjeldskaar Aukrust, Anna Maria Ostenrath, Emre Yaksi

**Affiliations:** 1 Kavli Institute for Systems Neuroscience and Centre for Neural Computation, Faculty of Medicine and Health Sciences, Norwegian University of Science and Technology, Olav Kyrres gata 9, Trondheim, Norway; 2 National Hospital for Neurology and Neurosurgery, University College London Hospitals NHS Foundation Trust, London, UK; 3 Koç University Research Center for Translational Medicine, Koç University School of Medicine, Istanbul, Turkey

**Keywords:** Neural circuits, Sensory processing, Sensorimotor processing

## Abstract

The dorsal raphe nucleus (DRN) shapes behaviors including mood and motivation. The DRN contains molecularly distinct and topographically organized neurons that target specific forebrain regions. To understand how DRN neurons process sensory information, we investigated the spatiotemporal activity patterns of DRN neurons, DRN axons, and their forebrain targets in zebrafish. We found a remarkable topographic organization of ongoing activity and sensory-motor responses within the DRN. A subset of DRN neurons was driven by locomotion and sensory stimuli. Gad1-positive DRN neurons exhibited distinct activity during rest and sensory-motor stimulation. DRN axons in the forebrain showed topographically organized excitation and inhibition in response to sensory stimulation and locomotion. DRN axons covaried with forebrain neuronal activity. DRN ablation reduced the synchrony and sensory-motor responses of forebrain neurons and enhanced defensive behaviors. We revealed the functional diversity of DRN neurons and their role in transmitting sensory and locomotor signals via topographically organized forebrain projections.

## Abstract (web summary)

Functional heterogeneity of dorsal raphe nucleus is not fully understood. Here authors show topographically organized sensory and locomotor responses from the zebrafish dorsal raphe and its forebrain projections. Dorsal raphe ablation disrupts forebrain synchrony, weakens sensory and motor responses, and enhances defensive behaviors.

## Introduction

Neuromodulatory systems are essential in shaping brain computations and animal behavior by dynamically regulating neural circuit activity^1–16^. Serotonin is a critical neuromodulator that is evolutionarily conserved across species^17–25^. Various serotonin receptors are widely expressed throughout the nervous system^26–28^, adjusting synaptic transmission^29,30^, neural activity^16,20,31–33^ and orchestrating network dynamics^33,34^, enabling flexible responses to changing internal states and environmental demands. The primary hub of the serotonergic system in the vertebrate brain is the dorsal raphe nucleus (DRN), located in the midbrain^19,20,35–37^. DRN neurons project broadly across the brain, influencing diverse neural processes from sensory and motor computations^11,26,38–40^ to regulation of emotions and mood^4,39,41–43^, appetite^44,45^, sleep^20,46^ as well as higher-order cognitive functions such as learning^16,26,38^ and decision-making^41,47^. Hence, DRN is critical for the brain to correctly integrate sensory cues and adapt behavioral outputs. Consequently, DRN is a primary target for understanding and treating various neuropsychiatric disorders^48,49^.

Recent studies revealed that DRN is not a homogenous nucleus but instead composed of molecularly diverse and topographically organized groups of neurons^36,50–53^. Viral tracing of DRN projections in mice showed that serotonergic neurons located in ventral DRN and co-expressing *vesicular glutamate transporter 3* (*Vglut3*) primarily innervate cortical regions^35,54^, whereas dorsal DRN neurons co-expressing *thyrotropin-releasing hormone/glutamate decarboxylase 1/glutamate decarboxylase 2* (*Trh/Gad1/Gad2*) mainly project to subcortical regions^35,54^. These results suggest that molecularly and topographically distinct subcircuits of DRN neurons may be involved in processing of different neural information and distribute this to distinct forebrain targets. Do these DRN subcircuits represent functionally distinct ensembles of neurons? What kind of environmental and self-generated cues are encoded across DRN neurons? How is this information transmitted by DRN axons in the forebrain, targeting cortical and subcortical structures? And finally, what role do DRN inputs play in orchestrating and regulating the activity of neural ensembles across the forebrain?

We aimed to answer these questions by using a small vertebrate, zebrafish, with relatively transparent brains that enable the monitoring of activity in large populations of individual neurons^4,7,19,38,40,55–59^ across DRN^19,20,26,38,46^ and the forebrain^60,61^. At the juvenile stage, around 3–4 weeks, zebrafish begin to exhibit cognitively demanding behaviors such as learning^62–64^, social interactions^61,65,66^, and diverse defensive behaviors^67–69^ that are typically associated with neuromodulation and maturation of the forebrain. Various developmental^70–73^, molecular^70,71^, anatomical^70,71^, behavioral^74,75^, and functional^76–80^ studies increasingly point toward similarities between the zebrafish and the mammalian forebrain. While most studies on the zebrafish DRN^38,40,81,82^ focus on reflex-like midbrain and hindbrain sensory-motor computations, the impact of DRN inputs to ancestral cortico-limbic structures in zebrafish forebrain and DRN’s role in defensive behaviors remains to be explored.

In this study, we examined the activity of DRN neurons and their forebrain projections in awake, behaving juvenile zebrafish. We found that DRN neurons are organized into functional ensembles, where nearby neurons exhibit correlated activity patterns during rest, sensory stimulation, and locomotion. The majority of DRN neurons are significantly modulated by the animal’s locomotor activity, while smaller subsets respond to mechanical vibrations and light. A specific ensemble of Gad1b-expressing DRN neurons, located in the anterio-dorsal DRN, displays coordinated ongoing activity and is preferentially inhibited during sensory stimulation and locomotor responses. Imaging of DRN axons in the forebrain revealed prominent functional topography, with spatially distinct forebrain regions receiving synchronized DRN inputs that are either inhibited or excited during locomotion or sensory stimulation. Calcium signals in DRN axons and forebrain neurons covary, suggesting rapid functional coupling between them. Chemogenetic DRN ablation significantly reduces synchrony across the forebrain, emphasizing the DRN’s potential role as an orchestrator of the forebrain. Furthermore, DRN ablation decreases locomotor and sensory-evoked activation of forebrain neurons. Finally, DRN ablation significantly weakens the relationship between forebrain activity and locomotor events, impairing the animals’ recovery following aversive vibrations and their behavioral adaptation to novel tank.

## Results

### Dorsal raphe nucleus is composed of topographically organized neural ensembles

**Fig. 1:**
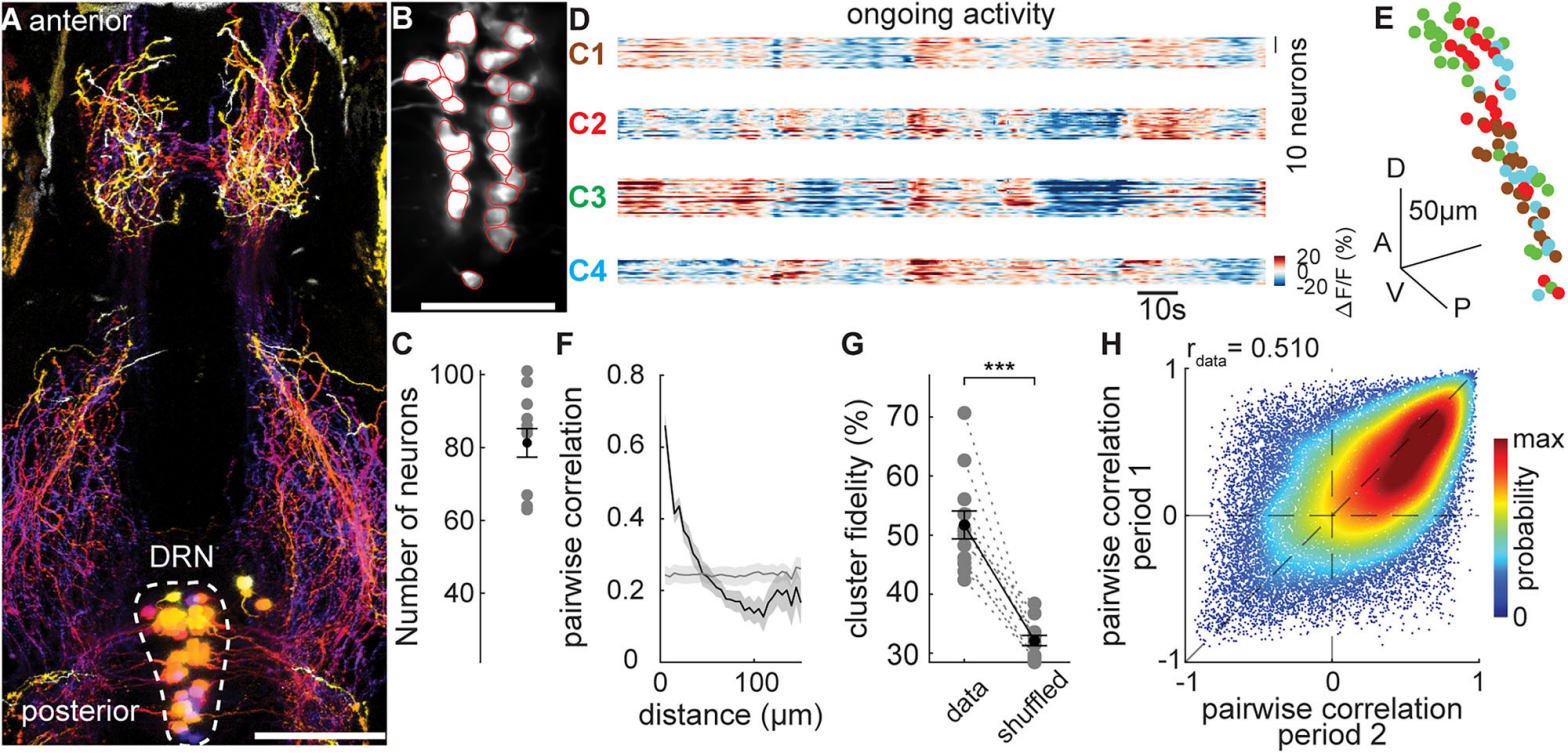
Ongoing activity of dorsal raphe ensembles is topographically organized. **A** Confocal microscopy image of dorsal raphe and its axonal projections in three-week-old *Tg(tph2:Gal4;UAS:caax-GFP)* juvenile zebrafish. Depth is color-coded: dorsal regions warmer and ventral regions colder. Scale bar is 100 μm. **B** Two-photon microscopy image of dorsal raphe neurons (red contour lines) in *Tg(tph2:Gal4;UAS:GCaMP6s)* zebrafish. Scale bar is 50 μm. **C** 81 ± 4 (mean ± SEM) dorsal raphe neurons were recorded in each fish (*n* = 12). **D** Ongoing dorsal raphe activity recorded using two-photon calcium imaging in *Tg(tph2:Gal4;UAS:GCaMP6s)* zebrafish. Dorsal raphe ensembles are clustered (C1–4) using k-means clustering. Warm colors represent higher calcium signals. **E** Three-dimensional reconstruction of dorsal raphe ensembles (k-means functional clusters). Neurons are color-coded based on their cluster identities shown in panel D. A: anterior, P: posterior, D: dorsal, V: ventral. **F** Pairwise Pearson’s correlation of dorsal raphe neurons during ongoing activity as a function of distance (*μm*) between each neuron pair. Light-gray line represents shuffled spatial distribution. Line represents mean, shading represents SEM. **G** The ratio of dorsal raphe neuron pairs remaining in the same functional clusters during two consecutive ongoing activity periods (high cluster fidelity) is significantly higher than chance levels. (*n* = 12 fish). Error bar represents mean ± SEM. ***p = 0.0005, two-sided Wilcoxon signed-rank test. **H** Pairwise correlations of dorsal raphe neuron activity during two consecutive ongoing activity periods. color: probability density estimate. r_data_ = 0.510 indicate robust synchrony between pairs of neurons. Orthogonal dashed lines: zero-correlation lines. Diagonal dashed line: unity line.

DRN neurons were shown to be molecularly diverse and topographically organized^36,50–53^. Hence, we first asked whether one can identify functional subcircuits or neuronal ensembles^83^ within DRN. To do this, we performed volumetric two-photon calcium imaging of the entire DRN in head-restrained^60,67^, awake and behaving juvenile (3 weeks old) *Tg(tph2:Gal4; UAS:GCaMP6s)* zebrafish^40,59,84,85^, expressing GCaMP6s in ~80 DRN neurons labelled by *t*ph2 gene (Fig. 1A-C, *n* = 12). We observed a substantial level of ongoing calcium activity across the entire DRN (Fig. 1D). K-means clustering^60,86,87^ of DRN neurons based on the similarities of their ongoing activity revealed 4 optimal clusters (Fig. S1A, B). We observed that functional clusters of DRN neurons with similar activity (Fig. 1D) were topographically organized into distinct DRN zones (Fig. 1E). To quantify this functional DRN topography further, we plotted the average pairwise correlation of DRN neurons as a function of distance between them^59,67,86–88^. We observed that nearby DRN neurons exhibited more correlated ongoing activity compared to distant DRN neurons (Fig. 1F). Next, we assessed the stability of DRN clusters by quantifying the likelihood that pairs of DRN neurons remain in the same cluster during two consecutive 1,5 minute periods, a measure we termed “cluster fidelity”^60,86,87^. Our analysis revealed that over 50% of DRN neuron pairs remained in the same cluster, a proportion significantly above chance level (Fig. 1G). Cluster fidelity of DRN neurons remained significantly above chance level for up to one hour (Fig. S1D). In line with this, we also observed that correlations between DRN neurons remained stable during consecutive 1,5 minute periods (Fig. 1H), when compared to shuffled controls (Fig. S1C). These findings demonstrate that juvenile zebrafish DRN is composed of functionally heterogenous and topographically organized functional ensembles of neurons.

### Dorsal raphe neurons respond to locomotor and sensory signals

**Fig. 2:**
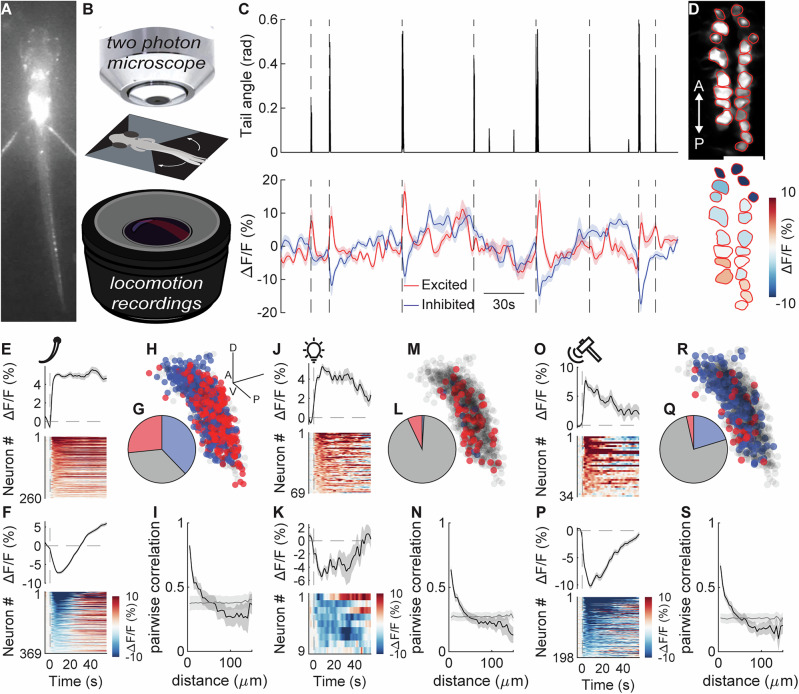
Locomotor and sensory responses of dorsal raphe neurons are topographically organized. **A** Image of a head-restrained and tail-free juvenile zebrafish recorded with an infra-red camera. **B** Scheme of the setup for simultaneous imaging of brain activity and head-restrained zebrafish locomotor behavior. **C** Example trace of locomotion measured as tail-beat angle in head-restrained juvenile zebrafish (top). Simultaneously measured calcium signals (ΔF/F) from dorsal raphe neurons (bottom). Each dashed line marks the onset of a locomotor bout. Line represents mean, shading represents SEM. **D** Two-photon microscope image of dorsal raphe neurons in *Tg(tph2:Gal4;UAS:GCaMP6s)* juvenile zebrafish (top). Average neural response during the first 10 seconds of each tail-bout (bottom). Please note the inhibition (cold colors) in the anterior and the excitation (warm colors) in the posterior raphe. Scale bar represents 25 μm. Dorsal raphe neurons excited (**E**, warm colors) and inhibited (**F**, cold colors) by locomotor tail-bouts. *n* = 12 fish. Line represents mean, shading represents SEM. Fraction (**G**) and spatial distribution (**H**, anterior: A, posterior: P, dorsal:D, ventral: D) of dorsal raphe neurons with significant excitation (red) and inhibition (blue) tail-bout responses. Data from all fish are spatially aligned and overlaid. Scale bar in panel H is 50 μm. **I** Pairwise Pearson’s correlation of dorsal raphe neurons as a function of their distance (*μm*) during tail-bout responses. Gray line represents shuffled spatial distribution. Line represents mean, shading represents SEM. **J**–**N** Same analyses as in panels **E**–**I**, during light-evoked activity (**O**–**S**) Same analyses as in panels **E**–**I**, during mechanical vibration-evoked activity Responses are calculated within 10 s after stimulus onset represented by dashed vertical lines. Horizontal dashed lines: zero-signal lines. Significant responses are calculated if *p* < 0.05 across 6 repetitions. Wilcoxon signed-rank test.

DRN activity is often associated with mood, emotions and internal states^3,6,20,37,38^. We asked whether DRN neurons are sensitive to relatively transient and simpler changes in animal’s locomotor activity or the sensory environment. To test this, we simultaneously measured DRN activity alongside locomotor tail-bouts in head-restrained juvenile zebrafish (Fig. 2A-B, Video S1). We observed that DRN neurons are sensitive to locomotor activity. A population of DRN neurons was excited (in red), while another population was inhibited (in blue) by locomotor tail-bouts (Fig. 2C-D). Across all recorded fish (*n* = 12), 26% ± 24, (mean ± standard deviation) of DRN neurons were excited, and 38% ± 21 were inhibited by locomotor bouts (Fig. 2E-G). Three-dimensional reconstruction of locomotion-modulated DRN neurons across all fish revealed that locomotion-inhibited neurons were predominantly located at the dorsal-anterior DRN zones, while locomotion-excited neurons were positioned toward the ventral-posterior zones (Figs. 2H and S4A-B). To visualize the time courses of locomotor-related DRN responses, we performed k-means clustering. This analysis revealed that DRN ensembles exhibit locomotion-evoked excitation and inhibition with diverse temporal features (Fig. S4M). Interestingly, we also observed a population of DRN neurons that showed initial inhibition followed by excitation (Fig. S4M, green traces). Three-dimensional reconstruction of k-means clusters of locomotor DRN responses confirmed an overall anterior-dorsal vs. posterior-ventral organization (Fig. S4M), consistent with Fig. 2H. To further quantify the topography of locomotion-evoked DRN responses, we calculated pairwise correlations of DRN neuron responses as a function of distance between them^67,86–88^. This analysis revealed significantly higher response correlations between nearby DRN neurons, exceeding chance levels (Fig. 2I). Next, we asked what propotion of ongoing activity in DRN ensembles as in Fig. 1, can be explained by animals’ locomotion. We found that on average only 11.13% ± 0.42 variance of ongoing DRN activity is explained by animals’ locomotion, despite that this value is higher in a fraction of DRN neurons (Fig. S2A). This is also inline with our observation that average rate of DRN calcium activity is not significantly different in transitions between periods of locomotion and quiescence periods without locomotion for at least 30 seconds (Fig. S2B-C). Finally, we also observed that ongoing activity and spatial topography of DRN ensembles are present both during locomotion and quiescence periods without locomotion (Fig. S2D-I). These results showed that while a portion of DRN neurons respond reliably to locomotion, ongoing activity of DRN ensembles is prominent also during locomotory quiescence.

Next, we assed sensory responses in DRN, by delivering relatively neutral red-light flashes and aversive mechanical vibrations (frequency range in Fig. S3A). Red-light flashes did not elicit locomotor responses, whereas mechnial vibrations elicit locomotion in head-restrained animals (Fig. S3B-C-D). We observed that both red-light flashes and mechanical vibrations elicit responses in DRN. While light stimuli elicit primarily excitatory responses in 7% ± 6 of DRN neurons (Figs. 2J-M and S4N), vibrations elicit primarily inhibition in 19% ± 20 of DRN neurons (Figs. 2O-R and S4O). Both light and vibration stimuli, though less prominent compared to the locomotion-related activity, evoked a topographically organized DRN response based on pairwise response correlations of individual neurons (Fig. 2N and S), with increased fraction of vibration-evoked inhibitory responses observed towards the anterior-dorsal DRN (Fig. S4J). We observed weak but not significant enchacement of DRN responses upon repeated stimulation (Fig. S3E). We observed that a fraction of DRN neurons respond to more than one type of stimuli (Fig. S4P–R). Vibration responses of a fraction of DRN neurons were different in those trials with and without locomotory response to vibrations (Fig. S4S), suggesting that locomotion can alter the vibration responses in DRN.

Altogether, these results revealed that DRN neurons are sensitive to rapid changes in animals’ locomotion and environment. Locomotory and sensory responses of DRN neurons are diverse and topographically organized.

### Genetically labeled dorsal raphe neurons expressing Gad1b exhibit distinct ongoing activity and sensory-motor responses

**Fig. 3:**
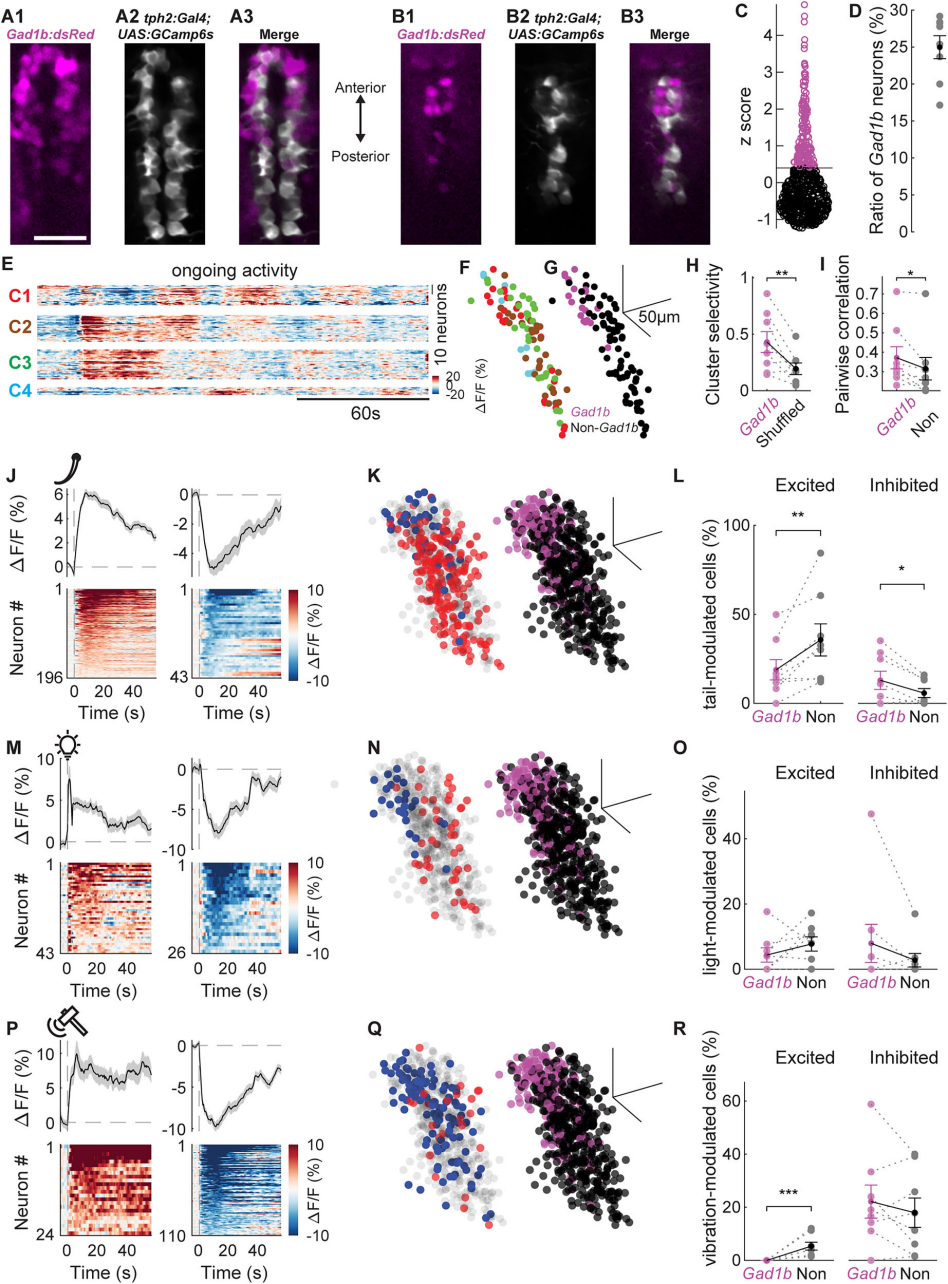
*Gad1b*-labeled dorsal raphe neurons exhibit distinct ongoing activity and sensory-motor responses. **A**, **B** Two-photon images of dorsal raphe in *Tg(Gad1b:dsRed; tph2:Gal4; UAS:GCamp6s)* juvenile zebrafish, dorsal (A1-A3) and ventral (B1-B3) optical planes. *Gad1b:dsRed* in magenta (left), *tph2:Gal4; UAS:GCamp6s* in white (middle) and merged (right). Scale bar 25 μm. **C** Z-scored red fluorescence for every neuron. The horizontal line marks the threshold for *Gad1b:dsRed* positive neurons (magenta). **D** The ratio of *Gad1b:dsRed*-labeled dorsal raphe neurons. **E** Ongoing activity of dorsal raphe neurons in *Tg(Gad1b:dsRed; tph2:Gal4; UAS:GCaMP6s)* zebrafish. Dorsal raphe neurons are k-means clustered (C1–4). Warm colors represent higher calcium signals. **F** Three-dimensional reconstruction of dorsal raphe clusters in panel **E**. **G** *Gad1b:dsRed*-labeling (in magenta) of dorsal raphe neurons in panel **E**. A: anterior, P: posterior, D: dorsal, V: ventral. **H** Cluster selectivity of *Gad1b:dsRed* dorsal raphe neurons (magenta), compared to the same number of random dorsal raphe neurons (gray). *Gad1b:dsRed* dorsal raphe neurons are significantly more selective to functional clusters. **p = 0.0056. **I** Average pairwise correlations of ongoing activity between *Gad1b:dsRed* dorsal raphe neurons (magenta) and across *Gad1b:dsRed* labelled and non-labelled dorsal raphe neurons. *Gad1b:dsRed* dorsal raphe neurons are significantly more correlated among themselves. *p = 0.0138. **J** Tail-bout responses of excited (warm color) and inhibited (cold color) dorsal raphe neurons. Line represents mean, shading represents SEM. **K** Spatial distribution of dorsal raphe neurons (right) that are significantly excited (red) and inhibited (blue) by tail-bouts. Same for *Gad1b:dsRed* dorsal raphe neurons (left). All fish are spatially aligned and overlaid. **L** Fraction of *Gad1b:dsRed* (*Gad1b*), and non-*Gad1b:dsRed* (Non) dorsal raphe neurons that are excited (left) or inhibited (right) by tail-bouts are significantly different. *p = 0.0251, **p = 0.0080. **M**–**O** Same as in panels J-L, during light stimuli. **P**–**R** Same as in panels J-L, during vibration stimuli. ***p = 0.0002. Responses are calculated 20 s after stimulus onset (dashed vertical lines). Horizontal dashed lines mark zero.Significant responses are calculated if *p* < 0.05 (Wilcoxon signed-rank test) across 6 repetitions.*n* = 8 fish. Error bar represents mean ± SEM. *p*-values are calculated using linear mixed-effects model.

In rodents, a genetically distinct group of neurons co-expressing *Trh/Gad1/Gad2* genes are located in the dorsal DRN and projects primarily to subcortical regions^35^. We asked whether juvenile zebrafish DRN contains such *Gad1*-positive neurons and whether these neurons form a distinct functional ensemble. We observed that 25% ± 4 of DRN neurons in the juvenile zebrafish are double-labeled with *Tg(Gad1b:dsRed)*^89^ and *Tg(tph2:Gal4;UAS:GCamp6s)* expression (in magenta and white, Fig. 3A-D). These *Gad1b*-labeled DRN neurons are primarily located in the dorsal-anterior zones (Fig. 3A-B-G).

To explore how *Gad1b*-labeled DRN neurons relate to the topographically organized functional clusters within the DRN (Figs. 1–2), we simultaneously imaged genetic identity and neural activity in the red and green spectra during ongoing activity, locomotion, and sensory stimulation. During ongoing activity, we observed that individual k-means clusters of DRN ensembles (Fig. 3E-F, red ensemble) largely overlap with *Gad1b*-labeled neurons (Fig. 3G, magenta; Fig. S5A). To quantify this overlap, we used a “cluster selectivity” index^60,86,87^. Cluster selectivity would be “0” if *Gad1b* neurons were distributed equally across all DRN ensembles, and “1” if all *Gad1b* neurons are in a single ensemble. We observed that *Gad1b*-labeled DRN neurons showed significantly higher cluster selectivity as compared to chance levels (Fig. 3H), suggesting they are more likely to form a functional ensemble than by chance. Consistently, we also observed significantly higher correlations between *Gad1b* neurons than correlations between *Gad1b* neurons with non-*Gad1b* DRN neurons (Fig. 3I). Next, we asked whether *Gad1b*-labeled DRN neurons have distinct locomotor and sensory responses. We observed that a significantly smaller fraction of *Gad1b* neurons were excited, while a significantly higher fraction of *Gad1b* neurons were inhibited by locomotion (Fig. 3J-L and S5B), when compared to other DRN neurons. We also observed similar trends for sensory responses, where *Gad1b* neurons were largely inhibited by visual and vibration stimulation (Figs. 3M-R and S5C-D). Notably, no *Gad1b* neuron was excited by vibrations (Figs. 3R and S5D). Taken together, our results demonstrate that G*ad1b*-labeled DRN neurons form topographically organized ensembles with preferentially inhibitory responses to locomotor activity and sensory stimulation.

### Forebrain innervations of dorsal raphe exhibit topographically organized activity

**Fig. 4:**
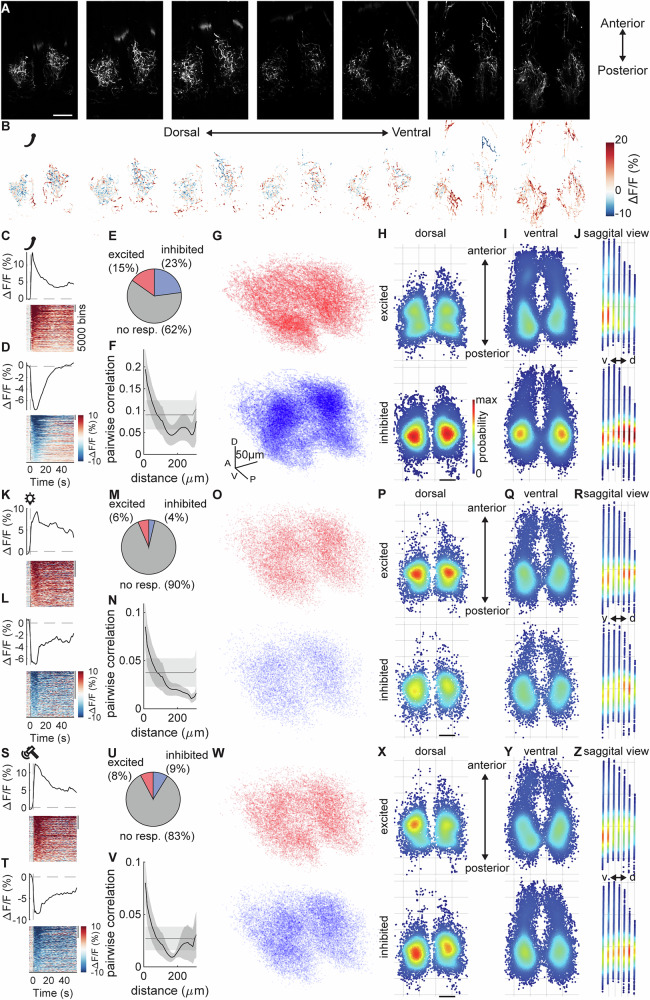
Sensory-motor responses of dorsal raphe axons innervating zebrafish forebrain are topographically organized. **A** Two-photon microscopy images of dorsal raphe axons innervating the forebrain in *Tg(tph2:Gal4;UAS:GCaMP6s)* juvenile zebrafish. Optical sections are shown from dorsal to ventral planes. A: anterior; P: posterior. Scale bar is 100 μm. **B** Spatial distribution of locomotion responses (ΔF/F) of dorsal raphe axons in the forebrain. Warm colors indicate excitation, color colors indicate inhibition. Corresponding optical planes from panel **A**. **C**, **D** Locomotory tail-bout responses of excited (warm color) and inhibited (cold color) dorsal raphe axons in the forebrain. Line represents mean, shading represents SEM. **E** Fraction of dorsal raphe axons with significant excitation (red) and inhibition (blue) during locomotion. **F** Pairwise Pearson’s correlation of dorsal raphe axons as a function of their distance (*μm*) during locomotion. Gray line represents shuffled spatial distribution. Line represents mean, shading represents SEM. **G** Three-dimensional reconstruction of dorsal raphe axons that are significantly excited (red, top) and inhibited (blue, bottom) by locomotory tail-bouts. Significant responses are calculated per axonal bin across 6 repetitions of sensory stimuli and all locomotor tail-bouts (if *p* < 0.05, Wilcoxon signed-rank test). Data from all fish are spatially aligned based on forebrain boundaries and overlaid. Spatial distribution of the locomotion excited (top) and inhibited (bottom) axonal bins in dorsal (**H**) and ventral (**I**) forebrain. Two-dimensional histograms are calculated using ks-density. Warm colors represent higher density/probability of tail-bout modulated axons. **J** Saggital views for the spatial distribution of the locomotion excited (top) and inhibited (bottom) dorsal raphe axonal bins. Warm colors represent higher density/probability of tail-bout modulated axons. v: ventral, d: dorsal. **K**–**R** Same analyses as in panels **C**–**J**, during light-evoked activity. **S**–**Z** Same analyses as in panels **C**–**J**, during mechanical vibration-evoked activity. Responses are calculated within 10 s after stimulus onset represented by dashed vertical lines. Horizontal dashed lines: zero-signal lines. Scale bars are 50 μm. *n* = 14 fish.

Axonal projections of DRN neurons broadly innervate the vertebrate forebrain^35,54,90,91^. However, the type of information carried via these DRN axons and the functional topography of these projections remain to be understood. To investigate this, we measured volumetric calcium signals from DRN axons across the entire forebrain of juvenile zebrafish *Tg(tph2:Gal4;UAS:GCaMP6s)* (Fig. 4A). First, we asked how DRN axonal pixels respond to locomotor activity across multiple optical planes (Fig. 4B). We observed that a prominent fraction of DRN axons in the forebrain were significantly excited or inhibited by locomotion (Fig. 4C-E), consistent with DRN neuron activity (Fig. 2E-G). To quantify the functional topography of these DRN projections, we calculated pairwise correlations of axonal locomotor responses as a function of the distance between them within each forebrain hemisphere^67,86–88^. This analysis revealed significantly higher response correlations among nearby DRN axons, exceeding chance levels (Fig. 4F). To better visualize this functional topography, we spatially aligned forebrain recordings from all recorded fish and plotted the three-dimensional locations of excitatory (blue) and inhibitory (red) axonal responses to locomotion (Fig. 4G). Visualizing these axonal responses as two-dimensional histograms in the dorsal (Fig. 4H) and ventral (Fig. 4I) forebrain, together with saggital views in Fig. 4J, revealed that spatially distinct and only partially overlapping forebrain zones receive different locomotor-related information from the DRN. Visual and vibration-evoked activity followed similar principles but involved much smaller fractions of sensory-modulated DRN axons (Fig. 4K-Z). Notably, pairwise differences between the DRN axon responses to different stimuli revealed that forebrain DRN axons with diverse functional properties are spatially organized (Fig. S6). Our results suggest that the DRN communicates different types of information to distinct forebrain targets.

### Activity of the dorsal raphe projections and forebrain neuronal activity covary

**Fig. 5:**
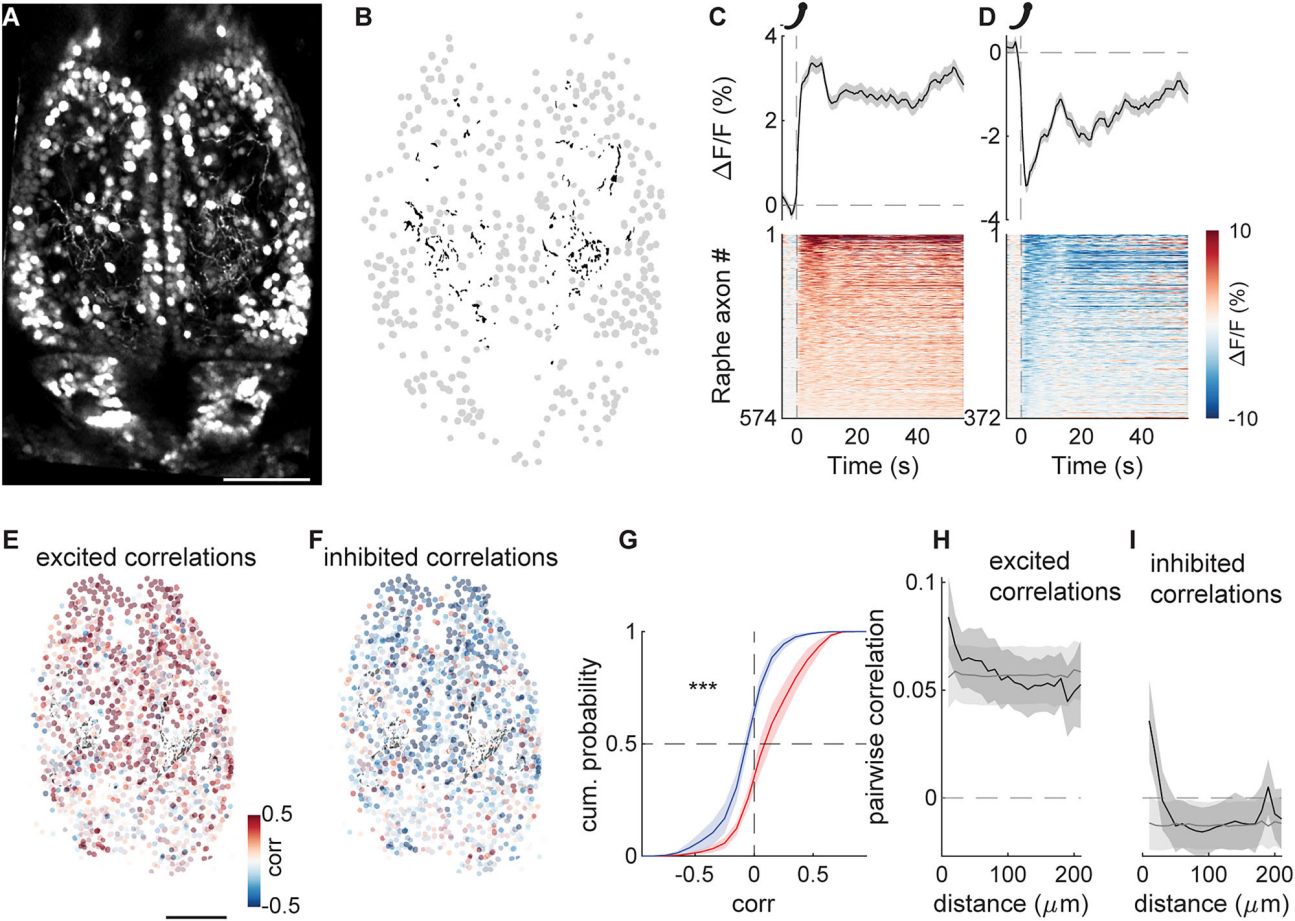
Locomotion related activity of dorsal raphe axons covaries with forebrain neurons. **A** Two-photon microscopy image of *Tg(HuC:Gcamp6s-nuclear)* labelled forebrain neurons together with *Tg(tph2:Gal4;UAS:GCaMP6s)* labelled dorsal raphe axons innervating juvenile zebrafish forebrain. **B** Two-dimensional reconstruction of dorsal raphe axons (black), and forebrain neurons (grey) identified in the image shown in panel **A**. **C**, **D** Tail-bout responses of excited (warm color) and inhbited (cold color) dorsal raphe axons in the forebrain. Horizontal dashed lines: zero-signal lines. Pearson’s correlations of activity between forebrain neurons and tail-bout excited (**E**) and inhibited (**F**) dorsal raphe axons. Black lines mark the dorsal raphe axons. **G** Cumulative distribution for correlations between forebrain neurons and excited (red), and inhibited (blue) dorsal raphe axons. Horizontal dashed line: 50%-probability line. Vertical dashed line: zero-correlation line. ***p = 0.0005, using linear mixed-effects model. Pairwise Pearson’s correlation between forebrain neurons and tail-bout excited (**H**) and inhibited (**I**) dorsal raphe axonal region of interests within each hemisphere as a function of their distance (*μm*) between them. Gray line represents shuffled spatial distribution. Horizontal dashed lines: zero-correlation lines. Responses are calculated within 10 s after stimulus onset represented by dashed vertical lines. *n* = 9 fish. Scale bars represent 100 μm. Line represents mean, shading represents SEM.

We observed that DRN projections in the forebrain respond to sensory-motor stimulation with topographically organized excitation and inhibition (Fig. 4). But how does this DRN axon activity relate to the activity of forebrain neurons? To address this, we volumetrically imaged the activity of DRN axons together with forebrain neurons. A triple transgenic zebrafish line, *Tg(tph2:Gal4; UAS:GCaMP6s; HuC:GCaMP6s-nuclear)*, enabled us to detect individual forebrain neuron nuclei independently from DRN axons in the forebrain, based on structural differences between axons and neuronal nuclei (Fig. 5A-B; see Methods). Consistent with our results in Fig. 4, we observed that spontaneous locomotion elicited excitation and inhibition in different populations of DRN axons (Fig. 5C-D) and across forebrain neurons (Fig. S7A-B). Next, we asked whether the activity of DRN axons in the forebrain covaries with the activity of surrounding forebrain neurons during locomotion. We observed that excited DRN axons show significantly higher positive correlations with forebrain neurons in comparison to inhibited DRN axons (Fig. 5E-G). Correlations of the excited DRN axons with the excited forebrain neurons were also significantly higher than the correlations of the inhibited axons (Fig. S7A,E), and vice versa for the correlations with the inhibited forebrain neurons (Fig. S7B,F).To quantify the spatial organization of these functional interactions, we calculated the correlations between the activity of DRN axons and forebrain neurons within the same hemisphere as a function of the distance between them. We observed that DRN axons exhibited stronger positive correlations with nearby forebrain neurons (Fig. 5H-I). Excited DRN axons on avergae were not closer to excited forebrain neurons than inhbited DRN axons (Fig. S7C). Excited axons were more correlated to excited forebrain neurons (Fig. S7E). This was similar between inhbited DRN axons and forebrain neurons (Fig. S7B-D-F). We observed similar yet weaker trends of correlations between DRN axons and forebrain neurons during ongoing activity (Fig. S7G-J) and for vibration-evoked responses (Fig. S7K-O). Despite the limitation that a small amount of DRN axons might spatially overlap with forebrain neuron nuclei, our results indicate that activity of DRN axons and forebrain neurons covary during locomotion, vibration responses and ongoing activity.

### Chemogenetic ablation of the dorsal raphe disrupts the synchrony and sensory-motor responses of forebrain neurons

**Fig. 6:**
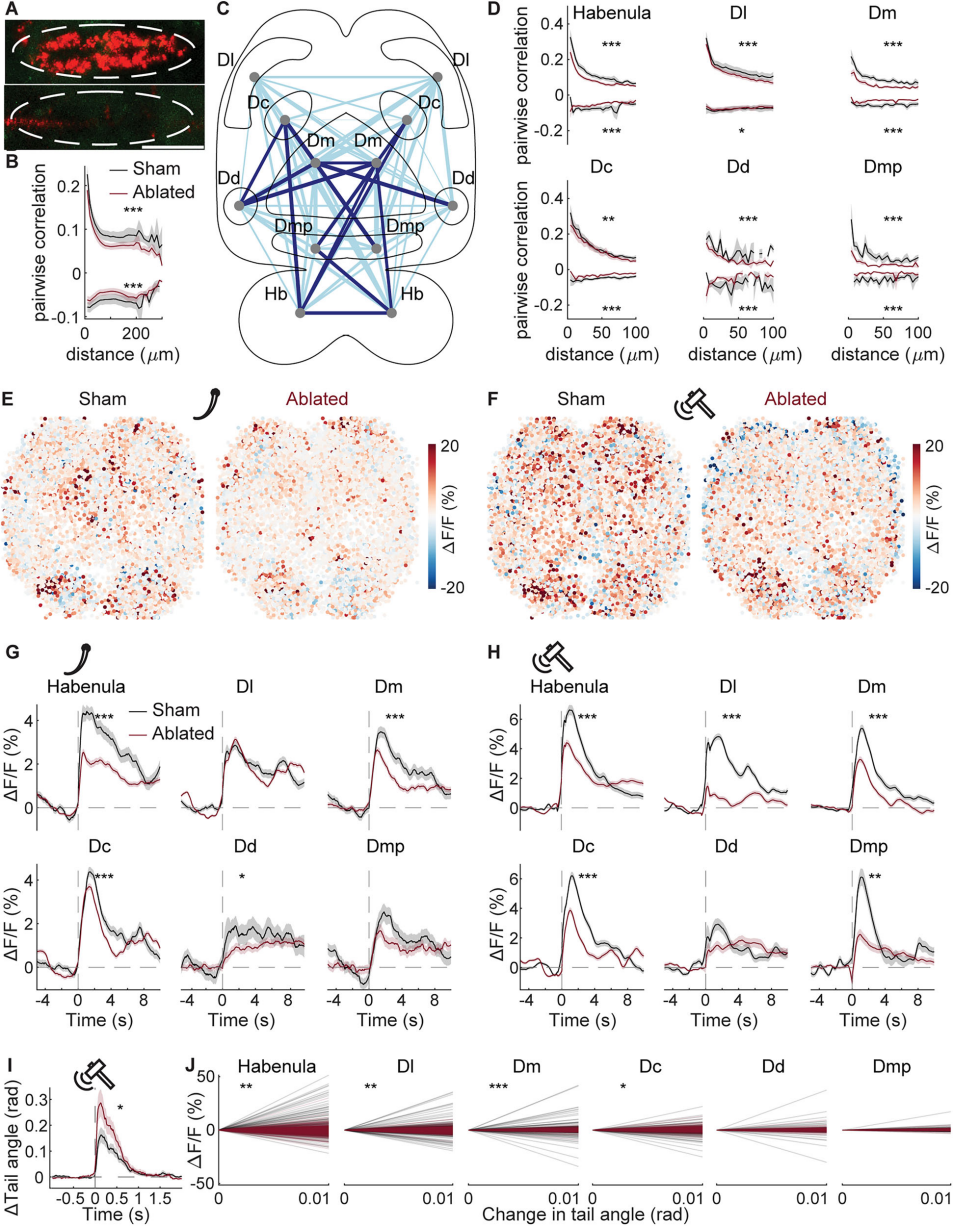
Dorsal raphe ablation impairs forebrain synchrony and sensory-motor responses. **A** Confocal microscopy of dorsal raphe in *Tg(tph2:Gal4;UAS:NTR-mCherry;HuC:GCamp6s)* juvenile zebrafish after sham (DMSO, top) or Metronidazole (DMSO + MTZ, bottom) treatment. Scale bar 50 μm. **B** Pairwise positive and negative Pearson’s correlations of forebrain neurons as a function of their distance (*μm*) between, in sham (black) and dorsal raphe ablated (brown) head-restrained juvenile zebrafish. Positive and negative correlations are significantly reduced. ANOVA: ***p = 8.4 × 10^-13^ (positive), ***p = 1.5 × 10^-7^ (negative). **C** Schematic illustration of alterations in forebrain functional connectivity upon chemogenetic dorsal raphe ablation, during vibration-evoked activity. The thickness of lines represents the average difference in correlations. Cyan represent a decrease, and blue represent a significant decrease. Abbreviated forebrain regions: Hb: Habenula, Dc: Dorsal-central, Dd: Dorsal-dorsal, Dl: Dorsal-lateral, Dm: Dorsal-medial, Dmp: Dorsal-medial-posterior, telencephalon. **D** Pairwise positive and negative Pearson’s correlations of neurons within identified forebrain regions as a function of their distance between, in sham (black) and dorsal raphe ablated (brown) zebrafish. ANOVA: Hb: ***p = 3.4 × 10^-23^ (positive), ***p = 4.0 × 10^-12^ (negative); Dl: ***p = 3.6 × 10^-14^ (positive), *p = 0.0243 (negative); Dm: ***p = 9.9 × 10^-32^ (positive), ***p = 8.8 × 10^-21^ (negative); Dc: **p = 0.0031 (positive), ***p = 9.5 × 10^-17^ (negative); Dd: ***p = 1.9 × 10^-7^ (positive), ***p = 5.2 × 10^-5^ (negative); Dmp: ***p = 1.6 × 10^-20^ (positive), ***p = 6.5 × 10^-18^ (negative). Spatial distribution of tail-bout (**E**) and vibration (**F**) responses (ΔF/F) in dorsal forebrain neurons in sham (left) and dorsal raphe ablated (right) animals. Warm colors excitation, cold colors inhibition. All fish are spatially aligned and overlaid. Average tail-bout (**G**) and vibration (**H**) responses of forebrain neurons in sham (black) and dorsal raphe ablated (brown) zebrafish. Neural responses (5 s after stimulus) in dorsal raphe ablated zebrafish are reduced: In G, Hb: ***p = 2.9 × 10^-10^; Dm: ***p = 3.7 × 10^-6^; Dc: ***p = 0.0001; Dd: *p = 0.0231, in H, Hb: ***p = 1.4 × 10^-6^; Dl: ***p = 0; Dm: ***p = 1.2 × 10^-10^; Dc: ***p = 4.9 × 10^-14^; Dmp: **p = 0.0012, using linear mixed-effects model. **I** Average behavioral responses to vibrations for sham (black), and ablated (brown) zebrafish (same fish as in panel **E**–**H**) *p = 0.0483, using linear mixed-effects model. Locomotion amplitude are calculated within 1 s after stimulus onset represented by the dashed vertical line. Horizontal dashed-line marks zero. **J** Slopes of linear fits per indvidual neuronal activity change in tail angle, upon vibrations sham (black), and ablated (brown). Relation between neural and behavioral responses in dorsal raphe ablated zebrafish is reduced. Hb: **p = 0.0097; Dl: **p = 0.0051; Dm: ***p = 1.0×10^-5^; Dc: *p = 0.0489, two-sided Wilcoxon ranksum test. Dorsal raphe ablated: *n* = 16 fish and 9709 neurons; sham: *n* = 16 fish and 8321 neurons. # neurons in sham: Hb = 1698, Dl = 2590, Dm = 1241, Dc = 1575, Dd = 399, Dmp = 372; # neurons in ablated: Hb = 2158, Dl = 3121, Dm = 1278, Dc = 1795, Dd = 533, Dmp = 344. Line represents mean, shading represents SEM.

Subsequently, we asked what role DRN projections play in regulating forebrain activity. To address this, we used a triple transgenic zebrafish expressing nitroreductase (NTR)^62,92–94^ for chemogenetic ablation of the DRN, while enabling panneuronal activity measurements, *Tg(tph2:Gal4; UAS-E1b:NTR-mCherry; HuC:GCaMP6s)*. A 24-hour treatment with 10 mM metronidazole (MTZ)^62,94^ resulted in complete DRN ablation (Fig. 6A). Given the broad forebrain innervation of DRN axons, we first asked whether DRN ablation impairs the orchestration of neurons in the dorsal zebrafish forebrain, which is proposed to contain homologs of the mammalian cortico-limbic systems^70,73,75,76,95–105^ and the habenula^93,106,107^. We quantified neural synchrony across the dorsal forebrain by calculating pairwise positive and negative correlations between neurons. We observed that both positive and negative pairwise correlations were stronger between nearby neurons and weaker between distant dorsal forebrain neurons, highlighting prominent functional topography during vibration stimulation period (Fig. 6B, black traces). Upon DRN ablation, both positive and negative correlations between dorsal forebrain neurons exhibit a small but significant reduction (Fig. 6B, red traces). To test the impact of DRN ablation on the functional interactions between dorsal forebrain regions, we calculated pairwise correlations of neurons located in individual regions^108^ identified using anatomical landmarks^60^. We observed an overall reduction in correlations between regions (Fig. 6C, cyan lines) and significant reductions in correlations between the central and posterior regions (Dm, Dc, Dmp, Dd, Hb) of the dorsal forebrain (Fig. 6C, blue lines). In DRN ablated animals, neurons within individual forebrain regions also exhibited weaker positive and negative correlations that were spatially organized (Fig. 6D). Impact of DRN ablation on forebrain synchrony remained similar when forebrain neurons with matching levels of vibration responses were compared as additional control (Fig. S8A-D). Finally, we observed smaller but significant reductions of forebrain synchrony during ongoing activity (Fig. S8E–G). All together these results revealed a reduction in forebrain synchrony after DRN ablation.

To further investigate the impact of the DRN on forebrain activity, we examined how forebrain responses during locomotion and aversive mechanical vibrations^67,87^ are altered by DRN perturbation. To visualize this, we spatially aligned all recorded neurons from all fish (sham: *n* = 16 fish and 8321 neurons, ablated: *n* = 16 and 9709 neurons) and plotted their average responses during locomotor bouts (Fig. 6E) and vibration stimuli (Fig. 6F). In DRN ablated animals, we observed weaker responses across dorsal forebrain. To determine whether responses in individual forebrain regions were differentially altered by DRN ablation, we quantified and compared response amplitudes from thousands of individual forebrain neurons. Locomotor responses were significantly weaker in central and posterior dorsal regions such as Dm, Dc, Dd, and the habenula (Fig. 6G). Vibration-evoked responses were significantly weaker in Dl, Dm, Dc, Dmp, and the habenula (Fig. 6H).

Our head-restrained juvenile zebrafish preparation enabled measurement of the animals’ locomotor behavior in response to aversive mechanical vibrations^67,87^. Comparing the DRN-ablated group to the sham group revealed a significant increase in the amplitude of locomotor bouts upon mechanical vibrations (Fig. 6I), while spontaneous locomotor activity during the baseline period was not changed (Fig. S8H). Finally, we asked whether DRN ablation altered the relationship between neural and behavioral responses to vibrations. To address this, we quantified trial-by-trial relationship between forebrain activity at the single-neuron level and changes in locomotor behavior. For each neuron, we computed a linear fit of neural response versus animals’ behavioral response and compared the distribution of slopes between sham and DRN ablated groups. DRN ablation resulted in a small but significant alteration in the relationship between behavioral and neural responses for the habenula, Dl, Dm, and Dc (Fig. 6J). Taken together, our results highlight the importance of DRN in regulating forebrain activity.

### Dorsal raphe ablation impairs defensive behaviors in juvenile zebrafish

It is essential for animals to initiate defensive behaviours upon threats and recover after the threat to explore their surroundings for potential resources. Such defensive behaviours also requires assessment of the intensity and history of threats, as well as to analyze associated risks and benefits. Juvenile and adult zebrafish are known to exhibit lasting bottom-diving behavior in response to immediate threats^109^, followed by a gradual recovery to their baseline exploratory state^67,110–112^. We investigated the impact of DRN ablation on two such defensive behaviors.

**Fig. 7:**
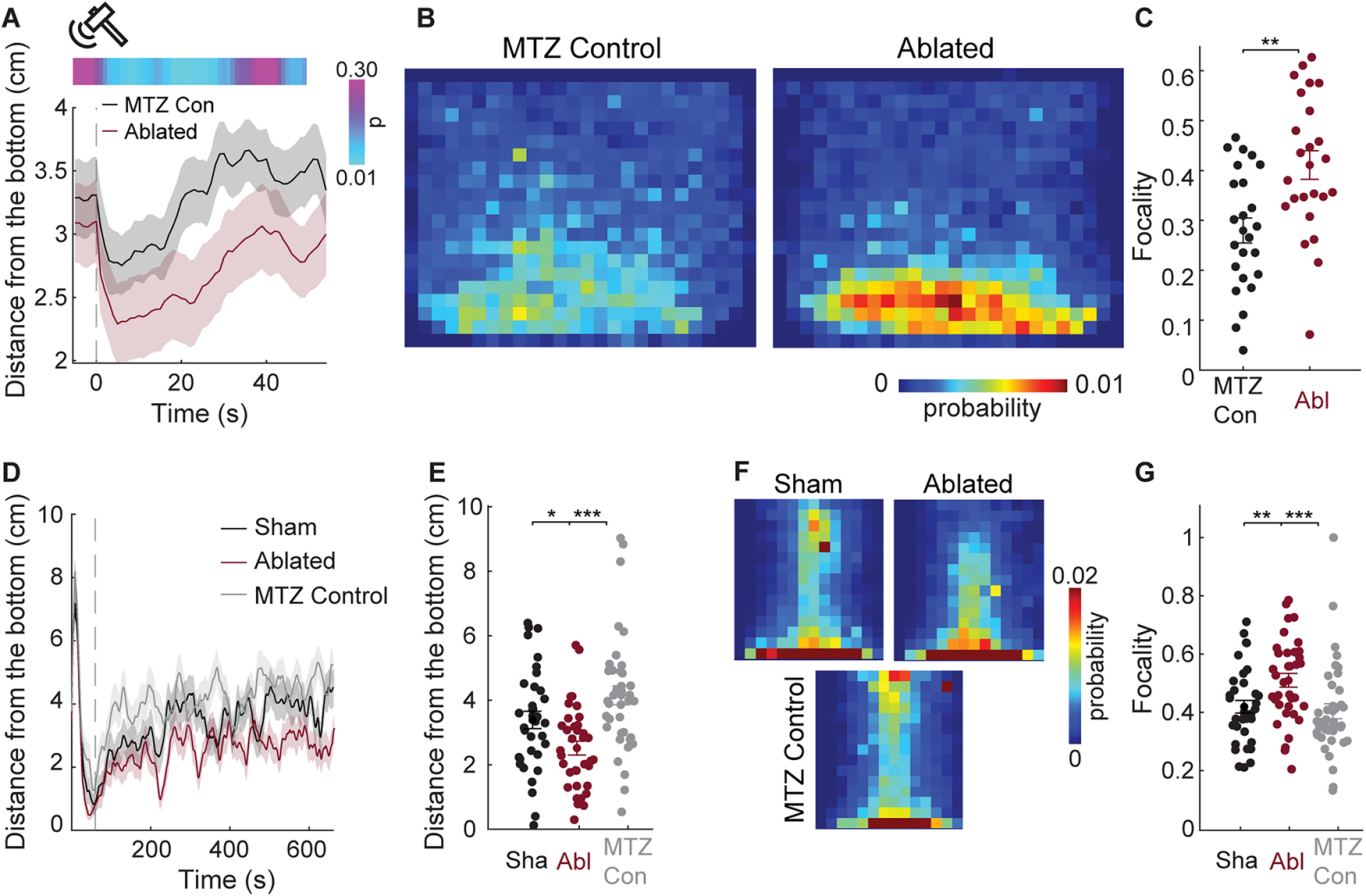
Dorsal raphe ablation impairs adaptive behaviors in freely swimming zebrafish. **A** Average time course of vertical swim position (distance from the bottom) in response to mechanical vibrations in MTZ-control (black, *n* = 25), and dorsal raphe ablated (brown, *n* = 24) juvenile zebrafish. Statistical differences were plotted as a function of time, where *p*-values are calculated across consecutive 5 s windows (two-sided Wilcoxon ranksum test). Note that dorsal raphe ablated zebrafish swim closer to the bottom of the tank for up to 20-30 seconds after transient vibration stimuli. Vertical line: stimulus onset. **B** Heatmaps representing the average position probability of freely swimming zebrafish during mechanical vibration response for the same groups as in panels **A**, **B**. **C** Focality of the position probability in panel C during mechanical vibration response. Note that dorsal raphe ablated zebrafish (brown) swim significantly more focal (less exploration), in comparison to MTZ-control (black) zebrafish. **p = 0.0020, two-sided Wilcoxon ranksum test. **D** Average time course of vertical swim position (distance from the bottom) during novel tank response in sham (black, *n* = 35), MTZ-control (grey, *n* = 39), and dorsal raphe ablated (brown, *n* = 36) juvenile zebrafish. Vertical line: initial diving (60 s). Response window: 600 s. **E** Average distance from the bottom for individual zebrafish during novel tank response. *p = 0.0134, ***p = 2.2 × 10^−5^, two-sided Wilcoxon ranksum test. Note that dorsal raphe ablated zebrafish (brown) swim significantly closer to the bottom, in comparison to sham (black) and MTZ-control (grey) zebrafish. **F** Heatmaps representing the average position probability of freely swimming zebrafish during the novel tank response for the same three groups as in panels **E**, **F**. **G** Focality of the position probability in panel G during the novel tank response. Note that dorsal raphe ablated zebrafish (brown) swim significantly more focal (less exploration), in comparison to sham (black) and MTZ-control (grey) zebrafish. **p = 0.0072, ***p = 0.0004, two-sided Wilcoxon ranksum test. Line represents mean, shading represents SEM. Error bar represents mean ± SEM.

To do this, we quantified juvenile zebrafish behavior from a vertical view and observed that upon mechanical vibrations, juvenile zebrafish perform an immediate bottom dive, followed by a slow and gradual climb upward^67^ (Fig. 7A). Up on mechanical vibration, DRN-ablated zebrafish swim closer the bottom of the tank (Fig. 7A). Swim depth preference before vibration stimuli was not affected by DRN ablation (see *p*-values before dashed line in Fig. 7A). Recovery after reaching the deepest dive point was not different between the two groups (Fig. S9A-B). DRN-ablated zebrafish exhibited a denser occupancy probability closer to the bottom of the tank, suggesting reduced exploration of the tank, for up to 20 seconds after mechanical vibrations (Fig. 7B). To quantify exploration, we used a focality measure, where a focality value of “1” indicates no exploration and “0” indicates complete exploration of all possible tank locations (see Methods for description). DRN-ablated zebrafish exhibited significantly higher focality, indicating reduced exploration (Fig. 7C).

When introduced to a novel tank, juvenile zebrafish display a prominent defensive behavior, performing a deep bottom dive followed by a gradual period of upward climbing^113,114^ (Fig. 7D). During this period, DRN-ablated zebrafish exhibited significantly lower y-positions, remaining closer to the bottom of the tank compared to controls (Fig. 7D-E). Furthermore, DRN-ablated zebrafish demonstrated a denser occupancy probability near the bottom of the tank, with significantly higher focality values indicating reduced exploration (Fig. 7F,G). Baseline swimming speed was unaffected in DRN ablated animals (Fig. S9C). Together, these findings revealed that DRN ablated zebrafish exhibit amplified defensive behaviours and reduced exploration after experiencing an environmental threat.

## Discussion

### Diversity of DRN neurons and their axonal projections in the forebrain

In this study, we observed a remarkable diversity of functional features across zebrafish DRN neurons and their axonal projections in the forebrain. Mammalian DRN neurons have been shown to be diverse, with molecularly distinct groups of neurons located in topographically organized DRN zones^36,54,115^. Here, we revealed that the zebrafish DRN is composed of topographically organized functional ensembles, where nearby DRN neurons exhibit similar ongoing, sensory and locomotor evoked activity. In vivo population recordings of individual rodent DRN neurons have recently revealed functionally distinct neural ensembles^116^. This study also suggests spatial segregation of neural ensembles recorded in a small fraction of the DRN. These findings are in line with our recordings covering the entire zebrafish DRN. Furthermore, we observed that a *Gad1b*-expressing population of DRN neurons exhibit functionally distinct neural activity in juvenile zebrafish. Interestingly, younger zebrafish larvae do not have such *Gad1*-positive DRN neurons^19,117^, suggesting a developmental increase in the molecular diversity of DRN neurons. Yet, *Gad1* co-expressing neurons are present in the mice DRN^35^, at a dorsal location similar to juvenile zebrafish DRN. These results indicate a conservation of developmental principles underlying the molecular topography of the DRN. Future studies on evolutionary comparison of molecularly defined DRN cell types, together with their development and topography, are needed for a better understanding of DRN function across species.

In line with the functional diversity of DRN neurons, we also observed that DRN axons projecting to the zebrafish forebrain exhibit topographically organized activity. To our knowledge, functional measurements of DRN axons activity across the mammalian forebrain have not been done. Nevertheless, anatomical viral tracing experiments revealed that molecularly distinct groups of mice DRN neurons innervate distinct cortical and subcortical regions^35,54^, suggesting a specialization of DRN information received by distinct forebrain targets. The same study revealed that while *Vglut3*-expressing DRN neurons primarily target cortical regions, *Trh/Gad1/Gad2*-expressing DRN neurons target subcortical regions. We observed that locomotion-inhibited DRN axons are primarily present at a central region extending from the ventral (preoptic area of the hypothalamus, pallidum) to the dorsal (amygdala homolog Dm) zebrafish telencephalon (Fig. 4G-H, in blue). Moreover, a stronger preference for locomotion-evoked inhibition in *Gad1b*-expressing and anterior-dorsal DRN neurons (Figs. 2H, 3J-K and Supplementary Fig. 4D) suggests that locomotion-inhibited DRN axons may, at least in part, originate from a distinct DRN population. Indeed, *Trh/Gad1* co-expressing DRN neurons in mice were shown to project to distinct hypothalamic, amygdalar, and pallidal regions^35,54^, supporting the functional specialization of forebrain-projecting DRN axons that we have observed in zebrafish. We also observed that locomotion-excited DRN axons from primarily *Gad1b*-negative and posterior-ventral DRN neurons (Figs. 2H,  3 and Supplementary Fig. 4C) innervate the zebrafish olfactory bulb and piriform cortex homolog Dp, as well as anterior, central, and lateral regions of the dorsal telencephalon (Fig. 4G-H), which are proposed to be homologous to mammalian hippocampus and cortical structures^70,73,75,76,95–105^, resembling axonal innervation patterns of *Vglut3*-expressing DRN neurons in mice^35,54^. Altogether, our findings provide important evidence for differential functional features of DRN axons innervating topographically distinct forebrain regions.

### What do DRN axons communicate to the forebrain?

Recent studies provide detailed expression maps of diverse inhibitory and excitatory serotonin receptors across the mammalian^118,119^ and larval zebrafish brain^26^. These findings support the idea that DRN inputs are differentially interpreted by recipient neurons depending on their serotonin receptor profiles. Moreover, DRN neurons were shown to co-release serotonin together with GABA^120^ or glutamate^121^, which means DRN axons can interact with their targets through multiple mechanisms. We observed that DRN axons’ activity exhibits covariance with forebrain neurons (Fig. 5H-I). We acknowledge that such covariance might be affected by a small amount of DRN axons spatially overlapping with forebrain neuron nuclei, potentially limiting the interpretation of these correlations. Nevertheless, a recent study showed that optogenetic activation of DRN neurons leads to a rapid increase in ongoing bursting activity in the zebrafish dorsal telencephalon^85^. These results are in line with our findings that DRN axons are correlated with forebrain neuron activity, and that genetic ablation of DRN leads to an overall reduction in sensory and motor responses and decorrelation of activity across the entire zebrafish dorsal telencephalon (Fig. 6). Hence, the net effect of DRN inputs to the dorsal telencephalon appears to be facilitating and orchestrating. While the reduction in forebrain correlations following DRN ablation (Fig. 6D) is statistically significant, we acknowledge that the absolute differences are modest. This is consistent with the nature of neuromodulatory systems, which typically exert subtle but widespread influence rather than producing binary effects. Importantly, we do not expect forebrain synchrony to be entirely abolished, as locomotor-related information can reach the forebrain through multiple parallel pathways, including direct sensory inputs and other neuromodulatory systems.

Traditionally, DRN activity is associated with the encoding of emotions, mood^4,39,41–43^, appetite^44,45^, sleep^20,46^, or even socialization^37,122^. Zebrafish DRN was shown to encode alterations of ambient light levels^39^. Intriguingly, our results revealed that locomotion and mechanical vibrations are prominent drivers of excitation and inhibition in distinct populations of zebrafish DRN neurons. This means that DRN has access to information related to rapid environmental changes and animals’ motor activity. In fact, we observed that chemogenetic DRN ablation led to a decoupling between zebrafish forebrain activity and locomotion (Fig. 6J). Hence, our results suggest that DRN might be an additional pathway, in parallel to other channels^14,123–127^, communicating information about animal locomotion to the forebrain. Sensory-motor information was shown to be available also to DRN^128^ and DRN axons^129^ innervating the mammalian cortex, suggesting that not only the anatomy^16^, but also the function of DRN axons in the forebrain might be conserved across the vertebrates.

### The role of DRN in regulating brain and behavior

DRN was shown to integrate motor, arousal, and stress-related signals to shape brain state in zebrafish and mammals^19,40,116,128,130,131^. Moreover serotonergic plasticity, including psilocybin induced behavioral changes, demonstrates conserved neuromodulatory mechanisms that reconfigure network activity across species^117,132,133^. We propose that locomotor signals from DRN can be utilized to modulate forebrain activity and amplify forebrain sensory responses. While we also observed that broad DRN ablation amplifies defensive behaviours, the specific role of DRN to forebrain projections in defensive behaviours is yet to be determined. For example, DRN neurons project broadly throughout the brain^35,90,91^, and we did not dissect the individual contributions of these different DRN projections, which is a limitation of our study. Future work will be necessary to tease apart the influences of different DRN subpopulations and their projections on brain computations and animal behaviour.

Two recent brain-wide imaging studies in larval zebrafish identified DRN as the primary region associated with the switch between animals’ exploration and exploitation states^134^, as well as the switch between active to passive coping states^135^. Similarly, DRN was also shown to be important for motor adaptation in zebrafish larvae, during which animals have to track the outcome of their actions and adjust the gain of their locomotory activity^38^. Given all these and our results, it is possible that the well-established role of DRN in mood and internal states^4,20,39,41–46^ might have evolved from humbler evolutionary beginnings, in which DRN-forebrain interactions mediate the communication between animals’ locomotor activity and sensory information processing. We therefore propose that future studies should focus not only on the role of DRN in encoding and regulating complex high-level behaviors but also on how simpler sensory-motor actions can influence such computations.

## Methods

The key resources utilized in this study are listed in Table 1.

**Table 1:** Key resources for this study

Reagent or Resource	Source	Identifier
**Chemicals**		
MS222 (Tricaine methanesulfonate)	Sigma-Aldrich	Cat# E10521
LMP Agarose	Fisher Scientific	Cat# 16520100
Metronidazole (MTZ)	Sigma-Aldrich	Cat# M1547
**Zebrafish lines with brief description)**	**Source and description**	
*Tg(tph2:Gal4;UAS:caax-GFP)*	Yokogawa et al. 2012^40^	N/A
*Tg(tph2:Gal4;UAS:GCaMP6s)*	Yokogawa et al. 2012^40^, Diaz et al. 2019^59^	N/A
*Tg(Gad1:dsRed//tph2:Gal4;UAS:GCamp6s)*	Yokogawa et al. 2012^40^, Diaz et al. 2019^59^, Satou et al. 2013^89^	N/A
*Tg(tph2:Gal4;UAS:Gcamp6s//HuC:Gcamp6s nuclear)*	Yokogawa et al. 2012^40^, Diaz et al. 2019^59^	N/A
*Tg(tph2:Gal4;UAS:NTR-mCherry//HuC:GCamp6s)*	Yokogawa et al. 2012^40^, Diaz et al. 2019^59^, Palumbo et al.^62^	N/A
*Nacre (mitfa*^*-/-*^*)*^*b692*^	^148^	ZFIN:ZDB-ALT-010919-2
**Software and Algorithms**		
ImageJ/Fiji	https://fiji.sc/	N/A
Cell detection, Image alignment and processing	^59,86,137,139^	N/A
**Other**		
Two-photon microscope	Scientifica	N/A

## Experimental model and subject details

### Fish husbandry

NFSA (Norwegian Food Safety Authority) has approved the animal facility and fish maintenance. Zebrafish, *Danio rerio*, were kept in 3,5 L tanks in a Tecniplast ZebTec Multilinking System. Constant conditions were maintained: 28.5 °C, pH 7.2, 700μSiemens. 14:10 hour light/dark cycle was preserved. Dry food (SDS100 up to 14dpf and SDS 400 for adult animals, Tecnilab BMI, the Netherlands) was given to fish twice a day, in addition to Artemia nauplii (Grade 0, Platinum Label, Argent Laboratories, Redmond, USA) once a day. From fertilization to 3dpf (days post fertilization) larvae were kept in a Petri dish with egg water (1.2 g marine salt in 20 L RO water, 1:1000 0.1% methylene blue) and between 3 and 5dpf in artificial fish water (AFW: 1.2 g marine salt in 20 L RO water). Juvenile (3 to 4-week-old) zebrafish were used for the experiments.

*Tg(tph2:Gal4;UAS:GCaMP6s), Tg(tph2:Gal4;UAS:GCamp6s;Gad1:dsRed), Tg(tph2:Gal4;UAS:Gcamp6s//HuC:Gcamp6s-nuclear*), and *Tg(tph2:Gal4;UAS:NTR-mCherry;HuC:GCamp6s)* zebrafish lines were used for calcium imaging.

### Metronidazole treatment

To ablate DRN neurons, *Tg(tph2:Gal4;UAS:NTR-mCherry;HuC:GCamp6s*, for calcium imaging experiments) or *Tg(tph2:Gal4;UAS:NTR-mCherry*, for freely-swimming experiments) three-weeks (21 days) old juvenile zebrafish were used. 2 days before the experiment (at 19 dpf), the fish were treated with 10 mM metronidazole (MTZ) (Sigma-Aldrich) and 0.5% DMSO in AFW for 24 h (from 19 to 20 dpf). Three fish were placed in a Petri dish containing 50 mL of 10 mM MTZ & 0.5% DMSO in AFW in a 28 °C incubator, with regular light/dark cycles (14 h light/10 h dark). Following the treatment, the fish were transferred to a new Petri dish containing fresh AFW. Animals were fed with dry food and placed back in the incubator for the washout period of at least 12 h (from 20 to 21 dpf).

Sham controls were siblings of the DRN ablation group identically except for the absence of metronidazole. Specifically, sham fish were exposed to the same handling, embedding, imaging, and drug treatment procedures^62,94^, but received only dimethyl sulfoxide (0,5% DMSO). For novel tank diving test we also added an additional control group (MTZ-Control), in order to test if 10 mM MTZ treatment might have any non-specific side effects on animals that lack the nitroreductase (NTR) transgene. We observed no differences between our sham controls and MTZ-controls in none of the parameters measured for novel tank diving test. Both sham-controls and MTZ-control significantly differed from DRN ablated group. Hence we concluded minimal non-specific impact of 10 mM MTZ treatment at the concentration we have used in juvenile zebrafish. All other experimental conditions (age, size, imaging setup, behavioral assays) were matched across groups.

### Inclusion and ethics statement

Our team includes researchers from diverse origins and backgrounds. One or more of the authors of this paper self-identifies as an underrepresented minority in science.

All experimental procedures performed on zebrafish larvae and juveniles were in accordance with the Directive 2010/63/EU of the European Parliament and the Council of the European Union and approved by the Norwegian Food Safety Authorities. Animals from both sexes were used in this study.

## Method details

### Two-photon calcium imaging and sensory stimulation in head-restrained juvenile zebrafish

For in vivo imaging, 21 dpf juvenile fish were embedded in 2.5% low-melting-point agarose (LMP, Fisher Scientific) in the lid of a 35 mm Petri dish (FALCON). The constant perfusion of AFW buffered with 10 mM NaHCO3 and bubbled with carbogen (95% O2 and 5% CO2) at 27 °C was maintained during the experiment. After solidifying for 20 minutes, LMP agarose was carefully removed in front of the mouth and posterior to the swim bladder.

A two-photon microscope (Scientifica Inc.) with a 16x water immersion objective (Nikon, NA 0.8, LWD 3.0) was used for calcium imaging. For excitation, a mode-locked Ti:Sapphire laser (MaiTai Spectra-Physics) was tuned to 920 nm. Recordings were performed as either single-plane or volumetric recordings (8 planes with a Piezo (Physik Instrumente (PI))). The acquisition rate was 18.61 Hz for single-plane recordings (image size 1536 × 850 pixels) and 2.33 Hz per plane for volumetric scans (image size 1536 × 850 pixels).

First, ongoing spontanous activity was measured for 3 min. Afterwards, six repetitions of each sensory stimuli (red light flash or vibrations) were applied. All our sensory stimulation parameters are selected based on our earlier studies^67,87,136^. For the light stimulus, we used a red LED (LZ1-00R105, LedEngin; 625-nm wavelength) placed in front of the recording chamber near the tube. The light stimulus was a flash of 200 ms duration with an intensity of 0.318 mW, measured at the position of the fish. Vibrations were delivered via a solenoid tapper (SparkFun Electronics, ROB-10391) with a 200 ms application of 12 V. Please see vibration stimulus intensity/frequency parameters in Supplementary Fig. 3A. To reduce potential adaptation, we introduced 1 min interstimulus interval for each stimulus delivery.The total duration of the recordings was 30 minutes.

### Imaging of locomotion in head-restrained zebrafish

For simultaneous imaging of calcium signals and locomotor activity, a Manta camera (G-031, Allied Vision; recording at 120 fps) was placed underneath the juvenile zebrafish. A custom-built IR light source (780 nm LEDs) was placed around the microscope objective above the fish to provide maximal IR illumination.

### Temporal synchronization of neural activity, stimulus delivery and locomotion recordings

All functional imaging experiments were performed volumetrically, except for the experiments in Fig. 6, which used single-plane imaging. Volumes were acquired using two-photon microscopy with a piezo-driven objective, scanning sequential planes from dorsal to ventral in a fixed order for every volume. Each volume consisted of 8 optical planes (1536 × 850 pixels), with an acquisition rate of ~2.3 Hz per volume ( ~ 18.4 Hz per frame for single plane experiments).

Stimulus triggers (light flashes or vibrations) were controlled by Arduino connected to the microscope and computer enabling stimulus delivery at specific two-photon frame numbers. To capture additional reference synchronization timing markers in the behavioral video recordings, we used Arduino generated synchronization pulses turning off the LEDs at specific two-photon microsocpy frames in the beginning, and end of the recording session, producing two dark frame blocks in the video. During offline preprocessing, the two-photon microscopy frame-triggered synchronous light-off-pulses were utilized to ensure that there is no synchronization problems between two-photon microscopy recordings, stimulus delivery and locomotion recordings.

### Measuring behaviors in freely-swimming zebrafish

For tracking behaviors in freely-swimming 21 dpf juvenile zebrafish, we used Zantiks LT set up with 6 tanks (10 × 11.5 × 3 cm) enabling experiments with six fish simultaneously while recording their vertical and horizontal positions in tanks. Ambient white light was available during all behavioral experiment. During novel tank diving experiment, the animals were gently placed into their respective tanks filled with AFW. Their movement during the experimental procedure was tracked at 15 Hz, in dimensions viewed from the side. Fish with tracking errors were identified manually and were not included in further analysis.

During vibration stimulation experiments 6 plastic arenas (11.5 × 11.5 × 1.5 cm) glued to an acrylic plate using epoxy resin were used. For analyzing the fish’s behavior, the fish were tracked at 10 Hz. To deliver the vibration stimuli, a “Solenoid-tapper”-device was coupled by a microcontroller Arduino Due to the arenas and programmed with a custom-software. After a 30 minute of adaptation period without any external stimuli, the tapper began to tap on the arenas (6 trials). All experiments were performed at room temperature around 24 °C.

## Quantification and statistical analysis

Two-photon microscopy images were aligned using a method described in^59,60,137^ (occasional XY drift was corrected, based on “hierarchical model-based motion estimation”^138^) or using suite2p^139^ (Video S1). Recordings were visually inspected for remaining motion and Z-drift, recordings with remaining motion artifacts were discarded. Regions of interest (ROIs) corresponding to neurons were automatically detected using a template matching algorithm^59,60,86^, and visually confirmed. To calculate the time course of each neuron, pixels belonging to each ROI were averaged over time. For each ROI, fractional change in fluorescence (ΔF/F) relative to baseline was calculated.

Functional clusters of neurons were calculated by using k-means clustering algorithm in MATLAB (Figs. 1D, and 3E)^86^. To identify optimal number of k-means clusters in the dorsal raphe, we used elbow method^140^. First, the distance of each cluster element to the centroid of that specific cluster was calculated, and this value was normalized by distances from each cluster element to every centroid. This operation is iterated for 1-30 number of cluster number ‘ks’ (Fig. S1A, black traces), and compared to shuffled/simulated data with same mean and variance (by shuffling the individual time series within each neuron), but no clustering (Fig. S1A, gray traces). To reveal the optimal clusters number, we subtracted values for real data from shuffled/simulated data (Fig. S1B). These analyses revealed that 3 minutes of ongoing DRN activity can be optimally represented by 4 clusters, which we choose to use in our analysis.

Cluster fidelity was calculated by measuring the probability of pairs of neurons being in the same cluster during two consecutive 1.5 min periods^86^. We compared the cluster fidelity of real k-means clusters with shuffled the cluster identities of same neurons.

Excitation or inhibition (upon sensory stimulation or locomotion) was determined by comparing the average activity during baseline (5 s before stimulus onset) with the average activity during response window. DRN neurons and axons exhibit slower calcium transients than forebrain neurons, hence we chose longer response windows for DRN recordings (10 s or 20 s), when compared to forebrain neurons (5 s) after stimulus onset). Significant responses were calculated using one-tailed Wilcoxon signed-rank test, across 5-6 repetition of each stimuli.

In Fig. 3, cluster selectivity was calculated to quantify the overlap of *Gad1b*-positive neurons with functional clusters of neurons identified using k-means clustering based on their ongoing activity. Cluster selectivity index is the life-time sparseness^86^ for the distribution of *Gad1b*-positive neurons across 4 k-means clusters. If cluster selectivity is 1, it means that *Gad1b*-positive neurons belong to one functional cluster. If cluster selectivity is 0, all *Gad1b*-positive neurons are equally distributed into all functional k-means clusters.

In Fig. 4, to identifty pixels corresponsing to DRN axons, we applied a pixel-intensity-based thresholding approach. Pixels with intensity values greater than 10% of the maximum pixel intensity in that plane were classified as axonal pixels.

In Fig. 5, to identify ROIs corresponsing to DRN axons, we first manually excluded forebrain regions with neuronal nuclei, expressing *HuC:Gcamp6s-nuclear*. Then, we adopted an independent component analysis (ICA) based method for axonal ROI detecion from Mukamel, E.A. et al. Neuron (2009)^141,142^. Once axonal ROIs are identified with ICA, we did visual inspection of ROIs and confirmed their overlap with DRN axons. The remaining analysis for this figure were done based on those DRN axonal ROIs, corresponding to DRN axonal segments. Later these DRN axonal ROIs were used for further analysis of calcium acitivity in individual axonal segments.

In Fig. 6, delineation of brain regions in the telencephalon was done manually on the raw image of the brain based on anatomical landmarks^143^ described in previous studies in zebrafish and other teleost fish^71,72,99,144,145^. The diagram in Fig. 6C was plotted in MATLAB and overlayed on the forebrain drawing prepared in Adobe Illustrator (same approach in Fig. S8C, F). In each animal, time course of the tail angle change (TAC) in each trial was calculated using a baseline period of 1 s before the stimulus onset. Response window for calculating average TAC was 1 s. The linear model for the relationship between neural and behavioral response is given by the equation:

1 $${y}_{i}={{{{\rm{\beta }}}}}_{0}+{{{{\rm{\beta }}}}}_{1}\cdot {x}_{i}+{{{{\rm{\varepsilon }}}}}_{i}$$

y: neural response (average ΔF/F in response window),

i: neuron index, β_0-1_: effects, x: behavioral response (average TAC in response window), ε: error term.

### Time windows for pairwise correlation calculations

We used different time windows for calculating pairwise correlations depending on the experimental context and available duration of the neural data. For analysis of ongoing activity (e.g. in Fig. 1), correlations were computed over the entire ongoing activity period (3 minutes for Fig. 1). For analysis of evoked activity (e.g. in Fig. 2), correlations were calculated using trial-averaged responses of individual neurons within 1-min trial window.

### Statistics

Statistical analysis was done using MATLAB; *p*-values are represented in the figure legends as (*p < 0.05, **p < 0.01, ***p < 0.001). A linear mixed-effects model with a restricted maximum likelihood^141,146,147^ was used for non-paired comparisons, and Wilcoxon signed rank test for paired comparisons. Linear mixed-effects models consider both the nested (observation within a single animal for a particular stimulus) and crossed (observation across animals) structure of the data. Thus, by treating animals as random effects, and ablation as a fixed effect, our results comparing for ablation can be generalized to the population of animals. All analysis was performed with Fiji and MATLAB.

### Reporting summary

Further information on research design is available in the Nature Portfolio Reporting Summary linked to this article.

## Supplementary information

Supplementary Information

Description of Additional Supplementary Files

Supplementary Video 1

Reporting Summary

Transparent Peer Review file

## Source data

Source data

## Data Availability

Calcium imaging data reported in this paper have been deposited in the sigma2 data base under the accession code 10.11582/2026.93nyzmjx. Source data are provided with this paper as a Source Data file.

## Code availability

Main associated codes that are used to make figures are available at: 10.5281/zenodo.19452784 and https://github.com/yaksilab/drn_paper.

## Materials availability

This study did not generate new unique reagents.
