# Supplementary material for: Topographically organized dorsal raphe activity modulates forebrain sensory-motor representations and contributes to defensive behaviors
Source: Nat Commun. 2026 Jul 16;17:6243. doi: 10.1038/s41467-026-75490-y (PMC13376501; doi:10.1038/s41467-026-75490-y)
Supplement: Supplementary file 1 — Supplementary Information [file 41467_2026_75490_MOESM1_ESM.pdf]

Supplemental figures

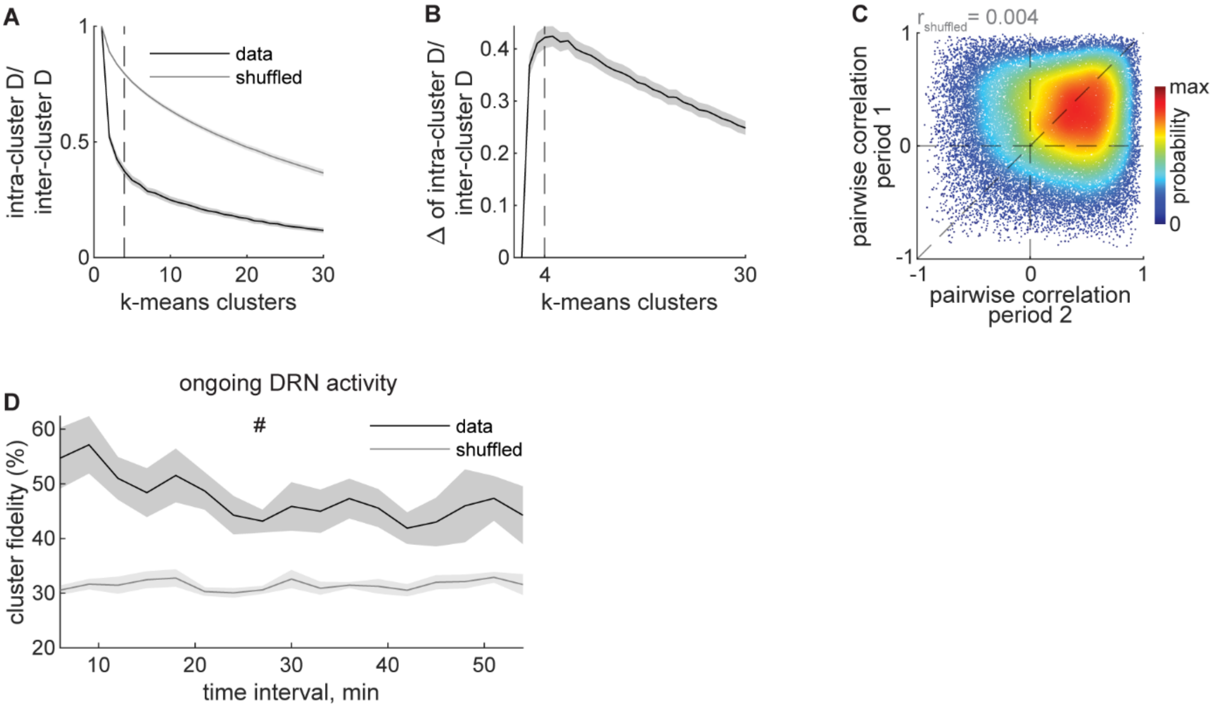

Figure S 1: Dorsal raphe exhibits structured ongoing activity. Related to Figure 1.
(A) Identification of optimal number of k-means clusters of ongoing dorsal raphe activity, by using elbow analysis. Elbow analysis calculates the sum of intra-cluster distances “D” for each cluster element, normalized by the sum of average inter-cluster distances for actual data (black), and simulated data (gray,100 iterations) with the same variance as the actual data but no cluster structure, in $n = 12$ zebrafish. This calculation is repeated for up to 30 k-means clusters (x-axis). The optimal number of clusters corresponds to the elbow point, where the black curve exhibits a prominent bend. Dashed lines indicate k-means analysis for 4 clusters. Line represents mean, shading represents SEM.
(B) Optimal number of clusters is further revealed, when actual data is compared to simulated data with similar variance but no cluster structure, by taking the difference of two curves in panel A. Note that the peak point of this difference reveals 4-5 optimal number of clusters in ongoing dorsal raphe activity. In this paper, we chose 4 clusters for k-means analysis of dorsal raphe activity. Line represents mean, shading represents SEM.
(C) Pairwise correlations of dorsal raphe neuron activity during two consecutive ongoing activity periods shuffled for pair identities (for data in Figure 1H). pde: probability density estimate. Shuffled data exhibit a correlation of $r_{shuffled} = 0.004$ for the pairwise correlation across two time periods. Orthogonal dashed lines: zero-correlation lines. Diagonal dashed line: unity line.
(D) Stability of dorsal raphe clusters is investigated by calculating cluster fidelity of neurons (mean ± SEM) in the first 3 minute in comparison to consecutive 3-minute time bins, in real data (black), and in simulated data with shuffled cluster identities (100 iterations, in grey). Note that while cluster fidelity remains relatively stable (around 50%), simulated data with shuffled cluster identities shows low cluster fidelity across time. Real data always remain significantly higher than shuffled control. Line represents mean, shading represents SEM, n = 8 zebrafish. ($p < 0.05$ or $p < 0.01$ indicated with #, two-sided Wilcoxon signed-rank test, no adjustments were made for multiple comparisons).

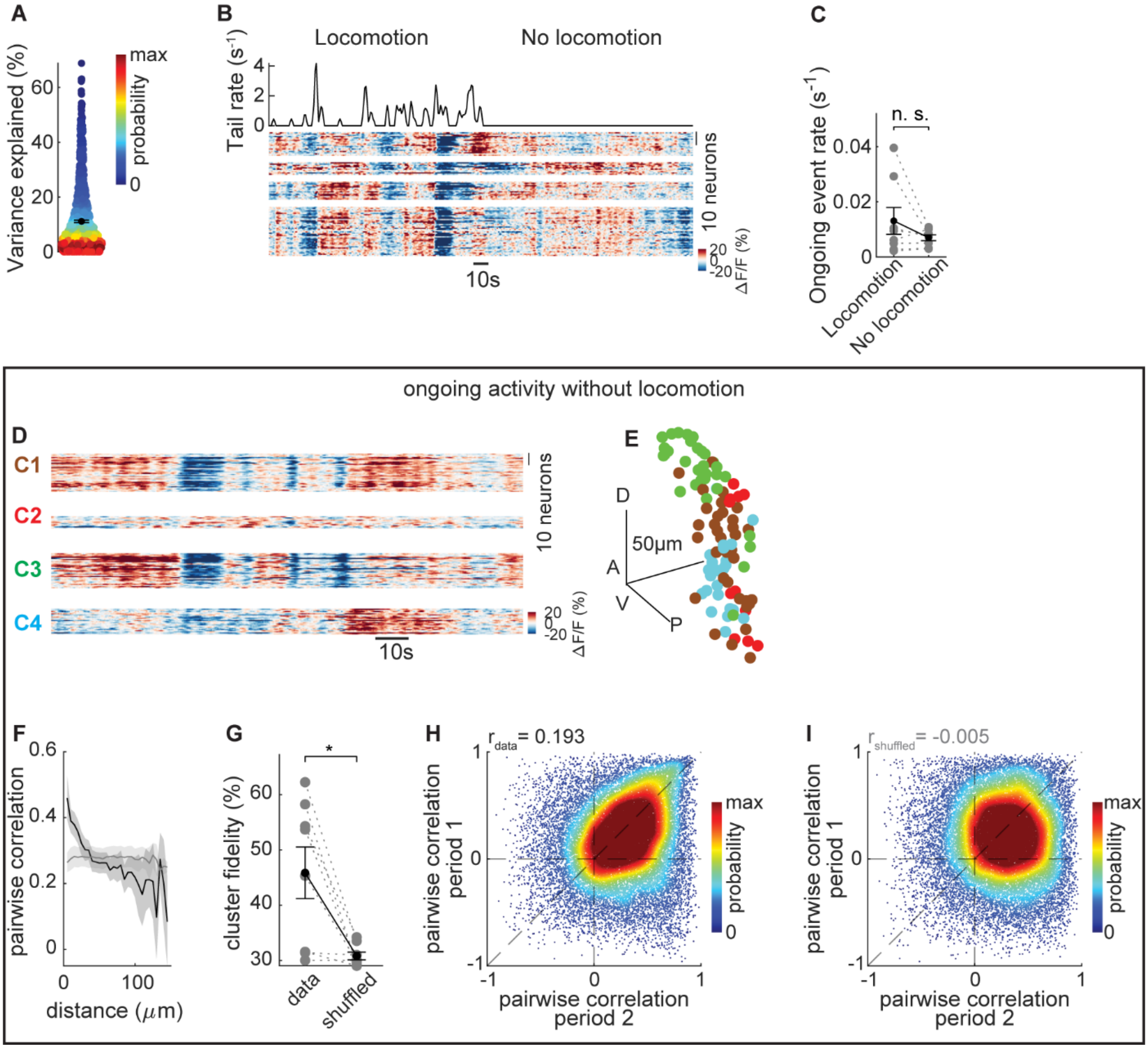

Figure S 2: Dorsal raphe exhibits structured ongoing activity during quiescent periods with no locomotor activity. Related to Figures 1 & 2.

(A) Percentage of variance explained by tail-bouts in every neuron. Probability density for neurons is color coded. n = 12 fish, mean ± SEM is shown in black.

(B) Example trace of locomotion measured as tail-bout rate in head-restrained juvenile zebrafish (top). Simultaneously measured dorsal raphe activity using two-photon calcium imaging (bottom). Warm colors represent higher calcium signals.

(C) Frequency of calcium events detected during locomotion versus no locomotion. Note that no significant difference was observed between these two epochs (n = 8 fish, p = 0.4609 two-sided Wilcoxon signed-rank test). Error bar represents mean ± SEM.

(D) Ongoing dorsal raphe activity recorded using two-photon calcium imaging in *Tg(Gad1b:dsRed ; tph2:Gal4 ; UAS:GCaMP6s)* zebrafish during quiescence periods with no locomotion. Dorsal raphe ensembles are clustered (C1–4) using k-means clustering. Warm colors represent higher calcium signals.

(E) Three-dimensional reconstruction of dorsal raphe ensembles (k-means functional clusters). Neurons are color-coded based on their cluster identities shown in panel D. A: anterior, P: posterior, D: dorsal, V: ventral.

(F) Pairwise Pearson's correlation of dorsal raphe neurons during ongoing activity (quiescence) as a function of distance (μm) between each neuron pair. Light-gray line

represents shuffled spatial distribution. Line represents mean, shading represents SEM (n = 8 fish).
(G) The ratio of dorsal raphe neuron pairs remaining in the same functional clusters during two consecutive ongoing activity (without locomotion) periods (high cluster fidelity) is significantly higher than chance levels. (n = 8 fish). Error bar represents mean ± SEM. *p = 0.0156, two-sided Wilcoxon signed-rank test.
(H) Pairwise correlations of dorsal raphe neuron activity during two consecutive ongoing activity (without locomotion) periods. color: probability density estimate. The data exhibit a correlation of r_{data} = 0.193 for the pairwise correlation across two time periods, indicating robust synchrony between pairs of neurons. Orthogonal dashed lines: zero-correlation lines. Diagonal dashed line: unity line.
(I) Pairwise correlations of dorsal raphe neuron activity during two consecutive ongoing activity (without locomotion) periods shuffled for pair identities. color: probability density estimate. Shuffled data exhibit a correlation of $r_{shuffled}$ = -0.005 for the pairwise correlation across two time periods.

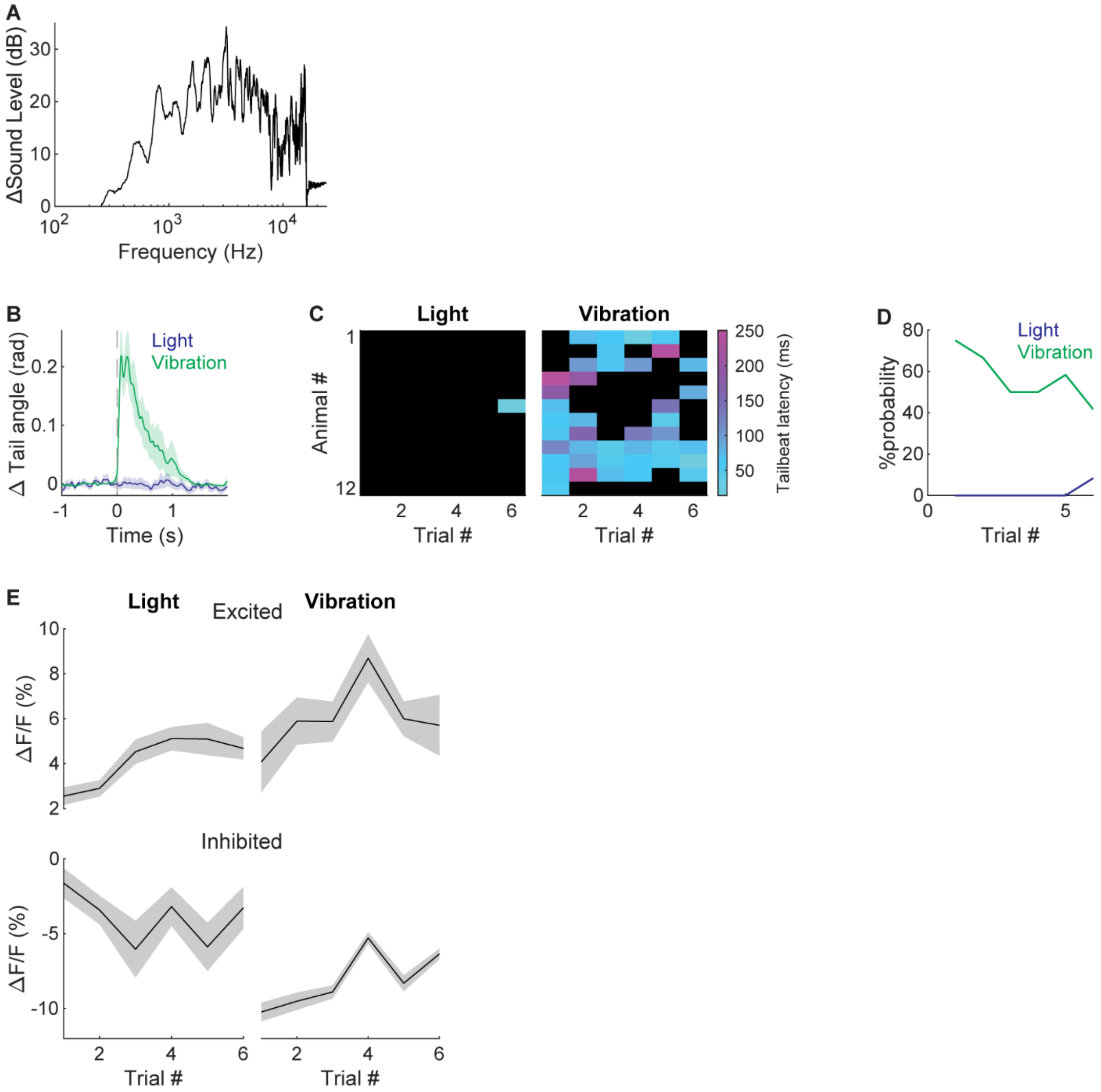

Figure S 3: Vibration, but not light, elicits reliable locomotor response accompanied by dorsal raphe response. Related to Figure 2.

(A) Frequency spectrum and amplitude of vibration stimuli.

(B) Time courses of average locomotor responses (change in tail angle) to light (blue) and to vibrations (green) in head-restrained juvenile zebrafish. Line represents mean, shading represents SEM. Vertical dashed line: stimulus onset.

(C) Locomotor responses and their trial-latency map for tail-bouts in response to light (left), and vibration (right). Each row represents an individual animal, and each column a trial. Tail-bout latency is color-coded. Trials without locomotory responses are blacked out.

(D) Probability of tail-bout response across trials for light (blue) and vibration (green).

(E) Average responses of dorsal raphe neurons classified as excited (top) or inhibited (bottom) during light (left), and vibration (right) stimuli across trials.

n = 12 fish, line represents mean, shading represents SEM.

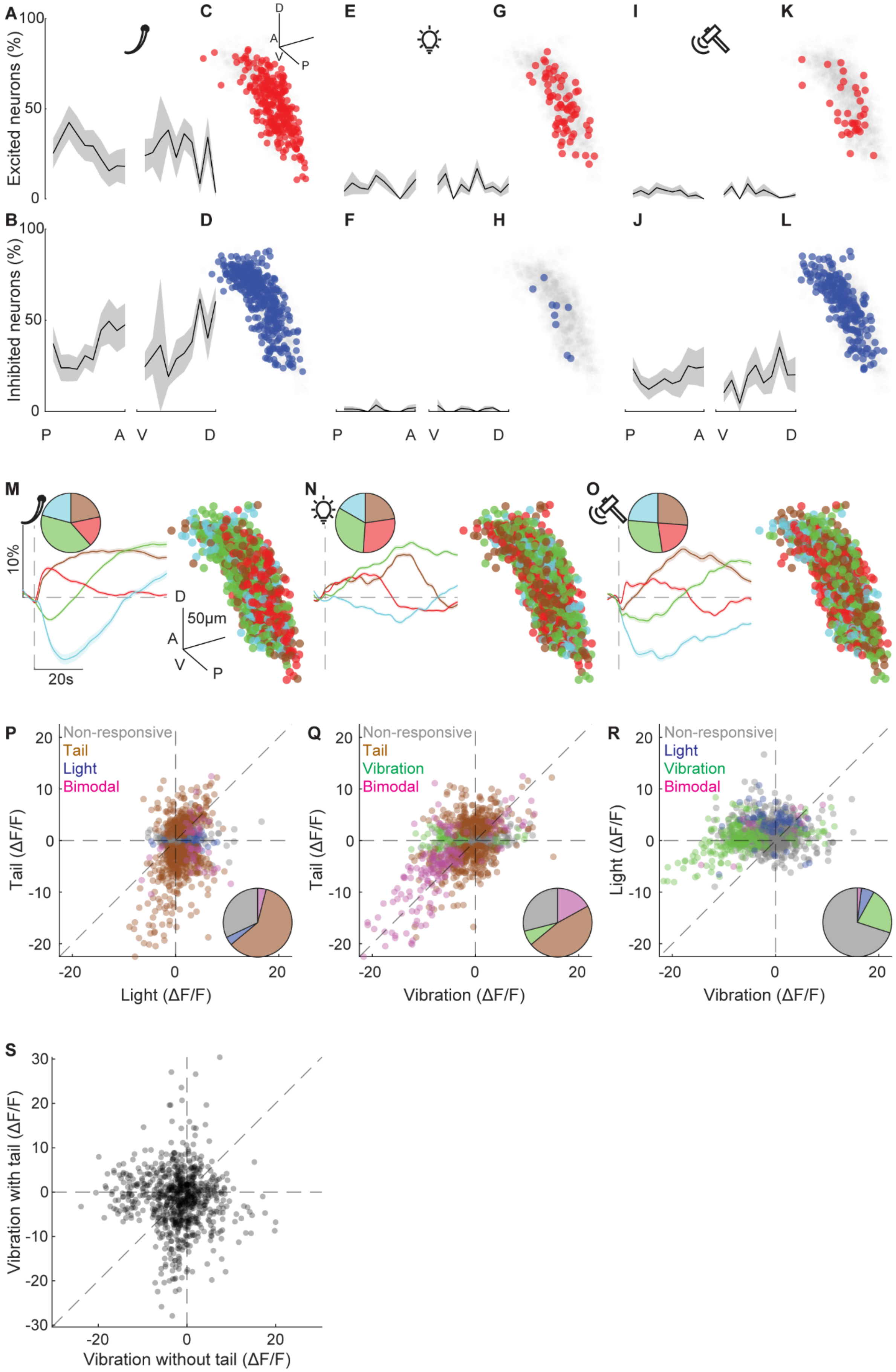

Figure S 4: Dorsal raphe responds to multiple stimulus modalities. Related to Figure 2.

(A-B) Fraction of significantly excited (A) and inhibited (B) dorsal raphe neurons upon locomotor tail-bouts with respect to their anteroposterior, and dorsoventral locations (number of excited/inhibited neurons within a particular location divided by the total number of neurons in that location). A: Anterior, P: Posterior, D: Dorsal, V: Ventral. Line represents mean, shading represents SEM.
(C-D) Spatial distribution (anterior: A, posterior: P, dorsal: D, ventral: V) of dorsal raphe neurons with significant excitation (C, red) and inhibition (D, blue) tail-bout responses. Data from all fish are spatially aligned and overlaid. Scale bar in panel C is 50 μm.
(E-H) Same analyses as in panels A-D during light responses.
(I-L) Same analyses as in panels A-D during vibration responses.
(M) Responses of dorsal raphe neurons in all fish (n=12 fish, n=976 neurons), during locomotor tail-bouts clustered by k-means clustering. The pie chart (top-left) represents the fraction of neurons in each color-coded k-means clusters. Time course of average responses (bottom-left) in each dorsal raphe k-means clusters. Line represents mean, shading represents SEM. Spatial distribution (right) of dorsal raphe neurons that are in different color-coded k-means clusters. Data from all fish are spatially aligned and overlaid. Scale bar is 50μm. Vertical dashed line: stimulus onset. Horizontal dashed line: zero-signal line.
(N-O) Same analyses in panel M, during light (N) and vibration (O) responses.
(P) Scatter plot of the mean responses of the dorsal raphe neurons for tail versus light, color-coded by their selectivity (gray for non-responsive, brown for tail exclusive, blue for light exclusive, magenta for bimodal neurons). The pie chart (bottom-right) represents the fraction of neurons in each color-coded category. Orthogonal dashed lines: zero-signal lines. Diagonal dashed line: unity line.
(Q-R) Same analyses as in panel P for tail versus vibration (Q), and for light versus vibration (R) (green for vibration exclusive neurons).
(S) Responses of all individual dorsal raphe neurons to vibration with and without tail-bout locomotory responses.

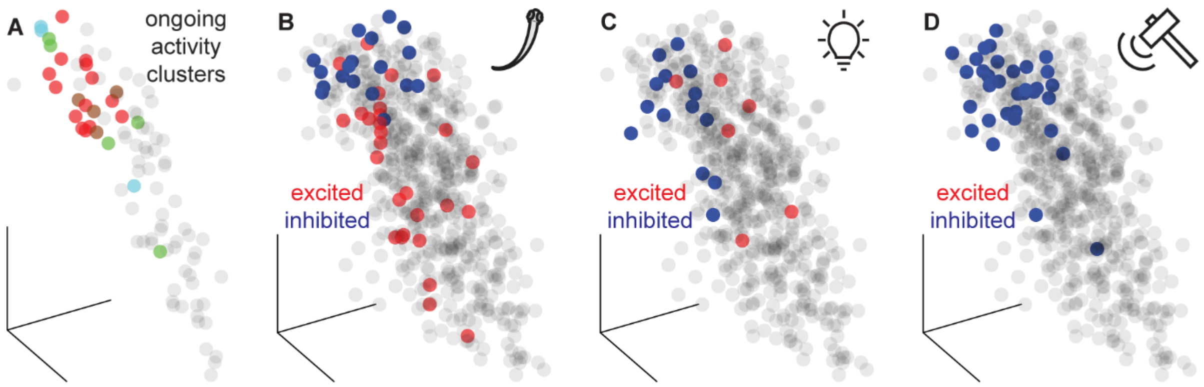

Figure S 5: Ongoing and evoked activity of *Gad1b*-labeled dorsal raphe neurons are topographically organized. Related to Figure 3.

(A) Spatial distribution of *Gad1b*-labeled dorsal raphe neurons grouped by functional clusters during ongoing activity in an example animal. Each color represents a distinct cluster identified by k-means analysis.

(B) Spatial distribution of *Gad1b*-labeled dorsal raphe neurons that are significantly excited (red) and inhibited (blue) by tail-bouts. Data from all fish are spatially aligned and overlaid.

(C) Same analysis as in panel B for light stimulation.

(D) Same analysis as in panel B for vibration stimulation.

Grey dots represent non-Gad1b:dsRed dorsal raphe neurons. Scale bars: 50 μm.

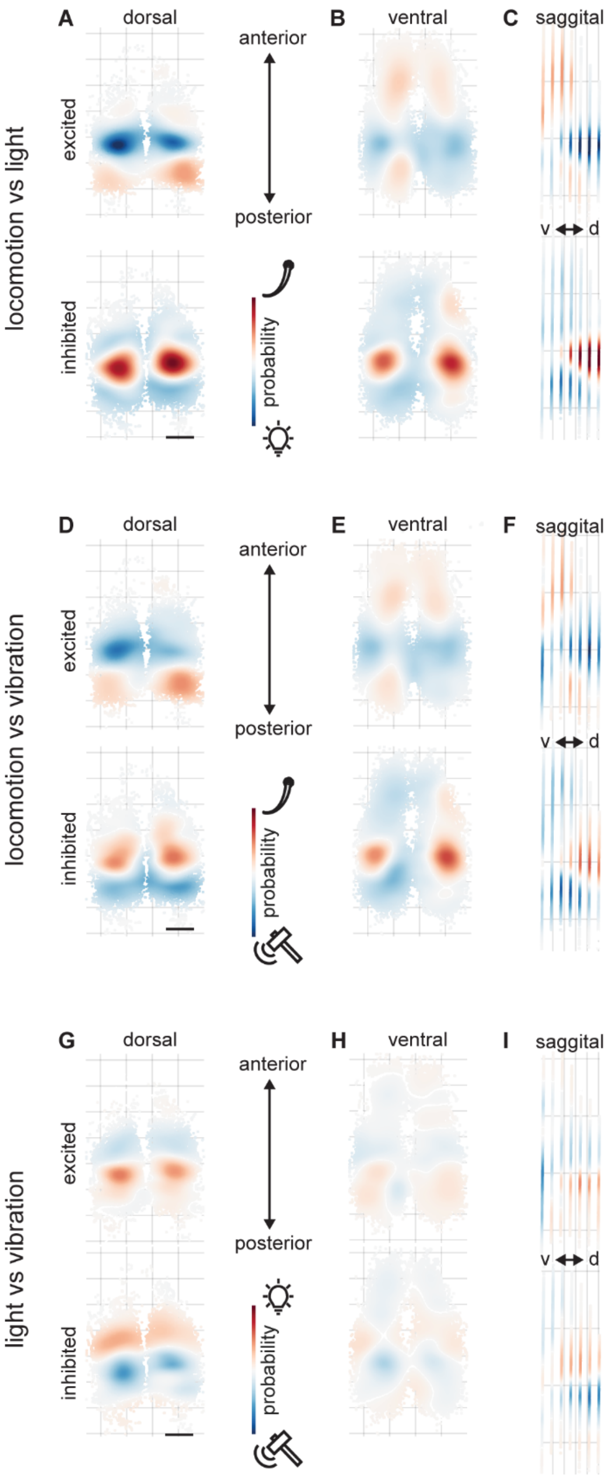

Figure S 6: Excitatory and inhibitory responses of the dorsal raphe axons to different stimuli are topographically organized in the forebrain.
Related to Figure 4.
(A-B) Difference between the spatial distribution of the tail-bout and light excited (top), and inhibited (bottom) axonal bins in dorsal (A) and ventral (B) forebrain. Red color highlights a spatial preference for the tail modulated axonal bins while blue color denotes a preference for the light modulated axonal bins.
(C) Sagittal views for the difference between the spatial distribution of the tail-bout and light excited (top) and inhibited (bottom) axonal bins per plane. Red color highlights a spatial

preference for the tail modulated axonal bins while blue color denotes a preference for the light modulated axonal bins. v: ventral, d: dorsal.
(D-F) Same analyses as in A-C for the tail-bouts versus vibration.
(G-I) Same analyses as in A-C for the modalities light and vibration.
Scale bars are 50 µm. *n* = 14 fish.

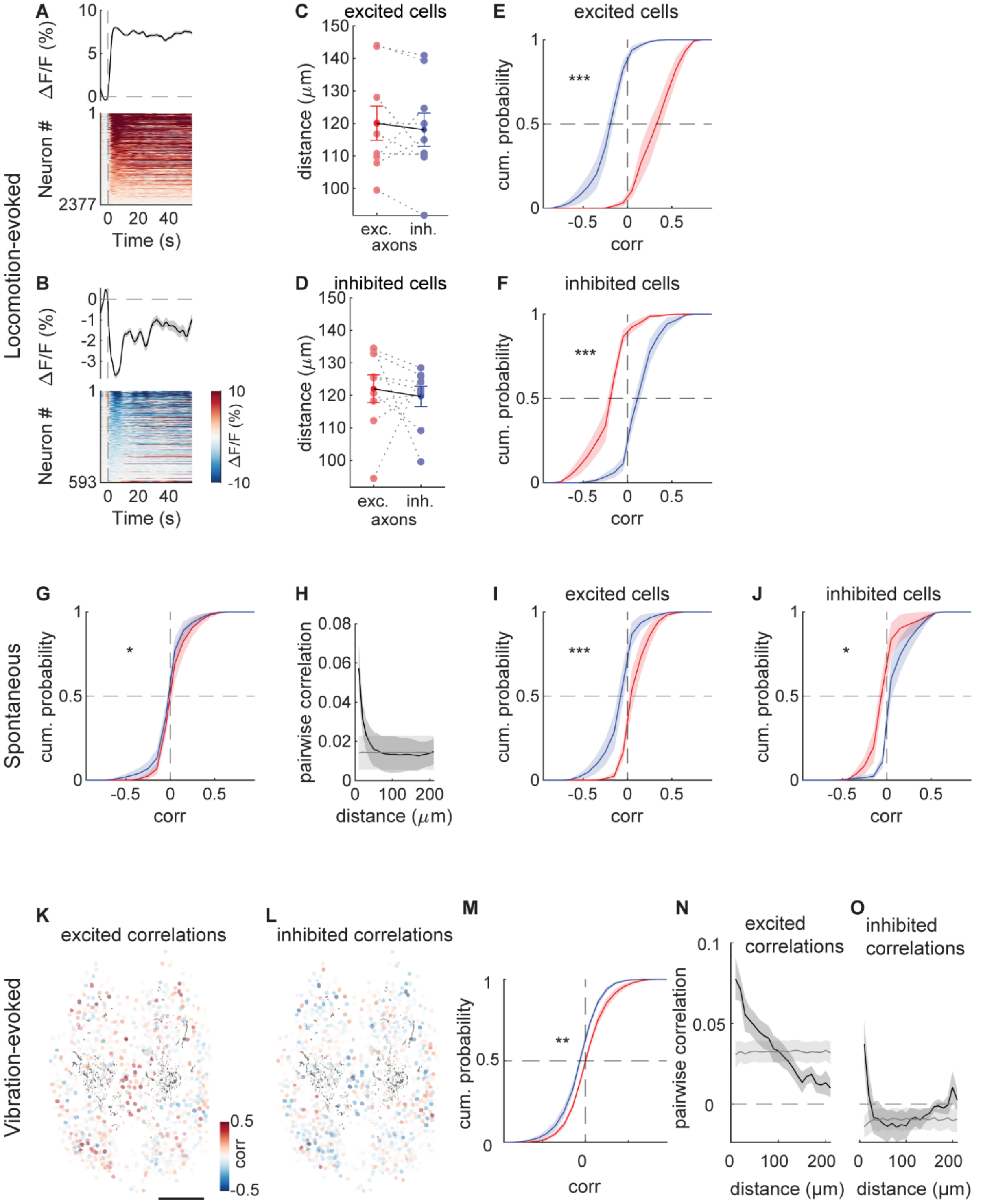

Figure S 7: Spontaneous, and locomotion and vibration related activities of dorsal raphe axons covary with forebrain neurons. Related to Figure 5.

(A-B) Tail-bout responses of excited (warm color) and inhibited (cold color) forebrain neurons. Line represents mean, shading represents SEM. Vertical dashed lines: stimulus onsets. Horizontal dashed lines: zero-signal lines.

(C-D) Average distances between excited (C)/inhibited (D) forebrain cells and excited (red)/inhibited (blue) dorsal raphe axons. Error bar represents mean ± SEM. No significant difference between the distances in C/D.

(E-F) Cumulative distribution for correlations between excited (E), and inhibited (F) forebrain neurons and excited (red) and inhibited (blue) dorsal raphe axons. Line represents

mean, shading represents SEM. Horizontal dashed lines: 50%-probability lines. Vertical dashed lines: zero-correlation lines. In E, ***$p = 2.2\times10^{-6}$, in F, ***$p = 3.5\times10^{-6}$ using linear mixed-effects model.
(G) Cumulative distribution for correlations between forebrain neurons and tail-excited (red) and tail-inhibited (blue) dorsal raphe axons during the spontaneous activity period. Line represents mean, shading represents SEM. *$p = 0.0108$ using linear mixed-effects model.
(H) Pairwise Pearson's correlation between forebrain neurons and dorsal raphe axonal region of interests as a function of their distance (µm) between them. Line represents mean, shading represents SEM. Gray line represents shuffled spatial distribution.
(I-J) Same analyses as in E-F during the spontaneous activity period. $n = 9$ fish. In I, ***$p = 0.0006$, in J, *$p = 0.0294$ using linear mixed-effects model.
(K-L) Pearson's correlations of activity between forebrain neurons and vibration excited (K) and inhibited (L) dorsal raphe axons. Black lines mark the dorsal raphe axons.
(M) Cumulative distribution for correlations between forebrain neurons and excited (red) and inhibited (blue) dorsal raphe axons. Line represents mean, shading represents SEM. **$p = 0.0078$, using linear mixed-effects model.
(N-O) Pairwise Pearson's correlation between forebrain neurons and vibration excited (N) and inhibited (O) dorsal raphe axonal region of interests as a function of their distance (µm) between them. Gray line represents shuffled spatial distribution. Horizontal dashed lines: zero-correlation lines. $n = 8$ fish. Scale bar represents 100µm. Line represents mean, shading represents SEM.

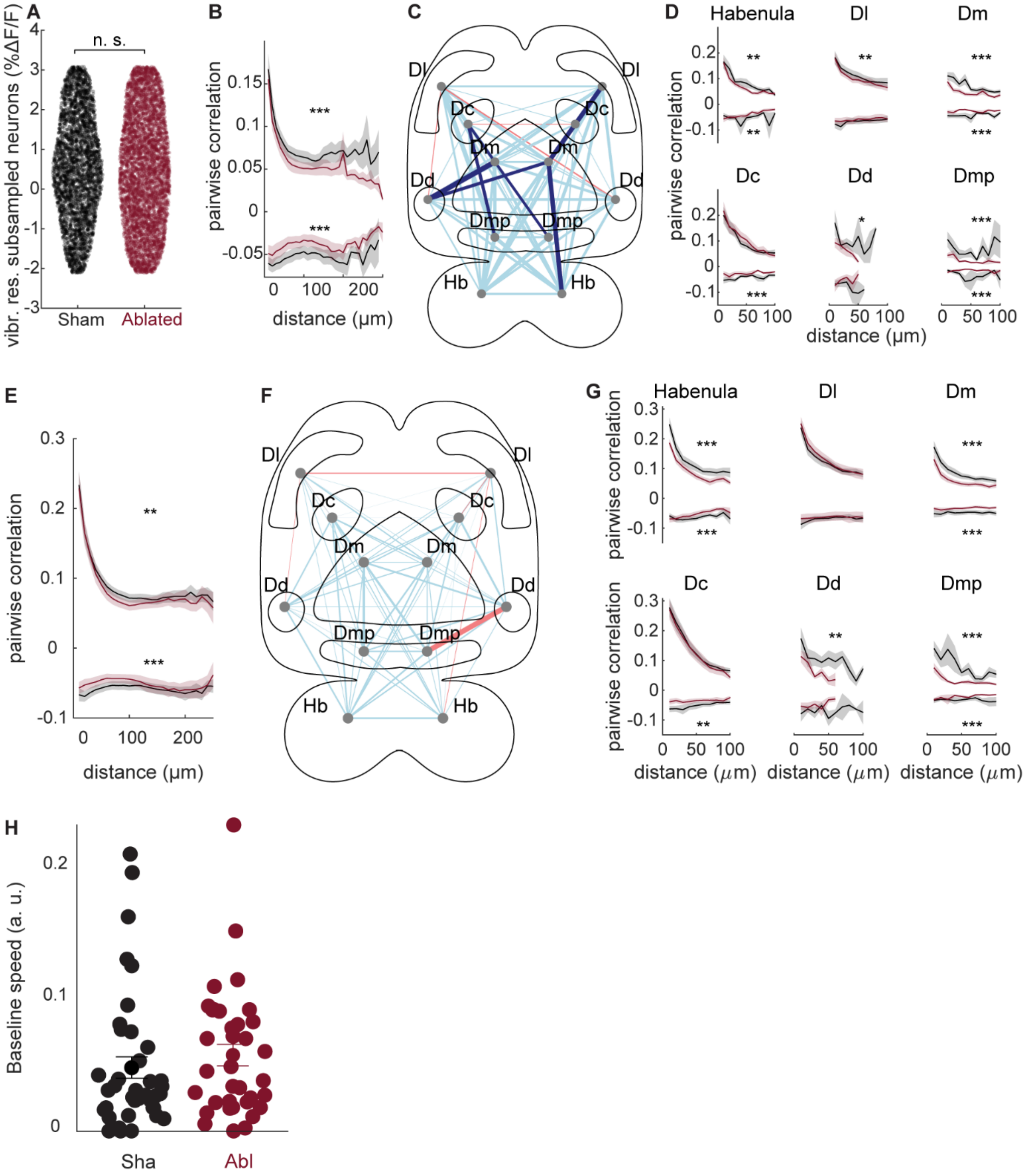

Figure S 8: Ablation of dorsal raphe impairs forebrain synchrony during vibration responses, and ongoing activity periods; and does not affect spontaneous swimming speed in head-restrained juvenile zebrafish. Related to Figure 6.

(A) Average vibration responses for response-matched forebrain neuron, with no significant difference between the sham (black) and dorsal raphe ablated (brown) zebrafish, by two-sided Wilcoxon ranksum test.

(B) Pairwise positive and negative Pearson's correlations of the response amplitude matched-forebrain neurons as a function of their distance (µm) between, in sham (black, n=16 fish) and dorsal raphe ablated (brown, n=16) head-restrained juvenile zebrafish. Line represents mean, shading represents SEM. Note an overall significant reduction of both positive and negative correlations. ANOVA displayed significance over the groups $***p = 1.1\times10^{-8}$ (positive), $***p = 2.4\times10^{-9}$ (negative).

(C) Schematic illustration of alterations in functional connectivity across response-matched forebrain neurons upon chemogenetic dorsal raphe ablation, during vibration-evoked activity. The locations of anatomically identified dorsal forebrain regions are marked with grey dots and abbreviated. The thickness of individual lines represents the average difference in correlations between thousands of individual neurons across forebrain regions. Cyan lines represent a decrease in average correlations, and blue lines represent a significant decrease in average correlations. Light red lines represent an increase in average correlations. Dorsal raphe ablated: n=16 fish and 4894 neurons; sham: n=16 fish and 3655 neurons. Dc: Dorsal-central telencephalon, Dd: Dorsal-dorsal telencephalon, Dl: Dorsal-lateral telencephalon, Dm: Dorsal-medial telencephalon, Dmp: Dorsal-medial-posterior telencephalon, Hb: Habenula.

(D) Pairwise positive and negative Pearson's correlations of the selected neurons within identified forebrain regions as a function of their distance (µm) between, in sham (black) and dorsal raphe ablated (brown) zebrafish. Line represents mean, shading represents SEM. Hb: $**p = 0.0020$ (positive), $**p = 0.0023$ (negative); Dl: $**p = 0.0010$ (positive); Dm: $***p = 1.1\times10^{-6}$ (positive), $***p = 1.3\times10^{-6}$ (negative); Dc: $***p = 2.3\times10^{-6}$ (negative); Dd: $*p = 0.0277$ (positive); Dmp: $***p = 2.0\times10^{-8}$ (positive), $***p = 3.0\times10^{-5}$ (negative) according to ANOVA.

(E-G) Same analyses as in B-D for all forebrain neurons, during the spontaneous activity period. ANOVA displayed significance over the groups, in E, $**p = 0.0057$ (positive), $***p = 0.0008$ (negative), in G, Hb: $***p = 9.9\times10^{-11}$ (positive), $**p = 1.2\times10^{-5}$ (negative); Dm: $***p = 5.9\times10^{-8}$ (positive), $***p = 2.1\times10^{-5}$ (negative); Dc: $**p = 0.0030$ (negative); Dd: $**p = 0.0039$ (positive); Dmp: $***p = 2.2\times10^{-8}$ (positive), $***p = 0.0001$ (negative).

(H) Average locomotion speed during the baseline period for sham (black, $n = 40$), and ablated (brown, $n = 35$) zebrafish. Error bar represents mean ± SEM.

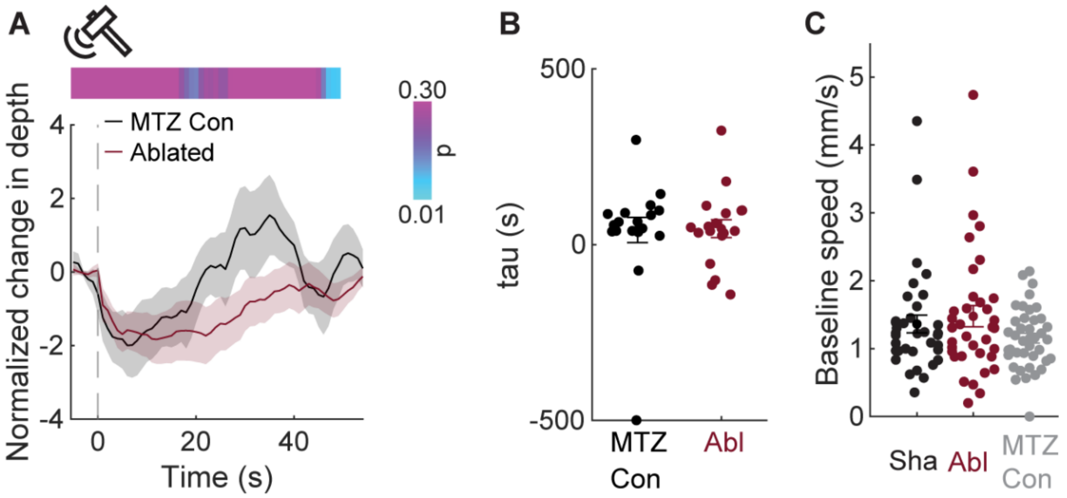

Figure S 9: Dorsal raphe ablation impairs behavioral response to vibration without affecting baseline swimming speed. Related to Figure 7.

(A) Average time course of change in depth normalized to the most bottom position reached in response to mechanical vibrations in MTZ-control (black, n = 25), and dorsal raphe ablated (brown, n = 24) juvenile zebrafish. p-values for comparison of the average normalized change in depth across consecutive 5 s windows between the groups are color coded at the top (two-sided Wilcoxon ranksum test). Note that there is no significant difference in recovery after animals reach their deepest swimming locations. Line represents mean, shading represents SEM. Vertical line: stimulus onset.

(B) Recovery time constant (tau from the exponential fit) for individual zebrafish during mechanical vibration response. Animals with goodness of fit > 0.1 are included, n = 18 for both MTZ-control (black), and dorsal raphe ablated (brown) zebrafish. Error bar represents mean ± SEM.

(C) Average free swimming speed during baseline for sham (black, n = 35), ablated (brown, n = 36), and MTZ-treatment control (grey, n = 39) animals in the novel tank test. Error bar represents mean ± SEM.
