# Supplementary material for: Topographically organized dorsal raphe activity modulates forebrain sensory-motor representations and contributes to defensive behaviors
Source: Nat Commun. 2026 Jul 16;17:6243. doi: 10.1038/s41467-026-75490-y (PMC13376501; doi:10.1038/s41467-026-75490-y)
Supplement: Supplementary file 2 — Description of Additional Supplementary Files [file 41467_2026_75490_MOESM2_ESM.pdf]

Title: Supplementary Video 1

Description: Functional imaging of dorsal raphe neurons and locomotor activity in head-restrained juvenile *Tg(tph2:Gal4; UAS:GCaMP6s)* zebrafish. The movie shows simultaneous monitoring of two-photon calcium imaging of dorsal raphe neurons and locomotor activity. On the left, the first panel displays the raw GCaMP6s fluorescence in the dorsal raphe. The second panel shows the corresponding ΔF/F calcium signals, highlighting neurons with increasing (warm colors) or decreasing (cool colors) activity. The third panel shows a bottom view of the tail and animals body used to extract locomotor behavior in the head-restrained zebrafish. On the right, time courses of neural activity (ΔF/F) in dorsal raphe neuronal clusters identified by k-means clustering. Below neural activity time courses, please find traces of x and y correction performed during the image registration and alignment process in suite2p. The bottom trace shows the locomotor activity of head-restrained zebrafish, allowing visualization of the relationship between dorsal raphe neuronal activity and tail movements.
