# Supplementary material for: Topographically organized dorsal raphe activity modulates forebrain sensory-motor representations and contributes to defensive behaviors
Source: Nat Commun. 2026 Jul 16;17:6243. doi: 10.1038/s41467-026-75490-y (PMC13376501; doi:10.1038/s41467-026-75490-y)
Supplement: Supplementary file 5 — Transparent Peer Review file [file 41467_2026_75490_MOESM5_ESM.pdf]

nature portfolio

Peer Review File

Topographically organized dorsal raphe activity modulates forebrain sensory-motor representations and contributes to defensive behaviors

Corresponding Author: Professor Emre Yaksi

This file contains all reviewer reports in order by version, followed by all author rebuttals in order by version.

Version 0:

Reviewer comments:

Reviewer #1

(Remarks to the Author)
- Summary -
The paper "Topographically organized dorsal raphe activity modulates forebrain sensory-motor computations and adaptive behaviors" by Mutlu et al., investigates sensory responses and responses to ongoing behavior in the dorsal raphe of zebrafish. The authors combine visual and mechanosensory stimuli as well as recorded spontaneous behavioral responses to investigate the functional and spatial organization of the dorsal raphe nucleus (DRN). The authors find that raphe neurons respond to these simple stimuli, which is perhaps not surprising given the established roles of serotonin in movement vigor and sensory arousal. Using the amenability of their preparation to calcium imaging the authors demonstrate that different neuron response types spatially segregate while correlations of responses of raphe axons in the forebrain with neighboring forebrain neurons suggest a functional influence on forebrain circuits. Chemogenetic ablations of the DRN furthermore underline this functional influence on forebrain activity and implicate DRN neurons in exploratory and anxiety linked behaviors. The paper is certainly interesting and sheds new light on the organization of the raphe; however, its writing appears rushed which makes it tedious to read in parts and the methods are currently inadequate to judge rigor of the approach. I furthermore have concerns about some of the analyses used and conclusions drawn which must be addressed before publication.

- Main points -
Issues with response selectivity
The authors make claims about the response selectivity of DRN neurons (e.g. Figure 2), however, I cannot find any data that speak to the relationship of stimuli and behaviors. E.g. what fraction of taps elicits a behavioral response? Has care been taken to isolate sensory stimuli from behavior? It is not at all explained in the methods, how a neural response is labeled as tap vs. light vs. swim. I assume that most taps elicit swims and it is unclear whether such episodes have been removed from the displayed responses. The authors need to show the frequency of behavioral responses to the varied stimuli and discuss whether episodes with behavioral responses were removed from the analysis and if not, why not.
On a related note, the legend of Figure 5 talks about "Tail-beat" responses but the symbols used and the main text suggests that taps were used. Is this because these are one and the same thing (i.e. each tap leads to a tail-beat response)?

Issues with interpretation of correlations
1. The authors find a modest decrease in forebrain activity correlations (both positive and negative) after DRN ablations (Figure 6B-D) as well as a decrease in overall telencephalic responses (Figure 6E-H). Since calcium imaging is limited by shot-noise the former can trivially be explained by the latter. As responses decrease, correlations will naturally decrease significantly even if no circuit properties are altered simply due to the sensitivity of correlations to the signal-to-noise ratio. Significance in the reduction of correlations therefore has to be evaluated compared to losses in correlation in a shot-noise limited model when comparable reductions in peak amplitude are observed. The current significance test is insufficient. Absent this the data should be moved to the supplements and it should be made explicit in the text that the reduction in correlations may be a consequence of the reduction in response amplitude. I don't think doing this takes away from the paper but will improve rigor.
2. The authors claim (Page 6) "… whereas inhibited DRN axons displayed stronger negative correlations with neurons in

their vicinity (Figure 5H-I).” It is unclear to me how this conclusion was reached. 5H indeed suggests that proximity to axons that respond with excitation to tail-beats (taps?) have higher positive correlations, but 5I essentially shows correlations that are not significantly different from the shuffles, except maybe at distance 0. However, these are still not “stronger negative correlations” considering that they are smaller than most observed excited correlations. I might be misreading here what the authors want to say.

* Issues with region responses *
To identify changes in the relationship of forebrain activity to behavior after DRN ablation the authors are “relating the neural response of each forebrain region to animals’ behavioral responses.” It is unclear to me what that means. Does this refer to average activity across the entire region? If yes, why was that choice made? In that case, a loss of responsiveness does not have to mean that neurons within the region respond less, but it could simply mean that the balance between excited and inhibited neurons changed. A more appropriate approach would be to perform per-neuron fits of the behavior-activity relationships and report the distributions of slopes between the ablated and non-ablated conditions. In a similar vein, since the authors are effectively interested in the change in slope it is unclear to me why they chose to fit the model with the interaction terms instead of fitting models separately on the ablated and control data. Approaching this per neuron would overcome this issue as well.

* Issues with clarity of methods *
The methods section is currently lacking in detail and clarity, especially with respect to data analysis:
1. The definition of behavioral vs. sensory responses is not given.
2. It is unclear what the text “In Figure 4…” [page 14] means
3. It is unclear what the ICA was used for in Figure 5 and why it was performed. How do independent components chime with the ROI count in Figure 5? What does it mean to be “manually inspected and verified?”
4. There needs to be a clear description on how the different control conditions are generated.

- Minor points and textual problems -
A seeming lack of proofreading and checking figure/section references made this manuscript frustrating to read at times. I suggest that the authors carefully check the text but I am listing some points that jumped out to me below:

P2: Vglut3/Trh/Gad1/Gad2 are abbreviated on first mention

P2 and discussion: I’m not a fan of references to “ancient cortico-limbic” structures or cortex homologs. The cortex has a very specific cellular organization as its defining feature and absent that it’s evolutionary origin does not suggest functional similarity.

P3: Unclear what “sensory-motor events” refers to – are these events of coincidence of some sensation and action? Or does it refer to both “sensory or motor related responses”

P3: “that are either inhibited or excited … or sensory-evoked activity” – what does this mean. If there is “sensory evoked activity” then it is by definition excitation. Or does this refer to sensory-evoked behavior?

P3: “… were inhibited by each tail-beat” – I do not think a calcium-indicator could resolve this. Do the authors mean “swim” as a collection of tail-beats?

Page 4 Figure S1 panel references are wrong – they point to the wrong panels and the intended panels appear missing.

Page 5: “we observed that mechanical vibrations” – but figure legend talks about tail beats. Figure symbols on the other hand do suggest mechanical vibrations. Which is it?

Page 6: “Such recovery requires a level of cognitive….” – this sentence contains a lot of strong words for a test that could rely on simple habituation. That doesn’t take away from the experiments performed, but as currently written (e.g., “cognitively demanding”) the sentences seem completely disconnected from the experimental tests.

Figure 1H: I don’t think the shuffled data adds much to this panel here but instead makes it confusing. To properly evaluate the shuffle result would require a KDE since it is near impossible to judge dot density especially with the overlaid KDE of the non-shuffled data. I suggest removing the shuffles and instead show a KDE of the shuffles (on the same color scale as the true data) in the supplements.

Figure 2H: This panel is unclear since different colored dots would hide each other so AP and DV cannot be read out at the same time. I suggest a split into two panels. The same is true for other overlays.

Figure 5: As mentioned above for C-H there is a disconnect in legend (“tail-beat responses”) and symbols used (“vibration stimulus”)

Figure 7A: This plot should be normalized to the bottom position reached to ensure that the results in 7C aren’t causing the apparent slower recover in 7A. In other words, the statement made for 7A is a statement about slopes and a normalized display would help make that point.

Figure 7J: What is the N in this figure and what are the statistics calculated across?

(Remarks on code availability)
URL was not reachable but simply led to a 404 error. This is unfortunately in line with my general impression of the paper being rushed and not properly proofread.

Reviewer #2

(Remarks to the Author)
This is a very nice study that identifies topographic features of the zebrafish dorsal raphe during baseline and in specific behavioral contexts, and relates these features to neuronal activity in the forebrain. The experiments are clearly described and presented, and are interpreted in a reasonable manner. The experiments yield clear and interesting answers to the questions posed. Overall, this is a solid contribution to what is rapidly becoming a highly studied area of neuroscience. I have only minor suggestions for improvement, outlined below:

1. Page 3: Please fix this error on page 3: (Error! Reference source not found.A, B)
2. Figure S1C,D: I'm not sure what this is showing. Does % of excited neurons represent the number of neurons that show excitation divided by the total number of raphe neurons that were imaged (i.e. 80 neurons)?
3. Fig 3A,B: the authors state that ~25% of DRN neurons are Gad1b positive, but in the images shown it look like most of the DRN neurons are Gad1b positive. Is this because the images only show focal planes where there is a high level of co-expression? If this is the case, it would be better to show images of all DRNs to better convey the conclusion that only 25% of cells are co-labeled.
4. Fig 3F,J,M,P: I think it would be useful to also show color-coded clusters for only the Gad1b+ DRNs, which would give spatial information about Gad1b+ clusters that is not provided by the graphs shown in panels K,N,Q.
5. Fig 4. The 2D histograms are a nice way to convey the spatial information. It would be interesting to see a comparison between the different behavioral contexts (i.e. tail movement, response to light, response to vibration) and the location of excited and inhibited fibers in the forebrain. For example, The inhibited fibers for tail movement and vibration stimulus look grossly
similar, but can this be quantified?
6. Fig 5C,D: The image appears to show vibration stimulus data, as stated in the main text, but the figure legend states this is tail-beat data.
7. Fig 5E-I: it would be interesting to know if the forebrain inhibited neurons are more spatially correlated (and if forebrain excited neurons are anti-correlated) with nearby Gad1b positive DRN axons.
8. Fig 6D: While the differences in correlation for sham vs. ablated are statistically significant, the differences appear to be very small. I suggest that the authors note this, or describe why they think the changes are large enough to be meaningful.
9. Fig 6J: While the differences are significant, they appear to be driven by a very small number of outlier data points. Do the authors agree with my interpretation? If they agree, I would soften the language of the text to reflect this.
10. Fig S2A: again, the differences are significant but apparently very small, so it's unclear how meaningful the differences are.

(Remarks on code availability)
I lack the skills needed to evaluate the code.

Reviewer #3

(Remarks to the Author)
The study captures interesting functional diversity in the DRN population in response to mechanical and visual stimuli. The results regarding the functional and anatomical diversity of DRN are convincing and valuable to the community.
The effects of DRN ablation on behavior (e.g. increase in sensory-motor response) are also credible. However, these results lack clarity in the presentation and, more importantly, are overstated to imply that the behavioral effects are due to DRN-forebrain interactions.
The most unconvincing claims concern the behavioral consequences of DRN-forebrain interactions. Overall, the latter half of the results sections presents a number of conclusions that are not supported by sufficient evidence.
In summary, the manuscript consists of three main parts – DRN diversity, behavioral phenotypes following DRN ablations, and forebrain sensory-motor activity changes in response to DRN ablations. However, the relationship between these three parts is unclear. At times, the authors seem to want to imply that there is a coherent narrative by simply putting results next to each other in a figure (e.g. Figure 6 and comment #5 below). However, to actually support these claims, deeper analyses and significant additional controls are necessary.
Finally, there are too many instances where basic experimental and statistical details (e.g. standard deviations, duration of time windows, animal numbers, meaning of lines on plots, etc.) are missing.

Major comments:
1. The manuscript strongly implies that changes in "DRN-forebrain interactions" are responsible for the behavioral results in Figure 7. In the set-up for the experiments in Figure 7, the authors state that "This cognitively demanding and adaptive behaviors are often attributed to cortico-limbic structures in the vertebrate forebrain".

However, there is no evidence that the effect of DRN ablation on diving behavior has anything to do with the forebrain. Figure 7 shows a real effect of DRN ablations -- increased diving depth in response to stimuli. However, this could have been generated entirely independently of changes in forebrain activity. The DRN projects broadly throughout the brain. Other brain regions (e.g. optic tectum, hindbrain) could be the source of the observed behavioral change.
The only way to potentially support the claim that DRN-forebrain interaction has a causal role in modulating diving behavior is to demonstrate that forebrain ablations uniquely alter the diving depth, or better still, specifically ablate the DRN projection to the forebrain. Unless either of these experiments can be performed, the claim about DRN-forebrain interactions in the context of the diving behavior is entirely speculative.
2. The usage of the term “adaptive behavior” throughout results related to Figure 7 is odd. “Adaptive” in what sense? The DRN ablated animal dove to a great depth (consistent with the strong tap responses shown in Figure 6i). In this case, it would of course take the animal longer to re-surface. The post-ablation plot in Figure 7a looks like the control plot plus a constant. The time window chosen for the analysis seems arbitrary.
3. The most credible takeaway is that DRN ablation leads to greater behavioral responses to mechanical stimuli. Introduction to a novel chamber would also be associated with sudden mechanical, optical, and/or flow-associated stimuli, assuming the animal is dropped into the chamber by the experimenter. The current presentation of the results obscures what appears to be a simple point.
4. One can make a simple objection to the conclusions in Figure 6 – if the sensory-motor responses in the forebrain are weaker following DRN ablation, then the SNR of the cells are lower. More specifically, the noise floor of each neuron (e.g. due to shot noise) is unlikely to be affected by DRN ablation, but the real signal is now significantly lower in amplitude. Mathematically, this directly leads to decreased correlation between cells and has no bearing on whether the actual “synchrony” has changed.
Given the relatively modest change in Figure 6, one would need to be convinced that the effect is not simply due to changes in SNR. For example, one can sub-sample a set of cells with identical response amplitudes to stimuli pre- and post-ablation and demonstrate that these cells show loss in synchrony. If this isn’t the case, then the authors have simply shown that forebrain neurons have weaker sensory-motor responses, which is credible. Again, as in Figure 7, it seems that there is a real result in Figure 6, but one that has been unconvincingly overstated.
5. Figure 6i has the same issue as Figure 7. There is no supporting evidence for the claim that the decrease in forebrain sensory-motor response is responsible for a significant increase in motor responses. Again, to demonstrate that this is true, the authors would need to specifically ablate the DRN projection to the forebrain or at a minimum, demonstrate that forebrain ablations lead to increased sensory-motor responses.
By shoehorning this result into a figure primarily on forebrain responses, there is some implied suggestion that these observations are related to each other. However, unless the above control experiments are performed, there is no reason to expect that a real relationship actually exists.
6. It’s unclear what is the underlying phenomenon that drives the correlation patterns observed in Figure 5, or what is the significance of the result in 5h. The DRN axon primarily innervates a central neuropil region. For instance, let's assume that the forebrain brain neurons activated by taps are simply distributed around the neuropil region. Geometrically, if one mirrors this layout across the left-right axis, then one would trivially expect that the correlation will be distance-dependent. It’s unclear whether this reveals any relationship between DRN and forebrain neurons.
To show that there really is something non-trivial, the distance correlation should be done on one half of the forebrain (left or right) at a time. Furthermore, one would ideally like to have some clarity as to why and how the correlations would be stronger or weaker.
7. Claims about modality-specific distribution of functional responses in the DRN need more quantification or qualification. For instance, the authors state that “increased fraction vibration-evoked inhibitory responses observed towards the anterior-dorsal DRN”. However, this may be influenced by a variety of factors including stimuli strength and habituation. Mechanical and light responses adapt over time and adapt at different rates. How are these factors accounted for? How is the stimulus intensity chosen? Parametrically varying stimuli intensity would have been helpful, otherwise one needs to explicitly state that light at x intensity and tap at x intensity elicited the observed results, with justification for the levels chosen.
8. Just as stimulus strengths are unstated in the results section, a number of other experimental details are left out. For instance, the authors state that “Next, we assessed the stability of DRN clusters by quantifying the likelihood that pairs of DRN neurons remain in the same cluster during two consecutive time periods”, without actually specifying the time period. This can have consequences for the clustering analysis.
9. To what extent is the “ongoing” activity of DRN in Figure 1 a function of spontaneous locomotor activity in the animal? If that’s the case, then this figure doesn’t offer additional information that isn’t already covered in Figure 2.
10. Are there conjunctive coding of different stimuli (e.g. Figure 2). Neurons are classified as excited/inhibited/other in response to each stimulus. But there can be neurons with responses to both.

Minor comments:
1. For all statements on the percentage of neurons (e.g. “Across all recorded fish (n=12) 26% of DRN neurons were excited, ..”), it would be good to include mean and standard deviation across animals.
2. Unclear what is the definition of “baseline” for delta F/F calculations. Neural activity is generally shown as delta F/F, i.e. fractional change in fluorescence relative to “baseline”. In different parts of the Methods section “baseline” is described as 3 minutes, 30 minutes, or as “5s period before stimulus onset”. It is unclear during which period exactly the fluorescence baseline is established, and why such a range was used.
3. Time window information is repeatedly missing. In Figure 1, it appears that the pairwise correlation is calculated over the entire window of spontaneous activity (several minutes?), whereas in Figure 2 the pairwise correlation is calculated during "tail-beat responses", so on a trial basis over a couple of seconds (?). This is basic information that should be stated clearly.
4. In Figure 4, it is unclear why there are two vertical dashed lines in the plots. If they represent the stimulus on- and offset, one expects this to be clearly stated in a standard figure legend.
5. What is the animal number for Figure 6j? For panel b, the figure legend specifies n = 16. But there are clearly more than

16 points in 6j, and the animal number is not given in the figure legend for 6j.
6. How is the average response defined in Figure 6j? Each point represents an individual fish with several stimulations. It is not clear how the “average of average responses” is defined here. As average value of the averaged response until offset of stimulus or until decay of the neural/behavioral response?
7. It is not clear how exactly the “shuffled/simulated data with same mean and variance” is created.
8. In Figure 5 C and D the text speaks of tail-beat responses, but the image shows the mechanical vibration icon.
9. Are there difference in baseline tank depth preference (Figure 7)? Ablated fish seem to prefer a deeper tank depth prior to any stimulus delivery based on Figure 7A.
10. typos: - (Error! Reference source not found.A, B).
11. No line numbers

(Remarks on code availability)

Reviewer #4

(Remarks to the Author)
In this study, Mutlu et al. use a combination of functional imaging, molecular circuit analyses, and behavioral experiments to generate a comprehensive description of the raphe in juvenile zebrafish. They find that the raphe nuclei consist of distinct functional and molecular subtypes. They also show that behavior/stimulus excited and inhibited raphe axons have different spatial distributions in the forebrain. Furthermore, they demonstrate that ablation of serotonergic neurons in the raphe decreases sensorimotor responses in the forebrain. Finally, this study detects clear behavioral effects induced by serotonin manipulation. In sum, this study provides new insights into the functions of serotonin, extending previous research on the raphe. Some of the findings may also require additional validation to address potential artifacts of the experimentation.

Before I can recommend publication, it would be important that the authors address the following points:

Main points:
- Please clarify whether the animal was moving or not in response to the visual and tactile stimuli. This is important to separate motor, sensory, and sensorimotor effects (for example, Fig. 2J,O). A lot of the described stimulus-response activity could, in fact, just be related to movement. Similarly, it would be interesting to know if there are any stimulus-specific responses in the raphe that occur when animals do not move.
- Additional details on how the experiments were performed would also be helpful. For all figures, please clarify what was done volumetrically and what plane by plane. Are volumes always acquired dorsally to ventrally? Are planes always scanned in the same direction? How are the behavior and stimuli aligned to the imaging data? At which time during the scanning process? This information would help interpret the short-time-scale effects.
- The main point of Fig. 5 is that raphe axons are correlated with nuclear-tagged cell activity in the forebrain—which is a great analysis. However, there could be small pieces of axons in the cell’s ROI that could be responsible for the correlation. Showing un-thresholded components could help get a sense of whether this is a problem. Clarifying the xyz size of the voxels would also be helpful.

Minor points:

Figure 1:
- Fig 1B: please present non-saturated micrographs if possible.
- Fig 1C,D: is this from plane or volume recordings? This is important to interpret whether these are the total number of serotonin neurons in each fish.
- Since the reporter is cytosolic, it would be good to demonstrate the quality of the segmentation.
- Fig 1D: Was the fish tail free? What percentage of the variance is explained by the behavior? —this information is also not in Fig. 2 but is important for the claim “We discovered that a large fraction of DRN neurons is primarily driven by animal locomotor activity” from the abstract.
- Fig 1H: Cluster stability was tested only on short time scales (consecutive time periods); it would be more meaningful to test stability over longer time scales.
- To mitigate the risk of confounding spatial patterns with movement artifacts, more thorough documentation of raw data is recommended. For instance, the authors could provide supplementary material, such as traces of the movement correction parameters, indicating whether these occur on a different time scale than the activity. Additionally, an example video of the movement-corrected data, along with the corresponding activity response spatial pattern, could be shared.

Figure 2:
- Fig 2D: In the heatmap plot, it is unclear whether the cells are absent or zero; displaying the contour in the image could provide an understanding of the proportion of cells responding. Additionally, if non-responsive cells are found in the region, together with responsive ones, this would help exclude movement artifacts.

Figure 3:
- Fig 3A: It would be more helpful to the reader to show the 2-photon micrographs that were used for the rest of the figure, or clarify why the authors use confocal images here.
- Fig 3C: Cell assignment can be noisy due to resolution and expression variability. Explaining and maybe quantifying uncertainty in cell type assignment would help clarify if the variability across fish is biological or due to measurement.
- Fig 3H: Please clarify how the control was done (whether it was gad1b cell correlation with non-gad1b cells or correlation

between non-gad cells).

Figure 4:
- Please clarify whether the statistics are done on voxels or components.
- Fig 4G: Details on how the alignment between animals was made are missing. Is there a reference image channel, or did the authors just map spatially filtered versions of the axonal structures on top of each other? If the latter case, how precise would that be?
- Fig 4H: a side projection would be helpful to visualize segregation in depth.

Figure 5:
- Fig 5C,D: is there a mistake in the little drawing? Should this be a tail flick rather than a tap? In the legend, it says ‘tail-beat responses’.
- It would be useful to also show traces from the forebrain neurons for direct comparison with the axons.

Figure 6:
- Fig 6C: It is important to clarify whether the decrease in correlation between regions can be trivially attributed to their reduced activity. For the example, the authors could calculate the correlations for the activity of the control but scaled down to approximately the value found after the ablation.
- Fig 6J: There are more than 16 points, are these really one point per fish? Or are these behavior trials?

Figure 7:
- The author demonstrates in the initial section of the work that there are heterogeneous populations of tph-positive neurons involved. However, these populations are manipulated collectively in the behavioral experiments. This aspect requires further discussion.

Discussion:
- There has been a lot of recent data on the activity of the raphe in fish, it would be great to have a bit more detailed interpretation of the results in the context of these other studies (eg. psilocybin, behavior states).

(Remarks on code availability)
code access not available.

Version 1:

Reviewer comments:

Reviewer #1

(Remarks to the Author)
I thank the authors for carefully engaging with my comments and suggestions. I believe that the additional analyses and clarifications have increased the rigor of the manuscript substantially. I think that the claims currently made in the manuscript are supported by the data as presented. While I agree with reviewer #3 that experiments to link the different described phenomena (spatial organization, effects on forebrain activity, behavioral effects of DRN ablations) are lacking, I do not get the impression from reading the paper that these connections are implied. However, it might be useful if the authors explicitly acknowledge that DRN neurons project broadly throughout the brain and that future work will be necessary to tease these influences apart.

(Remarks on code availability)
The code is now fully available; however, I have not reviewed it since I do not have MATLAB installed.

Reviewer #3

(Remarks to the Author)
The authors have made some real attempts to improve the manuscript, such as the response-amplitude-matched subsampling for correlation analyses or the variance-explained analysis for locomotion.

However, a fundamental problem persists. The authors’ rebuttal claims they never asserted that DRN-forebrain interactions underlie the observed behavioral phenotypes, yet the manuscript’s title, structure, and framing continue to strongly imply exactly this causal chain. Until this disconnect is resolved, the manuscript cannot be recommended for publication.

In the rebuttal, the authors make the following statements:
“We did not intend to make any claim that ‘DRN-forebrain interactions’ are responsible for the behavioral results in Figure 7.”
“Given that we did not make such a claim, we hope that reviewer #3 will agree that proving a claim that we did not make (on specific impact DRN to forebrain connections in animal behavior) is not within the scope of this study.”
“Specific ablations of DRN to forebrain connections while performing behavioral tests in free-swimming animals, is not experimentally feasible … Hence testing the specific impact of DRN to forebrain connections in free-swimming behaviors in zebrafish is not a technically feasible experiment.”

I accept the authors' assertion that they cannot perform the pathway specific ablation experiment needed to substantiate the title of this paper “Topographically organized dorsal raphe activity modulates forebrain sensory-motor computations and defensive behaviors”. However, ablation of the forebrain itself has been established in previous publications.

But the bigger concern is that the authors deny making a claim while simultaneously preserving all the textual and structural elements that lead a reader to infer precisely that claim.

Specifically:
1. The title implies a causal chain through the forebrain
The current title is: “Topographically organized dorsal raphe activity modulates forebrain sensory-motor computations and defensive behaviors.” The syntactic structure of this title is clearly making an effort to imply that the DRN’s modulation of forebrain activity is the mechanism through which defensive behaviors are affected. If the authors genuinely believe that the behavioral effects may arise from DRN projections to any brain region (tectum, hindbrain, etc.), the title must be revised to reflect this.
2. The manuscript structure implies a causal narrative
The paper is organized as a sequential argument: 1) DRN is topographically organized, 2) DRN axons modulate forebrain activity topographically, 3) DRN ablation disrupts forebrain synchrony and sensory-motor responses, 4) DRN ablation amplifies behavioral responses in head-restrained fish, 5) DRN ablation amplifies defensive behaviors in freely swimming fish. A reader following this narrative naturally concludes that the behavioral effects in steps 4–5 arise through the forebrain mechanisms documented in steps 2–3. This inference is reinforced by placing the head-restrained behavioral result within the same figure as the forebrain data, despite these being, accordingly to the authors, logically independent observations.
3. Key text in the Discussion still implies the link
The Discussion states: “We propose that locomotor signals from DRN can be utilized to modulate forebrain activity. Our results revealed that such DRN inputs amplify forebrain responses to sensory cues such as mechanical vibrations, as we observed in our experiments (Figure 6F-H). This can be particularly important during defensive behaviors, where animals make risk-benefit analyses. In fact, we observed that ablating DRN amplify lasting defensive behaviors after a transient threat.” This passage directly links the forebrain modulation to defensive behaviors, which is the claim the authors repeatedly insist they are not making. Although the authors added a caveat in the following paragraph acknowledging that DRN innervates the entire brain, the rhetoric of the passage above substantially outweighs it.

As I mentioned in the original review, there are two disconnected stories presented in the paper. The authors have not attempted to address, or even acknowledge, the problem associated with this.

(Remarks on code availability)

Reviewer #4

(Remarks to the Author)
This revision marks a notable improvement. The authors have thoroughly responded to the primary issue of differentiating between behavioral and stimulus responses, and they have also resolved all my minor concerns.

However, one issue still remains: The micrograph in Fig. 5 shows that resolution does not clearly separate axons from cell bodies, as they are in contact in both xy and likely z dimensions. It may be that axons from the raphe are located on top of the imaged nuclei (of course, not inside). As such, the imaging of these nuclei may contain, in fact, also signals from the raphe axons, potentially inducing spurious correlations. In addition, several axons, including those distant from most segmented axons, are still unsegmented, while some segmented cells resemble axon boutons. In principle, their correlation analysis is a great idea to probe for functional connectivity, but these issues make the claims about raphe axon and forebrain neuron correlations and the general approach not fully convincing. I would recommend that the authors clearly state this limitation in their results as well as address this point in their discussion. If the authors decide to drop this analysis, this would also be OK.

(Remarks on code availability)
I have checked the GitHub repository, and it looks quite minimal. I have not tried to run the code. It would be nice if the authors could expand their readme file a bit to explain the structure of the codebase and how to download the processed data. Documenting MATLAB files will also help readers to understand the analysis.

Reviewer #5

(Remarks to the Author)
I co-reviewed this manuscript with one of the reviewers who provided the listed reports. This is part of the Nature Communications initiative to facilitate training in peer review and to provide appropriate recognition for Early Career Researchers who co-review manuscripts.

(Remarks on code availability)

Open Access This Peer Review File is licensed under a Creative Commons Attribution 4.0 International License, which permits use, sharing, adaptation, distribution and reproduction in any medium or format, as long as you give appropriate credit to the original author(s) and the source, provide a link to the Creative Commons license, and indicate if changes were made.
In cases where reviewers are anonymous, credit should be given to 'Anonymous Referee' and the source.
The images or other third party material in this Peer Review File are included in the article's Creative Commons license, unless indicated otherwise in a credit line to the material. If material is not included in the article's Creative Commons license and your intended use is not permitted by statutory regulation or exceeds the permitted use, you will need to obtain permission directly from the copyright holder.
To view a copy of this license, visit https://creativecommons.org/licenses/by/4.0/

Dear Editors and Reviewers,

We are truly grateful for the reviewers' enthusiastic and thoughtful feedback on our manuscript. It is encouraging to see your appreciation for the novelty and significance of our findings, and we sincerely thank you all for your constructive suggestions, which have helped us enhance the clarity and impact of our work.

After carefully considering all comments, we have revised the manuscript to the best of our ability. Our responses to each comment are detailed below, including explanations of how we addressed them through new experiments, additional analyses, textual clarifications, and the inclusion of several new main and supplemental figures. To facilitate the review process, all changes in the manuscript are highlighted in yellow.

We believe these revisions have substantially strengthened our manuscript, and we are thankful for the reviewers' valuable input.

All reviewer comments are presented below in **red**, and our responses in **black** letters.

We look forward to your feedback on the revised version.

With kind regards,

Emre Yaksi Aytac Kadir Mutlu

REVIEWER COMMENTS

Reviewer #1 (Remarks to the Author):
- Summary -
The paper "Topographically organized dorsal raphe activity modulates forebrain sensory-motor computations and adaptive behaviors" by Mutlu et al., investigates sensory responses and responses to ongoing behavior in the dorsal raphe of zebrafish. The authors combine visual and mechanosensory stimuli as well as recorded spontaneous behavioral responses to investigate the functional and spatial organization of the dorsal raphe nucleus (DRN). The authors find that raphe neurons respond to these simple stimuli, which is perhaps not surprising given the established roles of serotonin in movement vigor and sensory arousal. Using the amenability of their preparation to calcium imaging the authors demonstrate that different neuron response types spatially segregate while correlations of responses of raphe axons in the forebrain with neighboring forebrain neurons suggest a functional influence on forebrain circuits. Chemogenetic ablations of the DRN furthermore underline this functional influence on forebrain activity and implicate DRN neurons in exploratory and

anxiety linked behaviors. The paper is certainly interesting and sheds new light on the organization of the raphe; however, its writing appears rushed which makes it tedious to read in parts and the methods are currently inadequate to judge rigor of the approach. I furthermore have concerns about some of the analyses used and conclusions drawn which must be addressed before publication.

Response 1: We thank the Reviewer 1 for complimenting the novelty and significance of our work. In this extensive revision, we hope that we managed address all comments as explained below.

- Main points -
Issues with response selectivity
The authors make claims about the response selectivity of DRN neurons (e.g. Figure 2), however, I cannot find any data that speak to the relationship of stimuli and behaviors. E.g. what fraction of taps elicits a behavioral response? Has care been taken to isolate sensory stimuli from behavior? It is not at all explained in the methods, how a neural response is labeled as tap vs. light vs. swim. I assume that most taps elicit swims and it is unclear whether such episodes have been removed from the displayed responses. The authors need to show the frequency of behavioral responses to the varied stimuli and discuss whether episodes with behavioral responses were removed from the analysis and if not, why not.
Response 2: We thank for these suggestions. In fact, in our original analysis, we did not want to make strong comments about response selectivity of DRN neurons, but thanks to the reviewers' suggestions, we discovered new insights into DRN activity and its connection to animal behavior, which are now presented in Figure **2, and** Supp Figures **3, 4**.
Firstly, our optimized head-restrained juvenile zebrafish preparation enabled us to monitor animals' locomotor behavior simultaneously to brain activity. Hence, we now disentangle and present animals' DRN responses to spontaneous locomotion, locomotion evoked by mechanical vibrations, mechanical vibrations that do not evoke locomotion, and finally light stimulation, separately (Figure **2, Supp Figure 4**). These results allowed us to better reveal which DRN neurons are selective and which DRN neurons are multi-modal for these different stimuli. Moreover, as asked by the reviewer, we also investigated whether there is any obvious adaptation in these locomotor responses (Supp Figure **3C-D**). We saw only little adaptation in behavioral response, likely due to our long interstimulus interval of 1min.
Secondly, as the reviewer mentioned, we did not initially separate tap induced locomotion from spontaneous locomotion. We took this advice and used only spontaneously generated locomotion when presenting locomotion related responses (Figures **2, 3, 4**). We did not see any significant differences in any of these new results and conclusions, when compared to our earlier conclusions with all locomotor activity. The new results presented in **Figure 2E**, shows clear spontaneous locomotion evoked activity in DRN neurons.
Thirdly, we also took the suggestion of the reviewer and compared selectivity of DRN responses to pairs of locomotion, vibration, and light evoked activity (**Supp Figure 4P-S**). Here, indeed we observed that in some of the DRN neurons vibration induced responses are also comparable to locomotion induced responses (Supp Figure **4Q,**

magenta). Yet, we also find out that there is a specific group of DRN neurons that will respond to locomotor activity and not to vibrations (**Supp Figure 4Q** in brown). Our new results are in line with the idea that DRN neurons have diverse functional features, which has certainly strengthened our initial arguments on the functional diversity of DRN neurons. We are grateful for these suggestions that enable us to better present the functional properties of DRN neurons.

On a related note, the legend of Figure 5 talks about “Tail-beat” responses but the symbols used and the main text suggests that taps were used. Is this because these are one and the same thing (i.e. each tap leads to a tail-beat response)?
Response 3: We thank for noting this; indeed, the presented results were vibration evoked locomotor responses. To be in line with the rest of our study, and to also address reviewers’ comment regarding the analysis of spontaneous locomotion period. We now did a whole new set of experiments, where we analyzed animals’ spontaneous locomotion and presented these new results. Also, we have replaced all wordings of tail-beats to “tail-bouts” for more precise definition in the text. We will use this definition form now on throughout the text. Our results with spontaneous tail-bouts (**now in Figure 5**) are identical with our previous results with vibration evoked tail-bouts (**which is now moved to Supplemental Figure7**). These new data and findings strengthen the relationship between DRN axons in the telencephalon and telencephalic neuron activity. Our conclusions remained unchanged.

Issues with interpretation of correlations
1. The authors find a modest decrease in forebrain activity correlations (both positive and negative) after DRN ablations (Figure 6B-D) as well as a decrease in overall telencephalic responses (Figure 6E-H). Since calcium imaging is limited by shot-noise the former can trivially be explained by the latter. As responses decrease, correlations will naturally decrease significantly even if no circuit properties are altered simply due to the sensitivity of correlations to the signal-to-noise ratio. Significance in the reduction of correlations therefore has to be evaluated compared to losses in correlation in a shot-noise limited model when comparable reductions in peak amplitude are observed. The current significance test is insufficient. Absent this the data should be moved to the supplements and it should be made explicit in the text that the reduction in correlations may be a consequence of the reduction in response amplitude. I don’t think doing this takes away from the paper but will improve rigor.
Response 4: We are thankful for this comment and further suggestions to improve these results that reviewer suggest further down the comments. To investigate the potential caveat about reduced responsiveness of forebrain neurons leading to reduced response correlations between them during sensory response, we did what the reviewer suggested below and randomly selected a number of forebrain neurons with no significant differences in their sensory responses between DRN ablated and control group (**Supp Figure 8A**). We next performed our correlation analysis only on those response intensity matched telencephalic neurons. Despite the matching of their response intensity, the DRN ablated neurons showed a significant reduction between their response correlations across the forebrain and within several forebrain regions (**Supp Figure 8A-D**). We also would like to highlight that a significant reduction in correlated activity across the forebrain in DRN ablated animals are also present during

spontaneous forebrain activity, with no stimulation (**Supp Figure8E-G**). Hence our new analysis and these combined results only strengthen our argument that DRN ablation reduce forebrain synchrony during both sensory responses and during spontaneous activity. We kept our main figure as it is and included these new additional control analysis and experiments in the supplemental figures. We hope that the reviewer will agree on our choice to highlight these findings based on these new supporting results.

2. The authors claim (Page 6) "... whereas inhibited DRN axons displayed stronger negative correlations with neurons in their vicinity (Figure 5H-I)." It is unclear to me how this conclusion was reached. 5H indeed suggests that proximity to axons that respond with excitation to tail-beats (taps?) have higher positive correlations, but 5I essentially shows correlations that are not significantly different from the shuffles, except maybe at distance 0. However, these are still not "stronger negative correlations" considering that they are smaller than most observed excited correlations. I might be misreading here what the authors want to say.
Response 5: We agree with the reviewer that the spatial relationships of negative correlations are weaker. The same point of the reviewer was further supported by our new experiments added to include spontaneous locomotion evoked activity (**in Figure 5**), in addition to vibration evoked tail-bouts (which is now moved to **Supp Figure7K-O**). Hence, we decide to follow the advice, and we only kept the claim about relationship between positive correlations and spatial distance between raphe axons and nearby forebrain neurons. If the reviewer and the editor prefer, we can completely remove this spatial analysis, since it does not make any substantial difference in our main message that DRN axons activity relates to the tail-bout evoked (**Figure 5, Supp Figure 7 A-B**), spontaneous (**Supp Figure7G-J),** and vibration-evoked (**Supp Figure7K-O**) activity of forebrain neurons.

* Issues with region responses *
To identify changes in the relationship of forebrain activity to behavior after DRN ablation the authors are "relating the neural response of each forebrain region to animals' behavioral responses." It is unclear to me what that means. Does this refer to average activity across the entire region? If yes, why was that choice made? In that case, a loss of responsiveness does not have to mean that neurons within the region respond less, but it could simply mean that the balance between excited and inhibited neurons changed. A more appropriate approach would be to perform per-neuron fits of the behavior-activity relationships and report the distributions of slopes between the ablated and non-ablated conditions. In a similar vein, since the authors are effectively interested in the change in slope it is unclear to me why they chose to fit the model with the interaction terms instead of fitting models separately on the ablated and control data. Approaching this per neuron would overcome this issue as well.
Response 6: We are grateful for this suggestion to approach this problem per neurons rather than averaging per brain region. As suggested by the reviewer, we now fitted a line for each forebrain neuron responses and its relation to animals' locomotion response. The slopes of these lines as indicated in updated **Figure 6J**, is the specific relation between a forebrain neuron's activity and animals' locomotion strength in each locomotor response to vibration stimuli. We observed that this relationship was significantly different and closer to zero slope in DRN ablated animals (in red), when

compared to control group (in black) in **Figure 6J**. While we agree that visualization of these lines for thousands of neurons are harder, we agree with the reviewer suggestion that doing this analysis and visualizations per neuron is a better strategy, to disentangle a reduction of responsiveness, versus a change in excitation/inhibition imbalance in telencephalon. We hope that our new analysis and visualizations are agreeable for the reviewer.

* Issues with clarity of methods *
The methods section is currently lacking in detail and clarity, especially with respect to data analysis:
1. The definition of behavioral vs. sensory responses is not given.
2. It is unclear what the text "In Figure 4…" [page 14] means
3. It is unclear what the ICA was used for in Figure 5 and why it was performed. How do independent components chime with the ROI count in Figure 5? What does it mean to be "manually inspected and verified?"
4. There needs to be a clear description on how the different control conditions are generated.

Response 7: We apologize for the issues in clarity of methods. We now tried to update this to better suit the reviewers' requests. Specifically:

1. We tried to better define our sensory stimuli and locomotion behavior in the methods. We also added a frequency spectrum for our mechanical vibrations in **Supp Figure 3A**.
2. Adjust the text regarding identification of axonal pixels.
3. Since these images have both DRN axons and forebrain cell bodies, we had to take a different approach to identify DRN axons. Hence, after manually excluding the regions with telencephalic neuron cell bodies, we adopted a ICA based method for axonal ROI detection from Mukamel, E.A., et al. *Neuron* (2009). Once axonal ROIs are identified with ICA, we did manual/visual inspection of ROIs and confirmed their overlap with DRN axons. The remaining analyses for this figure were done based on those DRN axonal ROIs, corresponding to DRN axonal segments. Hence ICA was only used to identify ROIs, but not while analyzing DRN axonal calcium dynamics. We apologize for the lack of clarity, and we hope that this clarification and textual update help.
4. We apologize for the lack of clear description. These descriptions are now added in the methods, under section entitled "Control conditions of experimental animals".

- Minor points and textual problems -
A seeming lack of proofreading and checking figure/section references made this manuscript frustrating to read at times. I suggest that the authors carefully check the text but I am listing some points that jumped out to me below:

Response 8: We apologize for this; we did a more through proof reading of the text and figure references. We also now went over all these points below and addressed them as suggested. You will find one-by-one replies to these in the below section:

P2: Vglut3/Trh/Gad1/Gad2 are abbreviated on first mention.

Response 9: Full names are given now in the text.

P2 and discussion: I'm not a fan of references to "ancient cortico-limbic" structures or cortex homologs. The cortex has a very specific cellular organization as its defining feature and absent that it's evolutionary origin does not suggest functional similarity.
Response 10: We thank for this thoughtful comment. It is well accepted that te proposed homologs of mammalian cortico-limbic circuits are located along zebrafish forebrain, and we have given several references supporting these claims. Yet, we agree with the reviewer that in the future through molecular investigations of cell-types along zebrafish forebrain along with developmental and functional studies are needed to identify which specific regions corresponding to which cortical structures. We should also note that our use of the term cortex, does not only encompass mammalian "neocortex", but also includes other cortico-limbic structures such as the hippocampus and cortical amygdala. This is also inline with the literature on vertebrate cortical evolution. Hence with support from the reviewer, we hope to keep our statement regarding the proposed homologies between zebrafish forebrain and mammalian cortico-limbic structures, especially given the references that we have provided supporting this argument. While, we think that our argument regarding the role of DRN in modulating forebrain activity does not necessarily require a strong homology between zebrafish and mammalian forebrain, a recent publication (https://peercommunityjournal.org/articles/10.24072/pcjournal.592/) that was presented during our revision, showed locomotion related activity of DRN axons innervating visuomotor/associative areas of the mammalian cortex. Hence these new results in the mammalian cortex are in line with our observations in the zebrafish forebrain.

P3: Unclear what "sensory-motor events" refers to – are these events of coincidence of some sensation and action? Or does it refer to both "sensory or motor related responses".
Response 11: The term "sensory-motor events" was used in one sentence in the introduction section. We now removed it and instead used the term "sensory stimulation and locomotor responses". In the rest of the text "sensory-motor responses" were used to infer "sensory and motor responses". If the reviewer thinks it is necessary, we can replace all "sensory-motor" with "sensory and motor" in the text. We hope to keep the text as it is, with the support from the reviewer.

P3: "that are either inhibited or excited ... or sensory-evoked activity" – what does this mean. If there is "sensory evoked activity" then it is by definition excitation. Or does this refer to sensory-evoked behavior?
Response 12: We now changed this as "DRN inputs that are either inhibited or excited during locomotion or sensory stimulation"

P3: "... were inhibited by each tail-beat" – I do not think a calcium-indicator could resolve this. Do the authors mean "swim" as a collection of tail-beats?
Response 13: We now changed this to "Across all recorded fish (n=12), 27% of DRN neurons were excited, and 38% were inhibited by each locomotor bout". Also all wordings of "tail-beats" in the text is now replaced by "tail-bouts". Thank you for

pointing this.

Page 4 Figure S1 panel references are wrong – they point to the wrong panels and the intended panels appear missing.
Response 14: Thank you for pointing this out. We carefully reviewed the figure and the panel references in the manuscript. We appreciate your attention and have updated **Supp Figure 1** and ensured that all panel references are correct and correspond to the intended panels. The revised figure now accurately reflects the descriptions in the text. We believe this update resolves the concern.

Page 5: "we observed that mechanical vibrations" – but figure legend talks about tail-beats. Figure symbols on the other hand do suggest mechanical vibrations. Which is it?
Response 15: As stated above in ***Response 3*** indeed, the presented results were vibration evoked locomotor response. To be in line with the rest of our study, and to also address reviewers' comment regarding the analysis of spontaneous locomotion period, we now did a whole new set of experiments, where we analyze animals' spontaneous locomotor tail-bouts and presented these new results.

Page 6: "Such recovery requires a level of cognitive…." – this sentence contains a lot of strong words for a test that could rely on simple habituation. That doesn't take away from the experiments performed, but as currently written (e.g., "cognitively demanding") the sentences seem completely disconnected from the experimental tests.
Response 16: We thank for this suggestion. We now altered this section as "It is essential for animals to initiate defensive behaviors upon threats and recover after the threat to explore their surroundings for potential resources. Such recovery requires assessment of the intensity and history of threats, as well as to analyze associated risks and benefits.". We also deleted the sentence "This cognitively demanding and adaptive behaviors are often attributed to cortico-limbic structures in the vertebrate forebrain." Finally, based on the feedback we have received from the reviewer, we also adapt our wording in the text and title from **adaptive behaviors** to **defensive behaviors**, which is more in line with the kind of behavioral test we have performed in DRN ablated animals.

Figure 1H: I don't think the shuffled data adds much to this panel here but instead makes it confusing. To properly evaluate the shuffle result would require a KDE since it is near impossible to judge dot density especially with the overlaid KDE of the non-shuffled data. I suggest removing the shuffles and instead show a KDE of the shuffles (on the same color scale as the true data) in the supplements.
Response 17: We agree with this suggestion, we hence moved the shuffled distributions in the supplemental figures in the form of KDE, as suggested **Supp Figure 1C.**

Figure 2H: This panel is unclear since different colored dots would hide each other so AP and DV cannot be read out at the same time. I suggest a split into two panels. The same is true for other overlays.
Response 18: We now show a split panel for these in **Supp Figure 4C-D,G-H, K-L.**

Figure 5: As mentioned above for C-H there is a disconnect in legend ("tail-beat responses") and symbols used ("vibration stimulus").
Response 19: We corrected this as explained in in ***Response 3.***

Figure 7A: This plot should be normalized to the bottom position reached to ensure that the results in 7C aren't causing the apparent slower recover in 7A. In other words, the statement made for 7A is a statement about slopes and a normalized display would help make that point.
Response 20: Thank you for the suggestion, based on the reviewers' feedback we decided that the term defensive behaviors (rather than adaptive behaviors) better describe the panel of behaviors that we have tested in our DRN ablated animals. Hence, we re-analyzed the vibration evoked defensive behaviors and their lasting effects after transient exposure to stimuli, in our updated figures. Firstly, we tested how animals' defensive behaviors evolve over time, by plotting the distance of animals from the bottom of the tank, and statistical differences (p-values) between DRN ablated and control groups as a function of time (sliding 5second bins) during 50 seconds window after vibration (**Figure 7A**). This plot clearly shows that the impact of vibration stimulation on animals' swim depth is quick and lasting. We also tested the idea of the reviewer by normalizing the swim depth data to animals' bottom position to test potential differences in recovery (**Supp Figure 9A**). This plot showed small/no effects in recovery of DRN ablated animals. Next, we tried to fit exponentials to animal behavior to potentially address whether there is a faster recovery as suggested by the reviewer. However, due to large variability in animals' behavioral responses to individual vibration stimuli, this approach did not yield any relevant statistics to make a strong argument about recovery period. Hence, we choose not to make major claims about the slope of recovery during these defensive behaviors. These time-constant calculations are now presented in **Supp Figure 9B**. If preferred by the reviewer, we can completely remove this recovery time-constant calculations from the manuscript.

Figure 7J: What is the N in this figure and what are the statistics calculated across?
Response 21: Since there was no Figure 7J, we assumed this comment was made for Figure 6J. We now completely changed the data display in Figure 6J, to follow this reviewer's suggestion and instead plot the slope of every individual neurons' activity as a function of its relation to animal behavior. So in this new plot N is the number of recorded neurons (# neurons in sham: Hb = 1698, Dl = 2590, Dm = 1241, Dc = 1575, Dd = 399, Dmp = 372; # neurons in ablated: Hb = 2158, Dl = 3121, Dm = 1278, Dc = 1795, Dd = 533, Dmp = 344.). We have 16 control fish and 16 DRN ablated fish for this figure.

Reviewer #1 (Remarks on code availability):
URL was not reachable but simply led to a 404 error. This is unfortunately in line with my general impression of the paper being rushed and not properly proofread.
Response 22: We apologize that the codes were in the link, but the link was not made public. We now enable public sharing of the codes.

Reviewer #2 (Remarks to the Author):

This is a very nice study that identifies topographic features of the zebrafish dorsal raphe during baseline and in specific behavioral contexts, and relates these features to neuronal activity in the forebrain. The experiments are clearly described and presented, and are interpreted in a reasonable manner. The experiments yield clear and interesting answers to the questions posed. Overall, this is a solid contribution to what is rapidly becoming a highly studied area of neuroscience. I have only minor suggestions for improvement, outlined below:
Response 23: We are grateful for the supportive comments on the value of our findings and its presentation. In this revised manuscript we now further answered all pending points with new analysis, new experiments, and textual changes.

1. Page 3: Please fix this error on page 3: (Error! Reference source not found.A, B)
Response 24: We apologize for this formatting issue. This is now fixed.

2. Figure S1C,D: I'm not sure what this is showing. Does % of excited neurons represent the number of neurons that show excitation divided by the total number of raphe neurons that were imaged (i.e. 80 neurons)?
Response 25: Yes, this figure indicates the fraction of DRN neurons and their spatial distributions (anterior-posterior and dorsal-ventral) that are excited or inhibited up on locomotor activity or sensory stimulation. These fractions are calculated in each fish separately and their mean+/-SEM is plotted in this figure. We hope this clarifies this point.

3. Fig 3A,B: the authors state that ~25% of DRN neurons are Gad1b positive, but in the images shown it look like most of the DRN neurons are Gad1b positive. Is this because the images only show focal planes where there is a high level of co-expression? If this is the case, it would be better to show images of all DRNs to better convey the conclusion that only 25% of cells are co-labeled.
Response 26: We originally used confocal microscopy images, which are higher resolution then two-photon microscopy images, to highlight the fraction of tph2:Gal4 DRN neurons express Gad1b. But indeed, our selected image doesn't fully represent the statistics we observed and displayed in our two-photon functional DRN recordings. To address this comment better, we now displayed our two-photon microscopy images (generated during our functional calcium imaging experiments) of a dorsal DRN and a ventral DRN plane. As we indicated earlier this image better reflects the anterior dorsal distribution of Gad1b positive DRN neurons. You will also notice that tph2:Gal4 labelled DRN neurons are surrounded by many Gad1b positive neurons, yet only few of those Gad1b neurons are tph2:Gal4 labelled DRN neurons that we have investigated during this study. Also to answer this comment, we have now plotted Gad1b:dsRed expression levels of all tph2:Gal4 labelled DRN neurons that we have detected and imaged in this study **(Figure 3C)**, normalized in each animal by using z-scores. Here we marked the threshold that we have used in our study that was selected to identify which tph2:gal4 labelled DRN neurons with clear expression of Gad1b:dsRed. All neurons above this threshold were considered Gad1b positive and comprise just above 25% of tph2:Gal4

labeled DRN neurons. We hope that providing this distribution plot enable the reviewer to assess our process of identifying Gad1b expression in DRN neurons. We have also performed our analysis based on small variations of this threshold, these iterations did not yield any differences in our findings and conclusions.

4. Fig 3F,J,M,P: I think it would be useful to also show color-coded clusters for only the Gad1b+ DRNs, which would give spatial information about Gad1b+ clusters that is not provided by the graphs shown in panels K,N,Q.
Response 27: We now presented our results as suggested in **Figure S5**

5. Fig 4. The 2D histograms are a nice way to convey the spatial information. It would be interesting to see a comparison between the different behavioral contexts (i.e. tail movement, response to light, response to vibration) and the location of excited and inhibited fibers in the forebrain. For example, The inhibited fibers for tail movement and vibration stimulus look grossly similar, but can this be quantified?
Response 28: We thank for these suggestions. Indeed, these 2D histograms very useful to identify locations of DRN axonal activity. Yet, we also agree that some of these histograms look rather similar. We quantify differences of these histograms between light versus vibration stimuli versus locomotion responses (**Figure S6**). Indeed, these differences very well highlighted the specific forebrain regions that receive functionally distinct DRN axonal innervations. Moreover, inspired by another reviewer's comment, we also provided similar 2D histogram along the sagittal views (**Figure 4J-R-Z, Figure S6C-F-I**). These graphs clearly showed a spatial segregation of DRN axons based on their functional properties along dorsal-ventral axis of the sagittal view. We thank for this suggestion, and we hope that our new figures and analysis fully address this comment.

6. Fig 5C,D: The image appears to show vibration stimulus data, as stated in the main text, but the figure legend states this is tail-beat data.
Response 29: We thank for noting this; indeed, the presented results were vibration evoked locomotor response. To be in line with the rest of our study, and to also address another reviewers' comment regarding the analysis of spontaneous locomotion period. We now did a new set of experiments, where we analyzed animals' spontaneous locomotor tail-bouts and presented these new results. Our results with spontaneous locomotion (**now in Figure 5**) are very similar to our previous results with vibration evoked locomotion (**which is now moved to Supp Figure7K-O**). These new data and findings strengthen the relationship between DRN axons in the telencephalon and telencephalic neuron activity.

7. Fig 5E-I: it would be interesting to know if the forebrain inhibited neurons are more spatially correlated (and if forebrain excited neurons are anti-correlated) with nearby Gad1b positive DRN axons.
Response 30: Indeed, this is a very good suggestion, and we now have tested this. We observed that excited forebrain neurons are more correlated with excited DRN axons **Figure S7A&E,** and inhibited forebrain neurons are more correlated with inhibited DRN axons **Figure S7B&F.** We also calculated the distance between excited forebrain neurons and excited DRN axons, and compared this distance to inhibited DRN axons.

Similarly, we calculated the distance between inhibited forebrain neurons and excited DRN axons, and compared this distance to inhibited DRN axons. These results are presented now in **Supp Figure7A-F.**

8. Fig 6D: While the differences in correlation for sham vs. ablated are statistically significant, the differences appear to be very small. I suggest that the authors note this, or describe why they think the changes are large enough to be meaningful.
Response 31: We agree the effect is small but indeed significant. We now stated this effect specifically as "small but significant reduction in correlations" in the main text. Moreover, upon suggestion of reviewer 1 we also did a series of new analysis to ensure the stronger effects on response correlations that we observe hold under different conditions. Please see our **Response #4 (above)** for detailed explanation of these additional controls and analysis ensuring that the correlation effects that we observed remains significant under multiple conditions.

9. Fig 6J: While the differences are significant, they appear to be driven by a very small number of outlier data points. Do the authors agree with my interpretation? If they agree, I would soften the language of the text to reflect this.
Response 32: We agree that our effect is small but significant across many forebrain regions and we updated our results accordingly in the text. Moreover reviewer 1 suggested an excellent analysis to improve these plots, not only region by region but neuron by neuron, hence we now present the slope of the relationship between the activity of individual neurons and animals locomotor behavior in this new updated figure. While our results remain unchanged, up on this new and more fine-grained analysis, differences between control and DRN ablated groups are prominently visualized. A detailed explanation of these new results can also be found in **Response #6 (above).** Also, we now soften the language by stating this effect specifically as "small but significant reduction in correlations" in the main text.

10. Fig S2A: again, the differences are significant but apparently very small, so it's unclear how meaningful the differences are.
Response 33: We thank for this point, which was addressed above in our **Response 31 and Response 4 (both above)**.

Reviewer #3 (Remarks to the Author):

The study captures interesting functional diversity in the DRN population in response to mechanical and visual stimuli. The results regarding the functional and anatomical diversity of DRN are convincing and valuable to the community.
The effects of DRN ablation on behavior (e.g. increase in sensory-motor response) are also credible. However, these results lack clarity in the presentation and, more importantly, are overstated to imply that the behavioral effects are due to DRN-forebrain interactions.
The most unconvincing claims concern the behavioral consequences of DRN-forebrain interactions. Overall, the latter half of the results sections presents a number of

conclusions that are not supported by sufficient evidence.
In summary, the manuscript consists of three main parts – DRN diversity, behavioral phenotypes following DRN ablations, and forebrain sensory-motor activity changes in response to DRN ablations. However, the relationship between these three parts is unclear. At times, the authors seem to want to imply that there is a coherent narrative by simply putting results next to each other in a figure (e.g. Figure 6 and comment #5 below). However, to actually support these claims, deeper analyses and significant additional controls are necessary.
Finally, there are too many instances where basic experimental and statistical details (e.g. standard deviations, duration of time windows, animal numbers, meaning of lines on plots, etc.) are missing.
Response 34: We thank for highlighting the value of our work, and we also appreciate the feedback regarding the presentation of our findings and their implications. Indeed, our findings on the functional diversity of DRN neurons and their diverse projections to the forebrain is, to our knowledge, the first of its kind. To our knowledge, such modulation of forebrain sensory and motor representations, after DRN ablation was also not shown before. Finally, impact of DRN ablation on animals' sensory evoked and defensive behaviors is well supported by our results. After reading the comment of Reviewer #3, we have carefully read through our manuscript, and we could not find any section, where we claim that the behavioral effects that we observed after DRN ablations are only due to DRN forebrain interactions. We also did not intend to do such claims in the text, and we apologize for this misunderstanding. We went over the text again to make sure that our findings are better explained.
Unfortunately, specific ablations of DRN to forebrain connections while performing behavioral tests in free-swimming animals, is not experimentally feasible. There is no transgenic line that exclusively label forebrain projecting DRN neurons. Neither there is any evidence for zebrafish DRN neurons that are specifically targeting forebrain but no other brain regions. Hence testing the specific impact of DRN to forebrain connections in free-swimming behaviors in zebrafish is not a technically feasible experiment. Given that we did not make such a claim, we hope that reviewer #3 will agree that proving a claim that we did not make (on specific impact DRN to forebrain connections in animal behavior) is not within the scope of this study.
Finally, you will notice that we did our best in answering all other points raised by the reviewers. We hope these revisions improve the clarity and transparency of our work and demonstrate our commitment to addressing all feasible concerns raised by the reviewers. We hope that, given these clarifications and improvements, the manuscript is suitable for publication.

Major comments:
1. The manuscript strongly implies that changes in "DRN-forebrain interactions" are responsible for the behavioral results in Figure 7. In the set-up for the experiments in Figure 7, the authors state that "This cognitively demanding and adaptive behaviors are often attributed to cortico-limbic structures in the vertebrate forebrain". However, there is no evidence that the effect of DRN ablation on diving behavior has anything to do with the forebrain. Figure 7 shows a real effect of DRN ablations -- increased diving depth in response to stimuli. However, this could have been generated entirely independently of changes in forebrain activity. The DRN projects broadly throughout the

brain. Other brain regions (e.g. optic tectum, hindbrain) could be the source of the observed behavioral change.
The only way to potentially support the claim that DRN-forebrain interaction has a causal role in modulating diving behavior is to demonstrate that forebrain ablations uniquely alter the diving depth, or better still, specifically ablate the DRN projection to the forebrain. Unless either of these experiments can be performed, the claim about DRN-forebrain interactions in the context of the diving behavior is entirely speculative.
Response 35: As explained above we did not intend to make any claim that "DRN-forebrain interactions" are responsible for the behavioral results in Figure 7. We also did not find this claim anywhere across our manuscript. We apologize if the sentence "This cognitively demanding and adaptive behaviors are often attributed to cortico-limbic structures in the vertebrate forebrain", implied such a claim. We now removed this sentence from the results section to avoid this potential confusion. As the reviewer pointed our DRN projects broadly across the brain, and there is no evidence for the presence of DRN neurons that are only projecting to the forebrain. Hence specific ablation of DRN projections to forebrain is not technically feasible during such free-swimming behaviors, with our current technologies.

2. The usage of the term "adaptive behavior" throughout results related to Figure 7 is odd. "Adaptive" in what sense? The DRN ablated animal dove to a great depth (consistent with the strong tap responses shown in Figure 6i). In this case, it would of course take the animal longer to re-surface. The post-ablation plot in Figure 7a looks like the control plot plus a constant. The time window chosen for the analysis seems arbitrary.
Response 36: We agree with the reviewer on the use of term "adaptive behavior" was not a good choice. And perhaps this was the source of misunderstanding that we have tried to discuss above. Taking this feedback in to account we believe that "defensive behaviors" better encompasses the tests we have performed and the claims we make for this study. We also now choose to indicate the statistical difference in animal behavior as a function of time, by plotting the changing p-values across 50 seconds (**Figure 7A**). As evident by these new statistics, DRN ablated animals' defensive responses to transient mechanical vibrations are statistically different in across multiple time windows. We hope that this way of plotting the data and statistics better displays relevant time window for assessing animal behavior.

3. The most credible takeaway is that DRN ablation leads to greater behavioral responses to mechanical stimuli. Introduction to a novel chamber would also be associated with sudden mechanical, optical, and/or flow-associated stimuli, assuming the animal is dropped into the chamber by the experimenter. The current presentation of the results obscures what appears to be a simple point.
Response 37: We thank for pointing out this. As explained above, choosing the term "defensive behaviors" better encompasses both vibration responses, and novel-tank diving responses that we have tested in DRN ablated zebrafish. Both vibration responses and novel-tank diving responses are relatively long lasting for up to tens of seconds after animals' experience of the stimuli. Moreover, the statistical differences in vibration responses appear prominent at later time windows, long after the vibration stimuli is over. We agree that this is a defensive behavior, and we hope that the textual

changes that we made in this results section now better communicates our arguments and avoid confusions regarding our claims.

4. One can make a simple objection to the conclusions in Figure 6 – if the sensory-motor responses in the forebrain are weaker following DRN ablation, then the SNR of the cells are lower. More specifically, the noise floor of each neuron (e.g. due to shot noise) is unlikely to be affected by DRN ablation, but the real signal is now significantly lower in amplitude. Mathematically, this directly leads to decreased correlation between cells and has no bearing on whether the actual "synchrony" has changed. Given the relatively modest change in Figure 6, one would need to be convinced that the effect is not simply due to changes in SNR. For example, one can sub-sample a set of cells with identical response amplitudes to stimuli pre- and post-ablation and demonstrate that these cells show loss in synchrony. If this isn't the case, then the authors have simply shown that forebrain neurons have weaker sensory-motor responses, which is credible. Again, as in Figure 7, it seems that there is a real result in Figure 6, but one that has been unconvincingly overstated.
Response 38: We thank the reviewer #3 for this suggestion regarding subsampling of forebrain neurons with similar responses and testing the effect of DRN ablation on their synchrony. We now did this test and presented our results in **Supp Figure 8A-D.** Indeed, even after subsampling neurons with statistically similar responses, we still observed that within and across forebrain region correlations are significantly reduced after DRN ablation. We hope that our new controls make a more convincing point about the reduction in forebrain synchrony in DRN ablated animals. We now updated our results section accordingly.

5. Figure 6i has the same issue as Figure 7. There is no supporting evidence for the claim that the decrease in forebrain sensory-motor response is responsible for a significant increase in motor responses. Again, to demonstrate that this is true, the authors would need to specifically ablate the DRN projection to the forebrain or at a minimum, demonstrate that forebrain ablations lead to increased sensory-motor responses.
By showing this result into a figure primarily on forebrain responses, there is some implied suggestion that these observations are related to each other. However, unless the above control experiments are performed, there is no reason to expect that a real relationship actually exists.

Response 39: We did not make a claim "that the decrease in forebrain sensory-motor response is responsible for a significant increase in motor responses", anywhere in our text. Our preparation enabled us to investigate a limited aspect of animals locomotory response to mechanical vibration while imaging forebrain activity, and we reported our observation that DRN ablated animals have amplified vibration responses in head-restrained zebrafish. This is somewhat in line with our observations in **Figure 7** that DRN ablation animals have increased defensive behaviors during free-swimming. Moreover, our new analysis requested by other reviewers, now presented in **Figure 6J** shows that the relationship between forebrain activity and animals locomotor responses are reduced in DRN ablated animals. These results do not imply that DRN to forebrain connections are the underlying cause of behavioral alterations, but instead DRN

ablated animals showed impaired the relationship between the forebrain activity and animals' locomotor responses to forebrain. Hence, as we discussed in our discussion section, perhaps DRN projections to forebrain might be one of the several channels that can communicate animals' locomotor activity to the forebrain. We hope that the reviewer agrees that this is a reasonable conclusion based on our results.

6. It's unclear what is the underlying phenomenon that drives the correlation patterns observed in Figure 5, or what is the significance of the result in 5h. The DRN axon primarily innervates a central neuropil region. For instance, let's assume that the forebrain brain neurons activated by taps are simply distributed around the neuropil region. Geometrically, if one mirrors this layout across the left-right axis, then one would trivially expect that the correlation will be distance-dependent. It's unclear whether this reveals any relationship between DRN and forebrain neurons.
To show that there really is something non-trivial, the distance correlation should be done on one half of the forebrain (left or right) at a time. Furthermore, one would ideally like to have some clarity as to why and how the correlations would be stronger or weaker.
Response 40: The correlations as a function distance presented in this figure were indeed calculated within forebrain hemispheres and later results are merged for displaying this across all fish. We agree that our observations are purely based on correlations and not causal. Yet our general observation of correlation between DRN axons and forebrain neurons are also in line with an earlier publication that showed that optogenetic activation of DRN, generate bursting activity in a subpopulation of forebrain neurons (Figure 5B-D in https://doi.org/10.1038/s41467-024-47021-0), which we have already cited in the discussion section of our manuscript. We believe that our correlation-based analysis adds an additional dimension for the relationship between DRN axons and forebrain activity, and hence we prefer to keep these results. If the reviewer thinks that our correlation-based results are of limited value and do not add to our main message, we will be happy to discuss further with the editor and reviewers, whether or not to remove these results from our manuscript. If permitted, we prefer to keep these calculations and results as they are in line with previous literature.

7. Claims about modality-specific distribution of functional responses in the DRN need more quantification or qualification. For instance, the authors state that "increased fraction vibration-evoked inhibitory responses observed towards the anterior-dorsal DRN". However, this may be influenced by a variety of factors including stimuli strength and habituation. Mechanical and light responses adapt over time and adapt at different rates. How are these factors accounted for? How is the stimulus intensity chosen? Parametrically varying stimuli intensity would have been helpful, otherwise one needs to explicitly state that light at x intensity and tap at x intensity elicited the observed results, with justification for the levels chosen.
Response 41: The stimulus intensity were chosen based on several of our earlier studies (https://doi.org/10.1038/s41467-025-62115-z , https://doi.org/10.1016/j.cub.2021.08.021 , https://doi.org/10.1126/sciadv.aaz3173). In brief, our idea is to use two different stimulus modality that we know to elicit neural responses in the forebrain and yet have different behavioral response. The red-light flashes as we used did not elicit locomotor behaviors, whereas mechanical vibrations

reliably elicit such behaviors (**Supp Figure 3B)**. We now also presented how animal behavior changes up on repeated applications of the stimuli in **Supp Figure 3C-D**. To reduce concerns about potential adaptation, we used 1-minute inter-stimulus intervals. Such interstimulus interval is longer than many comparable studies in the field that used these stimulus modalities. We now better clarified the stimulus parameters in the updated text and with an additional **Supp Figure 3A**. Finally, we have now presented better quantification for the modality specific responses of DRN neurons in **Supp Figure 4P-R**. We thank the reviewer for these suggestions.

8. Just as stimulus strengths are unstated in the results section, a number of other experimental details are left out. For instance, the authors state that "Next, we assessed the stability of DRN clusters by quantifying the likelihood that pairs of DRN neurons remain in the same cluster during two consecutive time periods", without actually specifying the time period. This can have consequences for the clustering analysis.

Response 42: Cluster fidelity was calculated by measuring the probability of pairs of neurons being in the same cluster during two consecutive **1.5 minute** periods, as stated in the methods section. We now updated the statement from "different time windows" to "consecutive time windows" for better clarity in the main text. We hope this clarifies this point. Moreover, intrigued by this comment, we also performed longer recordings of ongoing DRN activity, and measured the stability of cluster fidelity during **1hr**, our new results presented **in Supp Figure 1D**, show that functional DRN ensembles are stable over longer periods of time

9. To what extent is the "ongoing" activity of DRN in Figure 1 a function of spontaneous locomotor activity in the animal? If that's the case, then this figure doesn't offer additional information that isn't already covered in Figure 2.

Response 43: We thank the reviewer #3 for this question on the relationship between ongoing DRN activity and animals' locomotion, which enabled us better support some of our arguments, and add a new dimension to our manuscript. We agree that a portion of DRN ongoing activity is influenced by animals' locomotor activity. But we also argue that only a small portion of ongoing DRN activity can be explained by animals' locomotion and DRN ensembles are distinguishable even during periods without locomotion. In fact, to better assess this relationship we have performed new experiments and multiple additional analysis. Firstly, we asked what fraction of variance of DRN neuron activity can be explained by animals' locomotor activity. As expected, the variance of activity in few DRN neurons can be explained above 60% by the locomotion (**Supp Figure 2A**). Yet, in many DRN neurons this was not the case. In fact, on average we found only around 11% of DRN activity can be explained by locomotion (**Supp Figure 2A**). These results state that ongoing DRN activity only partially depends on animals' locomotion. We now added these new experiments and analysis in our updated figure (**Supp Figure 2A**) and the main text. Next, we performed additional longer recordings of DRN activity, during which we can find periods of relative quiescence (no locomotion for at least for ~30 seconds) in addition to locomotion periods. Here we compared the total number of ongoing activity bursts in DRN neurons during transition from a locomotory active period to a locomotory quiescent period. We observed that there is no statistically significant reduction of ongoing activity (**Supp**

Figure 2B-C), despite small reduction of DRN activity in some animals. Finally, we investigated the ongoing activity of DRN ensembles solely during these quiescence periods without locomotion. These new experiments and analysis showed that DRN neurons are sufficiently active, and one can effectively identify DRN ensembles based on their ongoing activity, during locomotory quiescence period (**Supp Figure 2D-I**). Altogether, our results showed that while an important fraction of DRN neurons are excited/inhibited by locomotion, ongoing DRN activity is not solely driven by locomotion. In fact, DRN ensembles are prominent even in the absence of any locomotion, during quiescence periods. We choose to preserve the flow on our manuscript and keep the presentation of ongoing DRN activity in Figure 1. Moreover, we now choose to introduce these new results on the relationship between locomotion and ongoing activity of DRN ensembles (in **Supp Figure 2**), only after introducing the locomotion responses in DRN, presented in **Figure 2**. We think that this is important to keep the flow of our manuscript and our message. We hope that the reviewer and the editors would agree on our choice to keep our presentation flow.

10. Are there conjunctive coding of different stimuli (e.g. Figure 2). Neurons are classified as excited/inhibited/other in response to each stimulus. But there can be neurons with responses to both.

Response 44: We thank for the question. We now present the selectivity of DRN responses to pairs of locomotion, vibration, and light evoked activity (**Supp Figure 4P-R**). Here, indeed we observed conjunctive neurons, as well as DRN neurons with more selective responses. These results are in line with the idea that DRN neurons have diverse functional features, which has certainly strengthened our initial arguments on the functional DRN diversity. We are grateful for these suggestions that enable us to better present the functional properties of DRN neurons.

Minor comments:

1. For all statements on the percentage of neurons (e.g. “Across all recorded fish (n=12) 26% of DRN neurons were excited, ..”), it would be good to include mean and standard deviation across animals.

Response 45: Done.

2. Unclear what is the definition of “baseline” for delta F/F calculations. Neural activity is generally shown as delta F/F, i.e. fractional change in fluorescence relative to “baseline”. In different parts of the Methods section “baseline” is described as 3 minutes, 30 minutes, or as “5s period before stimulus onset”. It is unclear during which period exactly the fluorescence baseline is established, and why such a range was used.

Response 46: We apologize for the textual unclarity in the methods section saying, “baseline recording (30 min)”. This was not a baseline for any calculations. This sentence is now corrected as “After a 30-minute recording without any external stimuli”. Moreover, since inter stimulus intervals were 1 minute and due to high level of ongoing DRN activity, we had to use a shorter baseline of 5 seconds, while calculating the sensory responses. These specific baselines are now better clarified in the text.

3. Time window information is repeatedly missing. In Figure 1, it appears that the pairwise correlation is calculated over the entire window of spontaneous activity (several minutes?), whereas in Figure 2 the pairwise correlation is calculated during "tail-beat responses", so on a trial basis over a couple of seconds (?). This is basic information that should be stated clearly.
Response 47: We now added this information.

4. In Figure 4, it is unclear why there are two vertical dashed lines in the plots. If they represent the stimulus on- and offset, one expects this to be clearly stated in a standard figure legend.
Response 48: We now added this in the legends.

5. What is the animal number for Figure 6j? For panel b, the figure legend specifies n = 16. But there are clearly more than 16 points in 6j, and the animal number is not given in the figure legend for 6j.
Response 49: Up on request from reviewer #1, this analysis is now redone based on individual neurons and sample size are updated in the text. Please also see **Response #6** above regarding why these analyses were remade, based on reviewers' request.

6. How is the average response defined in Figure 6j? Each point represents an individual fish with several stimulations. It is not clear how the "average of average responses" is defined here. As average value of the averaged response until offset of stimulus or until decay of the neural/behavioral response?
Response 50: This analysis is now remade based on individual neurons and sample size are updated in the text. Each trial's neural and locomotory responses are calculated individually. Please also see **Response #6** above regarding why these analyses were remade, based on reviewers' request.

7. It is not clear how exactly the "shuffled/simulated data with same mean and variance" is created.
Response 51: We thank the reviewer for pointing this out. The shuffled/simulated data were generated by randomly permuting the time series of each neuron independently, while keeping the same mean, variance, and distribution as the original data. This procedure destroys the temporal structure and correlations between neurons while preserving basic statistics. We now added explanations about this in the methods section. Also, we should note that we have tried another simulated data, by shuffling time points between different neurons. Also with this alternative shuffling, the results of elbow method suggested 4-5 optimal k-means clusters for juvenile zebrafish DRN neurons labelled by *tph2:Gal4* transgenics.

8. In Figure 5 C and D the text speaks of tail-beat responses, but the image shows the mechanical vibration icon.
Response 52: We thank for noting this; indeed, the presented results were vibration evoked locomotor response. To be in line with the rest of our study, and to also address

reviewers' comment regarding the analysis of spontaneous locomotion period. We now did a whole new set of experiments, where we analyze animals' spontaneous locomotor tail-bouts and presented these new results. Our results and conclusions with spontaneous locomotion (**now in updated Figure 5**) are in line with our previous results with vibration evoked locomotion (**which is now moved to Supplemental Figure7K-O**). These new data and findings strengthen the relationship between raphe axons in the telencephalon and telencephalic neuron activity.

9. Are there difference in baseline tank depth preference (Figure 7)? Ablated fish seem to prefer a deeper tank depth prior to any stimulus delivery based on Figure 7A.
Response 53: In our revised figure, we now present the statistical significance between control and DRN ablated group, as a function of time including the baseline period. As this new analysis show, control and DRN ablated animals do not have significant difference during baseline period, and this difference become prominent and lasts several seconds after the transient stimulus delivery, marked by the vertical dashed line **Figure 7A**.

10. typos: - (Error! Reference source not found.A, B).
Response 54: Corrected.

11. No line numbers
Response 55: Line numbers are now added to the revised text.

Reviewer #4 (Remarks to the Author):

In this study, Mutlu et al. use a combination of functional imaging, molecular circuit analyses, and behavioral experiments to generate a comprehensive description of the raphe in juvenile zebrafish. They find that the raphe nuclei consist of distinct functional and molecular subtypes. They also show that behavior/stimulus excited and inhibited raphe axons have different spatial distributions in the forebrain. Furthermore, they demonstrate that ablation of serotonergic neurons in the raphe decreases sensorimotor responses in the forebrain. Finally, this study detects clear behavioral effects induced by serotonin manipulation. In sum, this study provides new insights into the functions of serotonin, extending previous research on the raphe. Some of the findings may also require additional validation to address potential artifacts of the experimentation.

Before I can recommend publication, it would be important that the authors address the following points:
Response 56: Thank you for pointing out the new insight our investigation brings.

Main points:
- Please clarify whether the animal was moving or not in response to the visual and tactile stimuli. This is important to separate motor, sensory, and sensorimotor effects (for example, Fig. 2J,O). A lot of the described stimulus-response activity could, in fact,

just be related to movement. Similarly, it would be interesting to know if there are any stimulus-specific responses in the raphe that occur when animals do not move.

Response 57: Thank you for these suggestions we now did multiple new analysis to address these comments. We present animals locomotory responses to vibration and visual stimuli in head-restrained zebrafish (**Supp Figure 3B**) in the revised manuscript. We also present responses of DRN neurons to pairs of multiple stimuli, which revealed some DRN neurons can respond to more then one stimulus, and some are more selective (**Supp Figure 4P-R**), highlighting heterogeneity among DRN neurons. Finally, as requested, we compared the DRN responses to vibrations, with and without locomotion (**Supp Figure 4S**). This analysis showed that especially inhibitory responses of DRN neurons are substantially different in trials with and without locomotory responses, strengthening our argument that DRN is composed functionally distinct groups of neurons.

On a parallel note, we now did substantial additional work to disentangle how much of the DRN activity can be explained by locomotion. Firstly, we asked what percent variance in DRN neuron activity can be explained by animals' locomotor activity. As expected, the variance of activity in some DRN neurons can be explained above 60% by the locomotion. Yet, in many DRN neurons this was not the case. In fact, on average we found only around 11% of DRN activity can be explained by locomotion. These results states that ongoing DRN activity only partially depends on animals' locomotion. We now added these new experiments and analysis in our updated figure (**Supp Figure 2A**). Next, we performed additional longer recordings of DRN activity, during which we can find periods of relative quiescence (no locomotion for at least for ~**30 seconds**) in addition to locomotion periods. Here we compared the total number of ongoing activity bursts in DRN neurons during transition from a locomotory active period to a locomotory quiescent period. We observed that there is no statistically significant change of ongoing activity (**Supp Figure 2B-C**), despite small reduction of DRN activity in some animals. Finally, we investigated the ongoing activity of DRN ensembles solely during this quiescence period without locomotion (**Supp Figure 2D-I**). These new experiments and analysis showed that DRN neurons are sufficiently active, and one can effectively identify DRN ensembles based on their ongoing activity, during locomotory quiescence period (**Supp Figure 2D-I**). All together our results showed that functional DRN ensembles are prominent and active, even in the absence of any locomotion.

- Additional details on how the experiments were performed would also be helpful. For all figures, please clarify what was done volumetrically and what plane by plane. Are volumes always acquired dorsally to ventrally? Are planes always scanned in the same direction? How are the behavior and stimuli aligned to the imaging data? At which time during the scanning process? This information would help interpret the short-time-scale effects.

Response 58: We now did our best to clarify this better in the revised methods. All recordings were done volumetrically (except in **Figure 6**, single plane recordings). Scans were always performed using same parameters of scanning and from dorsal to ventral. Behavior, stimulation and imaging data are aligned using a frame-trigger pulse generated by the microscope to control all other recording and stimulation devices. As an additional check-point, we also generated a microscope frame-triggered dark flash

(by briefly turning off infra-red LED used for locomotion imaging) at the start, and end of the recording session to be recorded by our locomotion recording camera. All these ensured that our synchronization was proper across devices and recordings.

- The main point of Fig. 5 is that raphe axons are correlated with nuclear-tagged cell activity in the forebrain—which is a great analysis. However, there could be small pieces of axons in the cell's ROI that could be responsible for the correlation. Showing un-thresholded components could help get a sense of whether this is a problem. Clarifying the xyz size of the voxels would also be helpful.

Response 59: We used a template matching algorithm to identify the nuclei of individual forebrain neurons, expressing GCamp6s-nuclear. Hence, only the neuronal nuclei were detected as neuronal ROIs for these experiments. We do not believe that there is any reason for DRN axons to penetrate inside the nuclei of individual forebrain neurons. This was precisely the reason why we chose GCamp6s-nuclear for these experiments rather than cytosolic GCamp6s. Hence, we never observed any small piece of DRN axons within forebrain nuclei. We cannot think of a scenario where small pieces of DRN axons will find themselves within pixels corresponding to neuronal nuclei. Finally, we observed substantial correlations between DRN axons and those forebrain neurons that are far from the locations of DRN axons (**Figure 5E**), which cannot be explained by interference from small pieces of DRN axons near neuronal nuclei. We believe that our results and arguments are within the reasonable spatial resolution limits of two-photon microscopy recordings (enabling detection of axons), if not higher than many of comparable studies. We hope that the reviewer would agree on this matter. If not, we would also be happy to drop these experiments, which are not key to our main message. Nevertheless, we are confident on the quality of our experiments, and we prefer to keep these results if the reviewer would agree.

Clarifying the xyz size of the voxels would also be helpful.

Response 60: Axonal data in this figure was based on identifying DRN axonal ROIs using an independent component analysis of covarying axonal pixels. Hence there are no voxels used in this analysis but instead axonal ROIs. ROI identification was important for reducing the noise in axonal pixels, since we had fewer axonal fragments during these experiments, due to joint nuclear imaging of forebrain neurons and DRN axons in the forebrain.

Minor points:

Figure 1:

- Fig 1B: please present non-saturated micrographs if possible.

Response 61: We updated accordingly.

- Fig 1C,D: is this from plane or volume recordings? This is important to interpret whether these are the total number of serotonin neurons in each fish.

Response 62: These are volume recordings.

- Since the reporter is cytosolic, it would be good to demonstrate the quality of the segmentation.

Response 63: We updated the figures to better show the segmentation quality.

- Fig 1D: Was the fish tail free? What percentage of the variance is explained by the behavior? —this information is also not in Fig. 2 but is important for the claim "We discovered that a large fraction of DRN neurons is primarily driven by animal locomotor activity" from the abstract.
Response 64: Yes, in all our experiments we freed animals' tail. The variance explained is now plotted in **Supp Figure 2A**. Please also see our longer response to this question, in **Response 57**. Finally, we updated this particular text in abstract as "We discovered that a fraction of DRN neurons are driven by animals' locomotor activity".

- Fig 1H: Cluster stability was tested only on short time scales (consecutive time periods); it would be more meaningful to test stability over longer time scales.
Response 65: This is a very good suggestion. We now did new recordings and analysis and showed the DRN cluster stability over 1hr recordings, now presented in **Supp Figure 1D**.

- To mitigate the risk of confounding spatial patterns with movement artifacts, more thorough documentation of raw data is recommended. For instance, the authors could provide supplementary material, such as traces of the movement correction parameters, indicating whether these occur on a different time scale than the activity. Additionally, an example video of the movement-corrected data, along with the corresponding activity response spatial pattern, could be shared.
Response 66: We now provided a supplemental Video, showing movement correction traces, neural activity, behavioral traces. Please find this in **Supp Video S1**.

Figure 2:
- Fig 2D: In the heatmap plot, it is unclear whether the cells are absent or zero; displaying the contour in the image could provide an understanding of the proportion of cells responding. Additionally, if non-responsive cells are found in the region, together with responsive ones, this would help exclude movement artifacts.
Response 67: We now provided these contour lines in all relevant figures.

Figure 3:
- Fig 3A: It would be more helpful to the reader to show the 2-photon micrographs that were used for the rest of the figure, or clarify why the authors use confocal images here.
Response 68: We now provided images collected by two-photon microscopy, during same experiments analyzed for this study.

- Fig 3C: Cell assignment can be noisy due to resolution and expression variability. Explaining and maybe quantifying uncertainty in cell type assignment would help clarify if the variability across fish is biological or due to measurement.
Response 69: The red pixel intensities were z-scored for each animal to ensure possible variabilities across animals do not affect our Gad1b neuron identification. We also now provide the distribution of our z-scored red-pixel values of all DRN neurons measured for this figure, as well as highlighted the z-score cut-off value that is used for

determining Gad1b positive DRN neurons (**Figure 3C**). We should also note that our conclusions remain unchanged, when other cut-off values were tested.

- Fig 3H: Please clarify how the control was done (whether it was gad1b cell correlation with non-gad1b cells or correlation between non-gad cells).
Response 70: This figure panel is now Figure 3I. The controls were correlations of Gad1b cells with non-Gad1b DRN neurons. This is now clarified better in the text.

Figure 4:
- Please clarify whether the statistics are done on voxels or components.
Response 71: This is calculated on voxels. This is now clarified better in the text.

- Fig 4G: Details on how the alignment between animals was made are missing. Is there a reference image channel, or did the authors just map spatially filtered versions of the axonal structures on top of each other? If the latter case, how precise would that be?
Response 72: There is no reference brain atlas for juvenile zebrafish. Since the positioning and size of brains were very similar across animals and recordings, we have done translational alignment and overlaid data from multiple animals. In brief, dorsal-ventral, antero-posterior and medio-lateral edges of relevant brain regions (DRN or forebrain) were determined and matched across animals. We did not aim at aligning individual axons or neurons, which was not needed in our claims and conclusions.

- Fig 4H: a side projection would be helpful to visualize segregation in depth.
Response 73: Such sagittal views are now provided in **Figure 4J,R,Z**.

Figure 5:
- Fig 5C,D: is there a mistake in the little drawing? Should this be a tail flick rather than a tap? In the legend, it says 'tail-beat responses'.
- It would be useful to also show traces from the forebrain neurons for direct comparison with the axons.
Response 74: We thank for noting this; indeed, the presented results were vibration evoked locomotor response. To be in line with the rest of our study, and to also address reviewers' comment regarding the analysis of spontaneous locomotion period. We now did a whole new set of experiments, where we analyze animals' spontaneous locomotor tail-bouts and presented these new results. Our results and conclusions with spontaneous locomotion (**now in updated Figure 5**) are in line with our previous results with vibration evoked locomotion (**which is now moved to Supplemental Figure7K-O**). These new data and findings strengthen the relationship between raphe axons in the telencephalon and telencephalic neuron activity. We also showed the responses of forebrain neurons for direct comparison in **Supp Figure 7A-B**.

Figure 6:
- Fig 6C: It is important to clarify whether the decrease in correlation between regions can be trivially attributed to their reduced activity. For the example, the authors could calculate the correlations for the activity of the control but scaled down to approximately the value found after the ablation.

Response 75: This is such a good idea. We thank the reviewer #4 for this suggestion regarding subsampling of forebrain neurons with similar sensory responses and testing the effect of DRN ablation on their synchrony. We now did this test and presented our results in **Supp Figure 8A-D.** Indeed, even after subsampling neurons with statistically similar responses, we still observed that within and across forebrain region correlations are significantly reduced after DRN ablation. We hope that our new controls make a more convincing point about the reduction in forebrain synchrony in DRN ablated animals. We now updated our results section accordingly.

- Fig 6J: There are more than 16 points, are these really one point per fish? Or are these behavior trials?

Response 76: Reviewer 1 suggested an excellent analysis to improve these plots, not only region by region but neuron by neuron, hence we now present the slope of the relationship between the activity of individual neurons and animals locomotor behavior in this new updated figure. While our results remain unchanged, up on this new and more fine-grained analysis, differences between control and DRN ablated groups are prominent. A detailed explanation of these new results can also be found in **Response 6 (above).**

Figure 7:
- The author demonstrates in the initial section of the work that there are heterogeneous populations of tph-positive neurons involved. However, these populations are manipulated collectively in the behavioral experiments. This aspect requires further discussion.

Response 77: This is a good point. We now adjusted our discussion to reflect the limitations of entire chemogenetic DRN ablation while discussing our behavioral results.

Discussion:
- There has been a lot of recent data on the activity of the raphe in fish, it would be great to have a bit more detailed interpretation of the results in the context of these other studies (eg. psilocybin, behavior states).

Response 78: We have done our best to cite all relevant papers of DRN in fish. We now discussed few additional references in the revised discussion.

Reviewer #4 (Remarks on code availability):

code access not available.

Response 79: We apologize that the codes were in the link, but the link was not made public. We now enable public sharing of the codes**.**

Dear Editors and Reviewers,

We thank you all for your thoughtful feedback and support for our manuscript.

As recommended by the reviewers and the editor, we have revised our manuscript to adjust the tone of our claims and to explicitly mention some of the limitations. All changes in the manuscript are highlighted in yellow.

All reviewer comments are presented below in **red**, and our responses in **black** letters.

We look forward to your feedback.

With kind regards,

Emre Yaksi Aytac Kadir Mutlu

REVIEWER COMMENTS

Reviewer #1 (Remarks to the Author):

I thank the authors for carefully engaging with my comments and suggestions. I believe that the additional analyses and clarifications have increased the rigor of the manuscript substantially. I think that the claims currently made in the manuscript are supported by the data as presented. While I agree with reviewer #3 that experiments to link the different described phenomena (spatial organization, effects on forebrain activity, behavioral effects of DRN ablations) are lacking, I do not get the impression from reading the paper that these connections are implied. However, it might be useful if the authors explicitly acknowledge that DRN neurons project broadly throughout the brain and that future work will be necessary to tease these influences apart.

Response #1: We thank Reviewer #1 for the constructive feedback, which has significantly strengthened our manuscript. We also appreciate the reviewer's supportive remark that "**the claims currently made in the manuscript are supported by the data as presented**". As recommended, we have now added a section to the Discussion explicitly acknowledging that "**DRN neurons project broadly throughout the brain and we did not dissect the individual contributions of these different DRN projections, which is a limitation of our study. Future work will be necessary to tease apart the influences of different DRN subpopulations and their projections on brain computations and animal behaviour**".

Reviewer #1 (Remarks on code availability):
The code is now fully available; however, I have not reviewed it since I do not have MATLAB installed.

Response #2: Thank you. As reccomended by the reviewer #4, we also now improved the "read me file".

Reviewer #3 (Remarks to the Author):

The authors have made some real attempts to improve the manuscript, such as the response-amplitude-matched subsampling for correlation analyses or the variance-explained analysis for locomotion.
Response #3: We thank the reviewer for all the constructive feedback, and we appreciate the acknowledgment that we did our best to implement all reviewer comments, which has improved our manuscript.

However, a fundamental problem persists. The authors' rebuttal claims they never asserted that DRN-forebrain interactions underlie the observed behavioral phenotypes, yet the manuscript's title, structure, and framing continue to strongly imply exactly this causal chain. Until this disconnect is resolved, the manuscript cannot be recommended for publication.

In the rebuttal, the authors make the following statements:
"We did not intend to make any claim that 'DRN-forebrain interactions' are responsible for the behavioral results in Figure 7."
"Given that we did not make such a claim, we hope that reviewer #3 will agree that proving a claim that we did not make (on specific impact DRN to forebrain connections in animal behavior) is not within the scope of this study."
"Specific ablations of DRN to forebrain connections while performing behavioral tests in free-swimming animals, is not experimentally feasible ... Hence testing the specific impact of DRN to forebrain connections in free-swimming behaviors in zebrafish is not a technically feasible experiment."

I accept the authors' assertion that they cannot perform the pathway specific ablation experiment needed to substantiate the title of this paper "Topographically organized dorsal raphe activity modulates forebrain sensory-motor computations and defensive behaviors". However, ablation of the forebrain itself has been established in previous publications.

But the bigger concern is that the authors deny making a claim while simultaneously preserving all the textual and structural elements that lead a reader to infer precisely that claim.
Response #4: We thank the reviewer for these thoughtful feedback. As also agreed by Reviewer #1, we did not claim a causal relationship between forebrain activity and the behavioral effects of DRN ablations. We argue that the order of the presentation of our results is a logical structure for communicating the findings of our study. Following the advice of all reviewers and the editors, we hope that the additional adjustments we made in our updated manuscript highlight the limitations of our study and sufficiently address the remaining concerns of Reviewer #3. You will find our point-by-point responses below.

Specifically:
1. The title implies a causal chain through the forebrain
The current title is: “Topographically organized dorsal raphe activity modulates forebrain sensory-motor computations and defensive behaviors.” The syntactic structure of this title is clearly making an effort to imply that the DRN’s modulation of forebrain activity is the mechanism through which defensive behaviors are affected. If the authors genuinely believe that the behavioral effects may arise from DRN projections to any brain region (tectum, hindbrain, etc.), the title must be revised to reflect this.
Response #5: We have revised the title to: “**Topographically organized dorsal raphe activity modulates forebrain sensory-motor representations and contributes to defensive behaviors**”. This title does not make any claim about a causal relationship between forebrain activity and defensive behaviors. Instead, it reflects the three core findings supported by our data:
(1) dorsal raphe activity is topographically organized,
(2) dorsal raphe ablation modulates forebrain activity, and
(3) dorsal raphe ablation alters defensive behaviors.
Importantly, the revised title avoids implying that the behavioral effects necessarily occur via the forebrain.
2. The manuscript structure implies a causal narrative
The paper is organized as a sequential argument: 1) DRN is topographically organized, 2) DRN axons modulate forebrain activity topographically, 3) DRN ablation disrupts forebrain synchrony and sensory-motor responses, 4) DRN ablation amplifies behavioral responses in head-restrained fish, 5) DRN ablation amplifies defensive behaviors in freely swimming fish. A reader following this narrative naturally concludes that the behavioral effects in steps 4–5 arise through the forebrain mechanisms documented in steps 2–3. This inference is reinforced by placing the head-restrained behavioral result within the same figure as the forebrain data, despite these being, accordingly to the authors, logically independent observations.
Response #6: We collected the head-restrained behavioral results simultaneously with the forebrain data. Hence, it is natural and logical to present these simultaneously measured observations in the same figure. Up on reccomendation of Reviewer #3, we have now toned down our claims at the end of this result section (Figure 6) by updating the former statement, “**Taken together, our results highlight the importance of DRN in regulating forebrain activity and shaping the relationship between forebrain activity and animals’ locomotor responses to mechanical vibrations**” with the simpler statement, “**Taken together, our results highlight the importance of DRN in regulating forebrain activity**”.

3. Key text in the Discussion still implies the link
The Discussion states: “We propose that locomotor signals from DRN can be utilized to modulate forebrain activity. Our results revealed that such DRN inputs amplify forebrain responses to sensory cues such as mechanical vibrations, as we observed in our experiments (Figure 6F-H). This can be particularly important during defensive behaviors, where animals make risk-benefit analyses. In fact, we observed that ablating DRN amplify lasting defensive behaviors after a transient threat.” This passage directly links the forebrain modulation to defensive behaviors, which is the claim the authors

repeatedly insist they are not making. Although the authors added a caveat in the following paragraph acknowledging that DRN innervates the entire brain, the rhetoric of the passage above substantially outweighs it.
Response #7: We now updated this section as below to better highlight the limitation of our study and the need for future experimentation. “**We propose that locomotor signals from DRN can be utilized to modulate forebrain activity and amplify forebrain sensory responses. While we also observed that broad DRN ablation amplifies defensive behaviours, the specific role of DRN to forebrain projections in defensive behaviours is yet to be determined. For example, DRN neurons project broadly throughout the brain, and we did not dissect the individual contributions of these different DRN projections, which is a limitation of our study. Future work will be necessary to tease apart the influences of different DRN subpopulations and their projections on brain computations and animal behaviour**”.

As I mentioned in the original review, there are two disconnected stories presented in the paper. The authors have not attempted to address, or even acknowledge, the problem associated with this.
Response #8: We hope that the specific adjustments that we made in our results and discussion sections, which are described above, now better address the comments of Reviewer #3.

Reviewer #4 (Remarks to the Author):
This revision marks a notable improvement. The authors have thoroughly responded to the primary issue of differentiating between behavioral and stimulus responses, and they have also resolved all my minor concerns.
Response #9: Thank you for all the constructive feedback, which helped us to improve our manuscript. We are glad to hear that Reviewer #4 is satisfied with our thorough responses to their major and minor comments.

However, one issue still remains: The micrograph in Fig. 5 shows that resolution does not clearly separate axons from cell bodies, as they are in contact in both xy and likely z dimensions. It may be that axons from the raphe are located on top of the imaged nuclei (of course, not inside). As such, the imaging of these nuclei may contain, in fact, also signals from the raphe axons, potentially inducing spurious correlations. In addition, several axons, including those distant from most segmented axons, are still unsegmented, while some segmented cells resemble axon boutons. In principle, their correlation analysis is a great idea to probe for functional connectivity, but these issues make the claims about raphe axon and forebrain neuron correlations and the general approach not fully convincing. I would recommend that the authors clearly state this limitation in their results as well as address this point in their discussion. If the authors decide to drop this analysis, this would also be OK.
Response #10: As suggested by Reviewer #4, we have now clearly stated this limitation in our results and discussion sections, in the following sentences. RESULTS:“ **Despite the limitation that a small amount of DRN axons might spatially overlap with forebrain neuron nuclei, our results indicate that activity of DRN axons and forebrain neurons covary during locomotion, vibration responses and ongoing**

activity". DISCUSSION: "**We acknowledge that such covariance might be affected by a small amount of DRN axons spatially overlapping with forebrain neuron nuclei, potentially limiting the interpretation of these correlations**".

Reviewer #4 (Remarks on code availability):
I have checked the GitHub repository, and it looks quite minimal. I have not tried to run the code. It would be nice if the authors could expand their readme file a bit to explain the structure of the codebase and how to download the processed data. Documenting MATLAB files will also help readers to understand the analysis.
Response #11: As requested, we have now expanded the README file to better explain the structure of the codebase. Moreover, we documented the MATLAB scripts used in the analysis by adding code header, comments, and usage instructions. We should also add that, upon acceptance of our manuscript, we will upload our relatively large dataset to an open-access server and provide the DOI number. This is the procedure we followed in our previous paper published in Nature Communications, and we hope that this is acceptable to the reviewers and the editor.

Reviewer #5 (Remarks to the Author):
I co-reviewed this manuscript with one of the reviewers who provided the listed reports. This is part of the Nature Communications initiative to facilitate training in peer review and to provide appropriate recognition for Early Career Researchers who co-review manuscripts.
Response #12: Thank you, we appreciate this initiative.
